# Efficient Induction of T Cells against Conserved HIV-1 Regions by Mosaic Vaccines Delivered as Self-Amplifying mRNA

**DOI:** 10.1016/j.omtm.2018.10.010

**Published:** 2018-10-26

**Authors:** Nathifa Moyo, Annette B. Vogel, Søren Buus, Stephanie Erbar, Edmund G. Wee, Ugur Sahin, Tomáš Hanke

**Affiliations:** 1 The Jenner Institute, Nuffield Department of Medicine, University of Oxford, Oxford OX3 7DQ, UK; 2 Biopharmaceutical New Technologies (BioNTech) Corporation, Mainz 55131, Germany; 3 Department of Immunology and Microbiology, University of Copenhagen, Copenhagen 2200, Denmark; 4 International Research Center for Medical Sciences, Kumamoto University, Kumamoto 860-0811, Japan

**Keywords:** RNA vaccine, HIV vaccine, conserved regions, tHIVconsvX, simian adenovirus, MVA, T cell vaccine, heterologous prime-boost, *in vivo* killing, T cells

## Abstract

Focusing T cell responses on the most vulnerable parts of HIV-1, the functionally conserved regions of HIV-1 proteins, is likely a key prerequisite for vaccine success. For a T cell vaccine to efficiently control HIV-1 replication, the vaccine-elicited individual CD8^+^ T cells and as a population have to display a number of critical traits. If any one of these traits is suboptimal, the vaccine is likely to fail. Fine-tuning of individual protective characteristics of T cells will require iterative stepwise improvements in clinical trials. Although the second-generation tHIVconsvX immunogens direct CD8^+^ T cells to predominantly protective and conserved epitopes, in the present work, we have used formulated self-amplifying mRNA (saRNA) to deliver tHIVconsvX to the immune system. We demonstrated in BALB/c and outbred mice that regimens employing saRNA vaccines induced broadly specific, plurifunctional CD8^+^ and CD4^+^ T cells, which displayed structured memory subpopulations and were maintained at relatively high frequencies over at least 22 weeks post-administration. This is one of the first thorough analyses of mRNA vaccine-elicited T cell responses. The combination of tHIVconsvX immunogens and the highly versatile and easily manufacturable saRNA platform may provide a long-awaited opportunity to define and optimize induction of truly protective CD8^+^ T cell parameters in human volunteers.

## Introduction

Control of the HIV-1 epidemic remains one of the leading global health priorities. Remarkable gains have been achieved in decreasing HIV-1 transmission and AIDS-related deaths due to development of over 30 antiretroviral drugs.^1^ However, still almost half of people who are HIV-1 positive are unaware of their status. In addition, antiretroviral drugs are not available on a regular reliable basis in many resource-poor settings, their effective administration requires rigorous daily compliance,^2^, ^3^ there are toxicities associated with their long-term use,^4^, ^5^, ^6^ and viruses develop resistance. Also, there is unwillingness to take drugs in a surprisingly large proportion of infected e.g., adolescent, individuals. Thus, an effective, prophylactic HIV-1 vaccine will always be the best solution and possibly key to any strategy for halting the AIDS epidemic.^7^ For the most efficient control of HIV-1, a vaccine will likely have to induce both broadly neutralizing antibodies and effective CD8^+^ T cells.^8^ Our aim is to understand and induce protective T cell responses, which will have a role in control of HIV-1 following initial transmission and in HIV-1 cure.

We have pioneered a T cell vaccine strategy, which employs highly conserved regions of the HIV-1 proteome.^9^ The first-generation immunogen HIVconsv uses 14 regions designed as a clade-alternating consensus.^10^ HIVconsv was tested extensively in pre-clinical settings.^11^, ^12^, ^13^, ^14^, ^15^, ^16^, ^17^, ^18^, ^19^ To date in regimens involving plasmid DNA, simian (chimpanzee) adenovirus (ChAdV-63), and poxvirus-modified vaccinia virus Ankara (MVA), the HIVconsv vaccines have been tested in eight clinical trials, showed promising immunogenicity and *in vitro* control of replication of four major clades of HIV-1 and, in combination with latency-reverting agent, produced a signal of viremic control during monitored antiretroviral treatment (ART) pause in early treated patients (Fidler et al., 2018, Intern. AIDS Soc., abstract; Mothe et al., 2017, Intern. Antivir. Soc., abstract; B. Mothe, C. Manzardo, A. Snachez-Bernabeau, P. Coll, S. Moron-Lopez, M.C. Puertas, M. Rosas, P. Cobarsi, R. Escrig, N. Perez-Alvarez, I. Ruiz, C. Rovira, M. Meulbroek, A. Crook, N. Bothwick, E.G. Wee, H. Yang, J.M. Miró, L. Dorrell, B. Clotet, J. Martinez, Picado, C. Brander, and T.H., unpublished data).^20^, ^21^, ^22^, ^23^, ^24^, ^25^ Six immunogens of the second generation, collectively designated tHIVconsvX, further improved the first-generation conserved-region design by bioinformatics-assisted definition of conserved regions, including protective epitopes defined in patient cohorts on four continents and maximizing a perfect potential T cell epitope match of the vaccines to the circulating global HIV-1 isolates through using a bivalent mosaic.^26^ The second-generation immunogens delivered by DNA, ChAdOx1, MVA, and integration-defective lentivirus vectors demonstrated good immunogenicity in animal models,^26^, ^27^ and recombinant ChAdOx1 and MVA are in the pipeline to enter human trials.

It is our belief that eventual development of effective vaccines against HIV-1 is more likely to happen through iterative multiple small but significant steps forward rather than a new “out-of-box” idea. The most relevant developments will always be those made in human trials, where acceleration of iterative improvements will be greatly facilitated by easily adaptable, affordable, and quickly manufacturable vaccine modalities. One such vector currently in the spotlight is mRNA. The use of naked RNA molecules was hampered for a long time by its instability, inefficient crossing of the cell membrane, and potent induction of innate responses, which, e.g., cease cellular translation.^28^, ^29^ Over the last decade, there have been great leaps toward solving these challenges through structural and chemical modifications to the RNA molecule itself,^30^, ^31^, ^32^, ^33^, ^34^, ^35^, ^36^ formulation into various nanoparticles or nanoemulsion,^33^, ^37^, ^38^, ^39^ and use of polymers and conjugation.^40^, ^41^ These advances enhanced by the excellent safety features of mRNA vaccines, and their fully synthetic and relatively cheap, fast, and scalable GMP manufacture have generated lots of hopes and indeed investment into this emerging platform.^42^ mRNA vaccines in pre-clinical models showed protective efficacy against a number of viruses, such as influenza, rabies, Ebola, and Zika,^37^, ^39^, ^43^ and an increasing list of preventive and therapeutic vaccine candidates have entered clinical evaluation in humans for cancer, allergy, and infectious diseases.^44^ Although mRNA vaccines to date focused not exclusively but predominantly on induction of neutralizing antibodies, in the present work, we explore the potential of polymer-formulated self-amplifying mRNA (saRNA) vaccines to induce alone and in a combination with other vaccine vectors CD8^+^ T cell responses. The results are discussed in the context of the current state of HIV-1 vaccine development.

## Results

### Design and Construction of the AIR.tHIVconsv1 and AIR.tHIVconsv2 Candidate Vaccines

**Figure 1:**
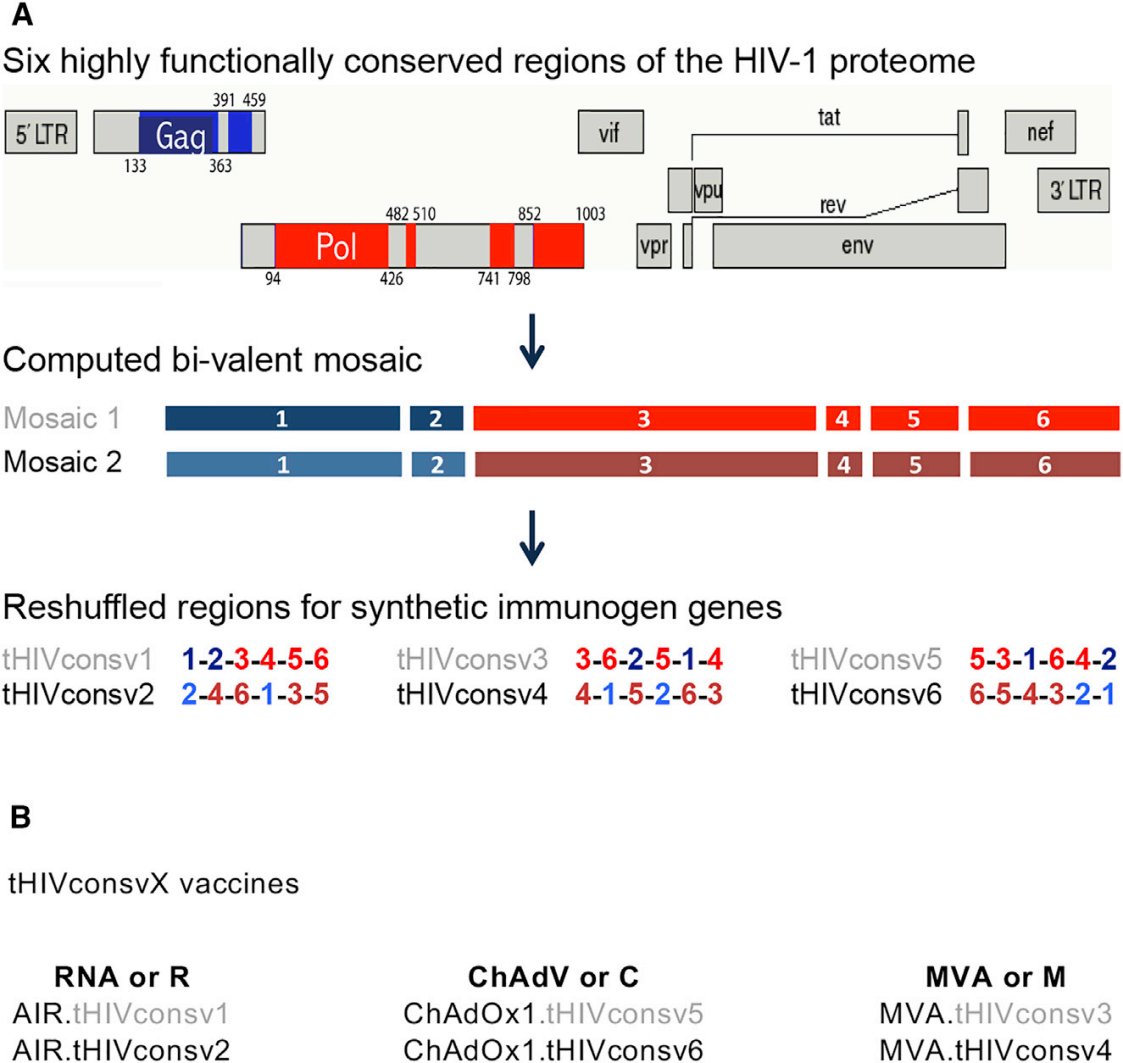
The tHIVconsvX Vaccines (A) Curated full-protein amino-acid HIV-1 sequences present in the LANL-HSD database (September 2013) were used to compute bivalent mosaic and select 6 highly conserved regions of the HIV-1 proteome.^26^ Mosaic 1 and mosaic 2 (color-coded) differ in approximately 10% of amino acids and together maximize match of the vaccines to globally circulating HIV-1 group M isolates. Each tHIVconsvX vaccine component of the multi-component regimens uses a different order of the 6 conserved regions to minimize induction of T cell responses to potential neoepitopes irrelevant for HIV-1 generated by two juxtaposed regions.^20^ (B) Vaccines used in this work. The 6 differentially ordered transgenes were inserted into vaccine vectors saRNA (RNA or R), non-replicating simian adenovirus ChAdOx1 (ChAdV or C), and non-replicating poxvirus (MVA or M). Unless specified, for each immunization, both mosaics were used together.

Novel candidate HIV-1 vaccines were vectored by a self-amplifying RNA-based Ribological RNA amplicon derived from Semliki Forest virus (SFV),^45^ which was developed by BioNTech and designated Amplified Immune Response (AIR). AIR.tHIVconsv1 and AIR.tHIVconsv2 mRNA vaccines express the second-generation conserved-region immunogens tHIVconsv1 and tHIVconsv2, respectively (Figure 1). These immunogens consist of 6 highly conserved regions of the HIV-1 Gag and Pol proteins computed into a bivalent mutually complementing mosaic 1 and mosaic 2,^26^ which are two versions of the same regions differing in approximately 10% of amino acids (Figure 1A). Mosaic 1 (tHIVconsv1&3&5) and mosaic 2 (tHIVconsv2&4&6) are always co-administered together for each vaccine dosing, whereby any mosaic 1 can pair with any mosaic 2 without compromising the regimen’s immunogenicity. Note that the choice of tHIVconsvX immunogens for vectors was arbitrary. tHIVconsv5&6 and tHIVconsv3&4 were inserted into ChAdOx1 and MVA, respectively^26^; thus, tHIVconsv1&2 were used for other vaccine modalities (Figure 1B). All genes coding for the tHIVconsvX immunogens employ humanized codons. After a completely cell-free *in vitro* transcription, the saRNA was purified and polymer formulated for enhanced cellular delivery and translation rate. The construction of the viral vaccines was described previously.^26^

### saRNA Vaccine Dose Optimization for T Cell Induction

**Figure 2:**
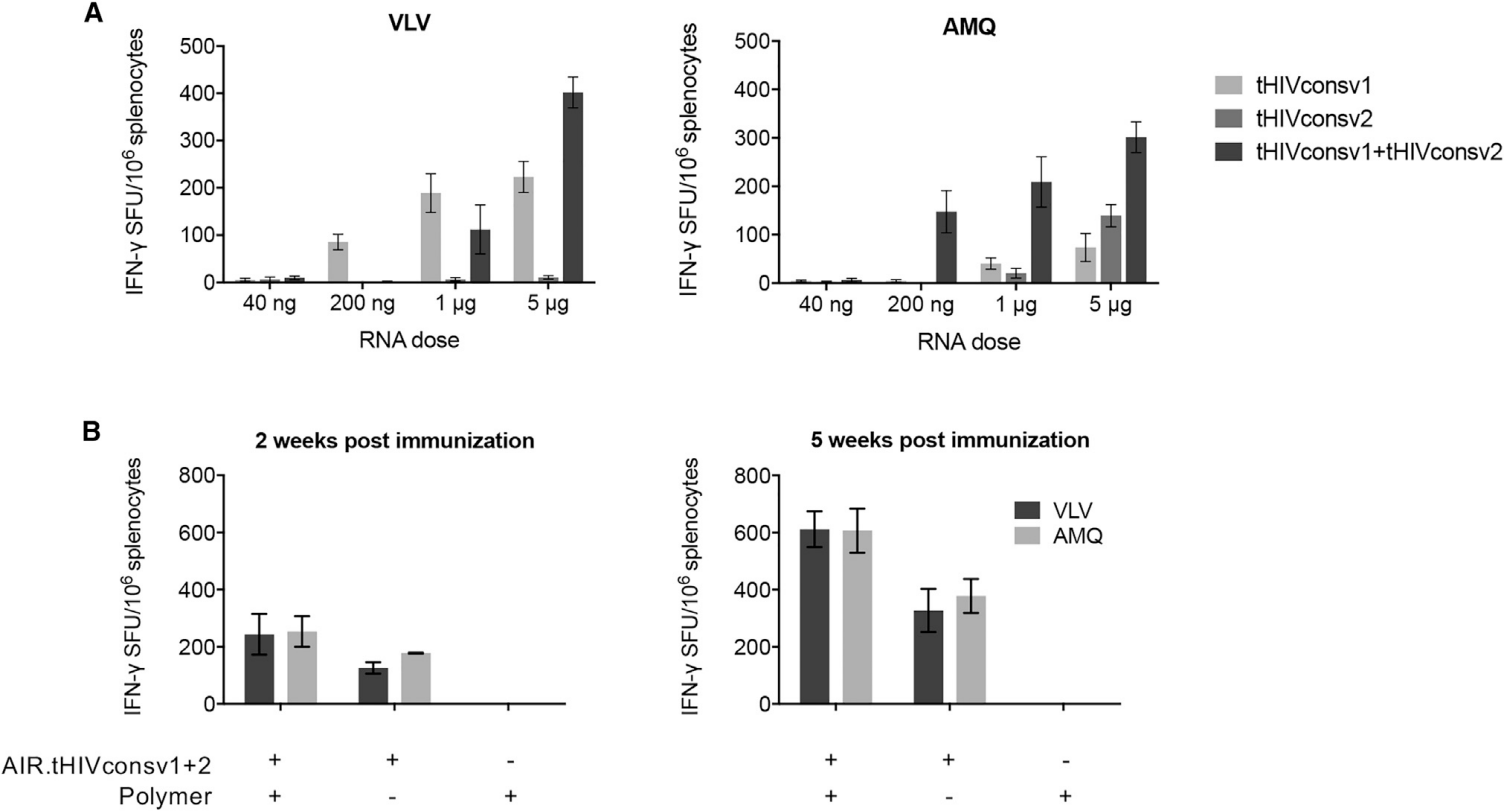
Dose Response of Mosaic saRNA Vaccines (A) Groups of BALB/c mice were administered increasing vaccine doses of either individual mosaic vaccines or their half-dose combination. (B) Effect of saRNA formulation. A single saRNA dose of AIR.tHIVconsv1 + AIR.tHIVconsv2, 5 μg in total, was administered to mice with or without polymer and also polymer alone was used to assess the immunogenicity of each vaccine component. Mice were sacrificed either 2 or 5 weeks after vaccination (Figure S1). For both panels, vaccine-elicited T cell responses in the spleen were enumerated in an IFN-γ ELISPOT assay, performed in triplicate, using two immunodominant peptide pairs VLV and AMQ; the mean of the triplicates for each mouse was calculated. Data are shown as median ± interquartile range (IQR) of the means for n = 3 mice per group.

All vaccine dosings were delivered by intramuscular needle injection into the quadriceps muscle of the hind legs. BALB/c mice were injected with a total of 40 ng, 200 ng, 1μg, and 5 μg of saRNA of either AIR.tHIVconsv1 alone, AIR.tHIVconsv2 alone, or as two half-doses of AIR.tHIVconsv1 + AIR.tHIVconsv2 together. We took advantage of two known H-2^d^ immunodominant epitopes AMQ (AMQMLKD/ETI; pool P1) and VLV (VLV/IGPTPVNI; pool P4)^17^, ^27^ and used only those two peptide pairs, one from each mosaic, in an interferon γ (IFN-γ) ELISPOT assay for enumeration of vaccine-elicited responses. We found that, 1 week after administration, the saRNA vaccines induced only weak responses below 50 spot-forming units (SFUs)/10^6^ splenocytes (Figure S2). It was through analysis of the kinetics of the T cell response induction shown below that we realized that, following saRNA vaccination, T cell frequencies keep increasing beyond the first week, which is the peak time for many viral vectors. Thus, the same dose-response experiment was repeated, but this time, mice were sacrificed 5 weeks post-vaccination. First, we confirmed the immunogenicity of both AIR.tHIVconsv1 and AIR.tHIVconsv2 individually (Figure 2A). For the combined half-doses, the frequencies of specific T cells were increasing up to 5 μg dose, and the total of 5 μg of saRNA (2.5 μg AIR.tHIVconsv1 + 2.5 μg AIR.tHIVconsv2) was chosen as the standard intramuscular dose. Next, we assessed the importance of the saRNA vaccine polymer formulation. Although complexing saRNA with polymer approximately doubled the elicited T cell frequencies relative to naked saRNA and reached approximately 600 IFN-γ SFUs/10^6^ splenocytes for both the VLV and AMQ epitopes at 5 weeks post-administration, the polymer without saRNA failed to induce any HIV-1-specific responses (Figure 2B).

### Characterization of T Cells Induced by a Single saRNA Dose

**Figure 3:**
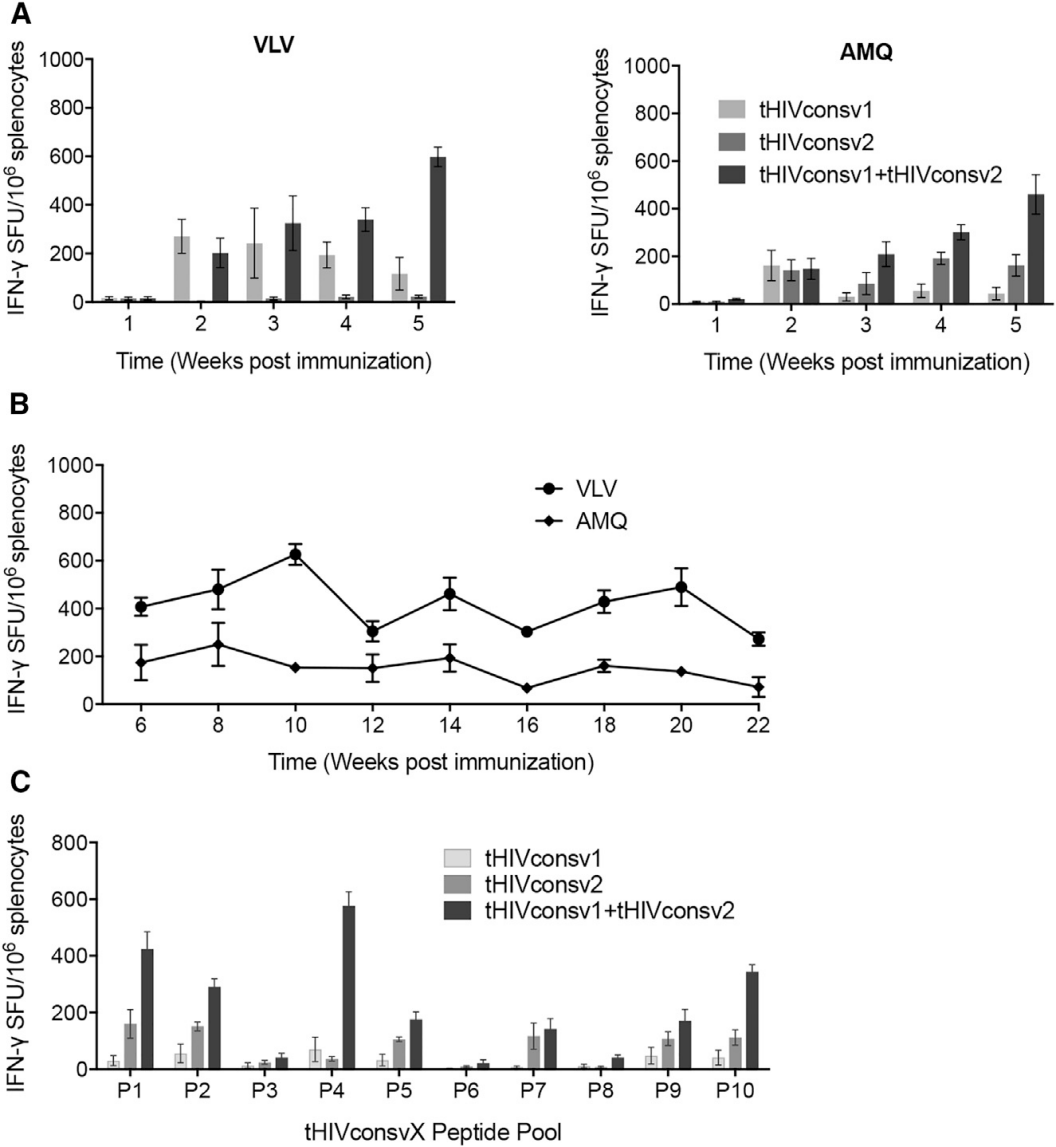
Induction of tHIVconsvX-Specific T Cells by a Single Dose of the saRNA Vaccine Groups of BALB/c mice were immunized with 5 μg of formulated AIR.tHIVconsv1, AIR.tHIVconsv2, or half-doses of AIR.tHIVconsv1 + AIR.tHIVconsv2 (Figure S1), and the vaccine-elicited responses were analyzed using the IFN-γ ELISPOT assay, performed in triplicate; the mean of the triplicates for each mouse was calculated. (A and B) Kinetics of T cell responses between weeks 1 and 5 (A) and weeks 6 and 22 (B) was examined employing two immunodominant peptide pairs VLV and AMQ indicated above the graphs. The latter interval only employed the combined vaccine. (C) Splenocytes from 5 weeks after saRNA vaccination were tested for recognition of ten peptide pools P1–P10 across the entire length of the tHIVconsvX immunogen to provide the total magnitude and initial estimation of the response breadth. All data are shown as median ± IQR of the means for n = 5 mice per group.

**Figure 4:**
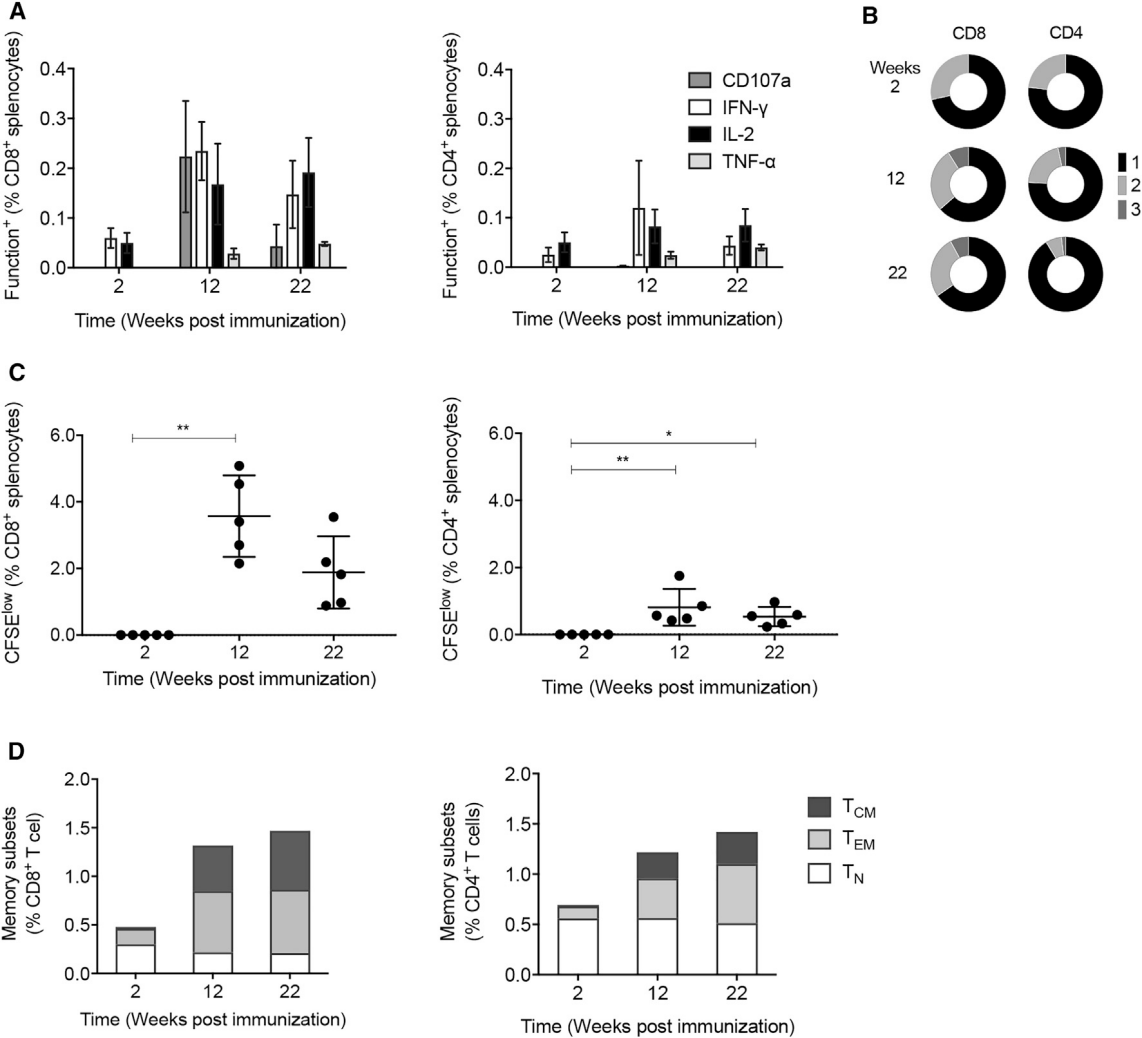
Evolution of saRNA-Induced T Cell Functions and Memory Subtypes Groups of BALB/c mice were immunized with total of 5 μg of formulated AIR.tHIVconsv1 + AIR.tHIVconsv2 (Figure S1), and the vaccine-elicited responses were analyzed using polychromatic flow cytometry at weeks 2, 12, and 22. (A) Total frequencies of cells with indicated functions at 3 time points post-immunization are shown for vaccine-elicited CD8^+^ and CD4^+^ T cells in graphs, and (B) the pie charts provide the plurifunctionality, whereby 1 (black)—one function; 2 (light gray)—two functions; and 3 (dark gray)—three functions (see Figure S5 for the gating strategy). (C) Proliferative capacity assessed in a CFSE-dilution assay (see Figure S7 for the gating strategy) is shown. (D) Memory subtypes in total CD8^+^ and CD4^+^ T cells responding to the BALB/c peptide pool by expression of CD44 and CD62-L (see Figure S6 for the gating strategy) are shown. T_CM_, central memory (CD44^hi^CD62L^hi^); T_EM_, effector memory (CD44^hi^CD62L^lo^); T_N_, naive T cells (CD44^lo^CD62L^hi^). The expression of indicated markers was analyzed in duplicate; the mean of the duplicates for each mouse was calculated. In (A) and (C), data are shown as median ± IQR and in (B) and (D) are shown as the median of the means for n = 5 mice per group. Results were analyzed using the Mann-Whitney U test for comparison between the long-term samples versus the early time point. Significant p values are indicated by asterisks: *p < 0.05; **p < 0.01.

Next, we characterized the T cell responses induced by a single saRNA dose. Groups of BALB/c mice were immunized using 5 μg of saRNA of the single or combined vaccines, sacrificed weekly between 1 and 5 weeks post-delivery, and the vaccine-elicited frequencies were enumerated using the two VLV and AMQ pairs of peptides. First, each mosaic induced different frequencies of VLV- and AMQ-specific CD8^+^ T cells depending on whether it contained immunodominant or subdominant epitope variants. Notably for the late time points, the mixed delivery of AIR.tHIVconsv1 + AIR.tHIVconsv2 was not additive in respect to the T cell frequencies induced by individual mosaics but synergized (Figure 3A). Second, the frequencies of tHIVconsvX T cells increased with time up to 5 weeks post-delivery. This observation prompted an extended experiment, which demonstrated sustained levels of the T cell frequencies following a single vaccine delivery until week 22 (Figure 3B). At week 5, we also assessed the breath of saRNA-induced T cell responses by using 10 peptide pools P1–P10 across the entire length of the two mosaic immunogens and also individual peptide pairs (Figure S3). The saRNA-vaccine-elicited T cells recognized 17 peptide pairs (Figure S4) in 8 peptide pools, confirming induction of broad “immunodemocratic” (broad of similar magnitude) responses (Figure 3C). Next, we examined the evolution of T cell functions at 2, 12, and 22 weeks after a single vaccine administration, assessing upon specific-peptide stimulation the production of IFN-γ, tumor necrosis factor α (TNF-α), interleukin 2 (IL-2), and degranulation measured by surface expression of CD107a, which correlates well with cytolytic activity^46^, ^47^ in an intracellular cytokine staining assay (ICS). All four functions were detectable for CD8^+^ T cells, and CD4^+^ T cells displayed only three functions because they typically do not degranulate (Figure 4A). In broad agreement with the IFN-γ ELISPOT data, the functionality was the highest of the three time points measured at week 12. Putting all these parameters together for single cells, we found over two-thirds of cells to be monofunctional and 8%–9% trifunctional CD8^+^ T cells detected at weeks 12 and 22 (Figure 4B). Both CD8^+^ and CD4^+^ T cells showed no proliferation at 2 weeks, peaked at 12 weeks post-vaccination, and declined thereafter (Figure 4C). Finally, at the same late time points, both CD8^+^ and CD4^+^ T cells showed structured memory phenotypes with significant proportions of effector memory (CD44^hi^CD62L^lo^) and central memory (CD44^hi^CD62L^hi^) T cells and, for CD8^+^ T cells, a smaller fraction of naive T cells (CD44^lo^CD62L^hi^; Figure 4D). Thus, the AIR.tHIVconsv1 + AIR.tHIVconsv2 saRNA vaccines induce conserved region-specific CD8^+^ and CD4^+^ T cells with desirable phenotypic properties.

### Optimal Timing for Homologous saRNA Boost

**Figure 5:**
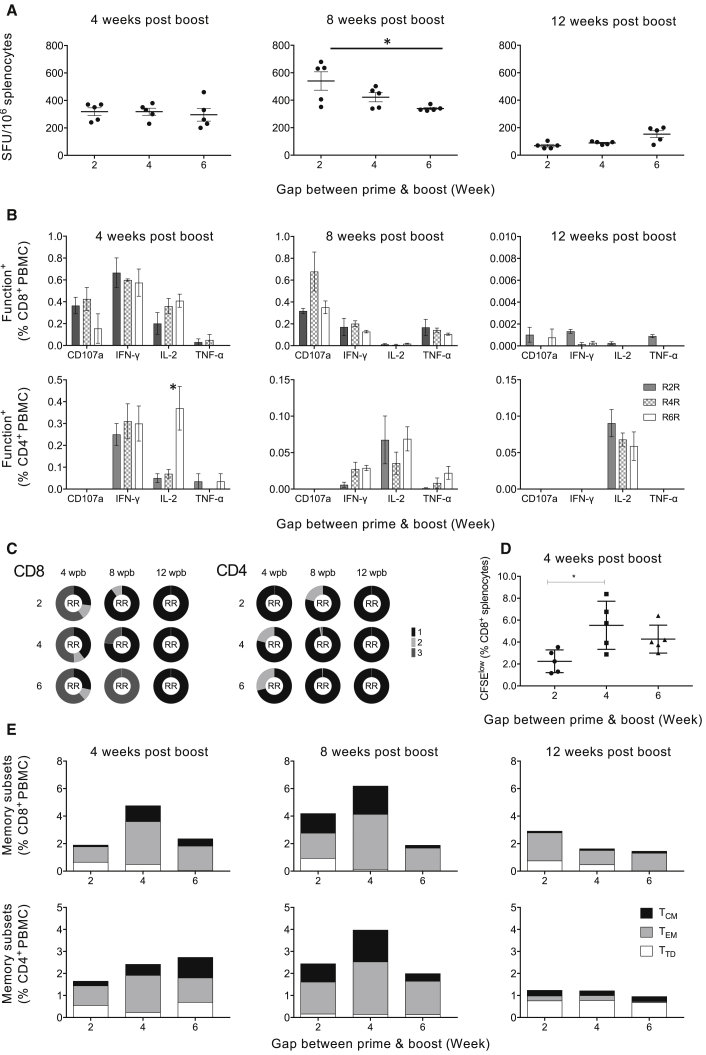
Optimizing Repeated saRNA Vaccine Boost Groups of BALB/c mice were immunized with 5 μg of formulated AIR.tHIVconsv1 + AIR.tHIVconsv2 (RNA or R) and boosted at 2, 4, or 6 weeks later with the same dose of the same vaccines. At 4, 8, and 12 weeks post-boost (Figure S1), the frequencies of responding PBMC and/or splenocytes were determined using one BALB/c pool of 17 responder peptide pairs in (A) an IFN-γ ELISPOT assay performed in triplicate and (B–E) multicolor flow cytometry analysis performed in duplicate; the mean of the triplicates or duplicates for each mouse was calculated and a panel of mAbs (B) used for the functional phenotypes and (C) the proportion of CD8^+^ T cells (left panel) and CD4^+^ T cells (right panel) expressing multiple functions are shown as pie charts, whereby the expression of one, two, or three markers is represented as 1 (black)—one function; 2 (light gray)—two functions; and 3 (dark gray)—three functions (see Figure S5 for the gating strategy); (D) CFSE proliferation assay and (E) using a panel of mAbs characterizing T cell memory subtypes is shown, whereby T_CM_, central memory (CD44^hi^CD62L^hi^); T_EM_, effector memory (CD44^hi^CD62L^lo^); and T_N_, naive T cells (CD44^lo^CD62L^hi^). Data in (A), (B), and (D) are shown as median ± IQR of the means for n = 5 mice per group, and data in (C) and (E) are represented as median of the means for n = 5 mice per group. Groups were compared using two-tailed Mann-Whitney U tests, and two-tailed p values were used. Significant p values are indicated by asterisks: *p < 0.05.

Given the unusual kinetics of induction and long persistence of the saRNA-elicited T cell responses, we set out to carefully determine the optimal time for a homologous saRNA boost. Groups of BALB/c mice were immunized twice with a 2-, 4-, and 6-week gap between the prime and boost administrations and sacrificed 4, 8, or 12 weeks later. Using the IFN-γ ELISPOT assay and the BALB/c pool of 17 pairs of responder peptides, both splenocytes and peripheral blood mononuclear cells (PBMCs) gave similar results, and there was no significant difference between the T cell frequencies among the three gaps with the exception of week 8 post-administration, whereby the difference between a 2- and 6-week gap reached significance (p < 0.05). For all gaps, specific T cell frequencies declined by 12 weeks post-boost (Figure 5A). This broadly agreed with the frequencies of IFN-γ-producing cells measured using ICS, which again did not differ among the three prime-boost gaps. However, there were peak frequencies of IFN-γ^+^ cells at 4 weeks post-immunization, which declined thereafter (Figure 5B). The differences observed among gaps were in the CD8^+^ T cell CD107a expression, which peaked for a 4-week gap at 8 weeks post-boost. We also noted the generally low production of TNF-α, the promoter of inflammation, apoptosis, and immunity.^48^, ^49^, ^50^ For CD4^+^ T cell IL-2 production, there was a peak for the 6-week gap at 4 weeks after saRNA boost, which was much less pronounced for the shorter gaps (Figure 5B). There were not any obvious differences among the three gaps in the number of functions displayed by individual CD8^+^ and CD4^+^ T cells, with the highest plurifunctionality measured 4 weeks post-vaccination (Figure 5C). The best proliferative capacity was observed for a 4-week gap (Figure 5D). Finally, we determined the structure of memory subtypes. The highest frequencies of T cells were detected for the 4-week gap at 8 weeks after vaccination, reaching 6% and 4% of CD8^+^ and CD4^+^ T cells, respectively, and effector memory T cells were the predominant memory subtype (Figure 5E). Overall, homologous saRNA boosts between 2 and 6 weeks after the saRNA priming induced very similar frequencies and qualities of effector CD8^+^ and CD4^+^ T cells. Possibly the regimen of choice would deliver two saRNA doses 4 weeks apart, and the T cells would be expected to peak between 4 and 8 weeks later.

### saRNA Prime Combines Well with Heterologous Viral Vector Boost

**Figure 6:**
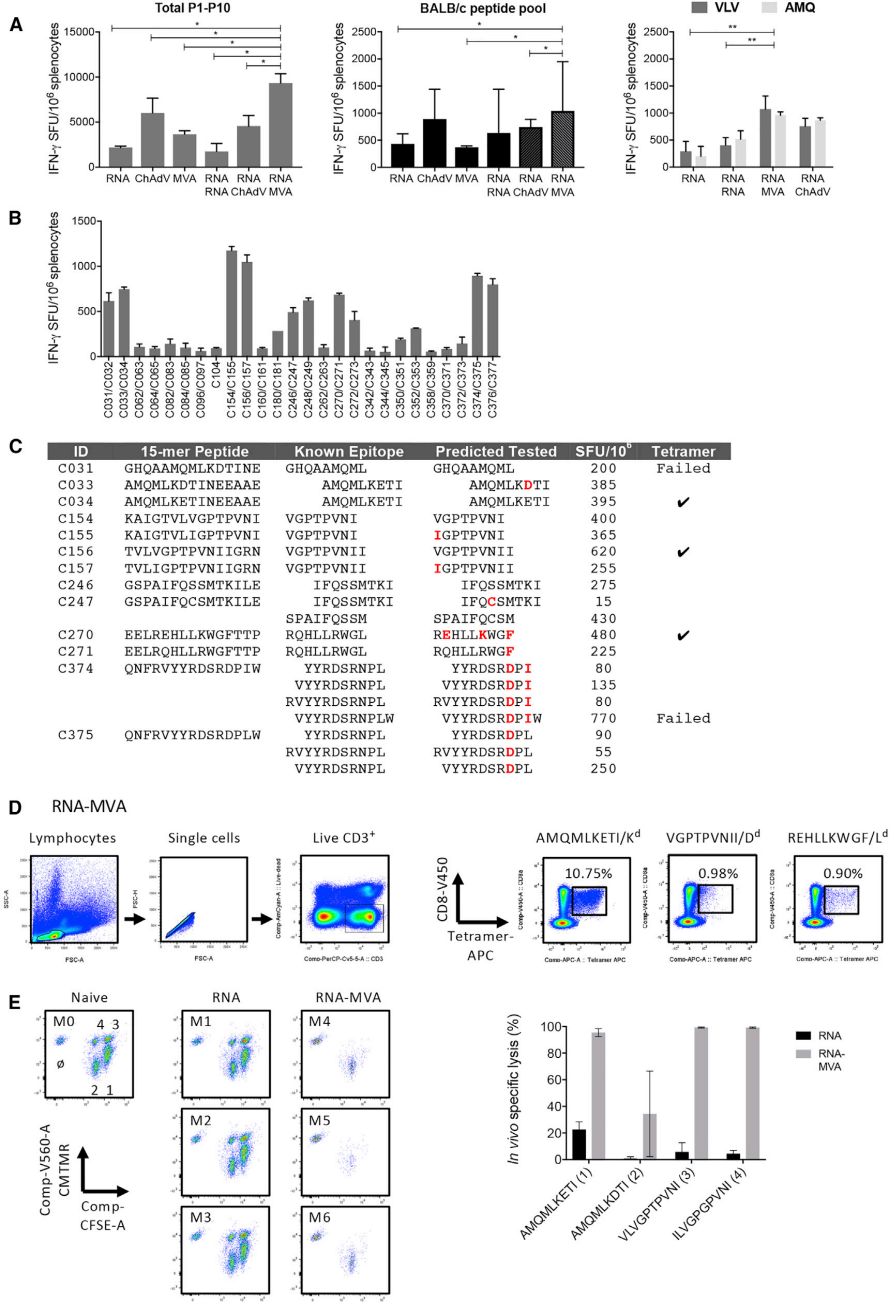
saRNA Vaccine in Heterologous Regimens with Viral Vectors (A) Groups of BALB/c mice were immunized using the indicated regimens (Figure S1). An IFN-γ ELISPOT assay, performed in triplicate, enumerated the elicited T cells using ten overlapping peptide pools P1–P10 and BALB/c pool (left and middle; n = 4 mice) and two pairs of immunodominant epitopes VLV and AMQMLKETI (right; n = 5 mice). The mean of the triplicates for each mouse was calculated. Data are shown as median + IQR of the means of “n” of mice per group. Groups were compared using Kruskal-Wallis test with Dunn’s multiple comparison post-test. Significant p values are indicated by asterisks: *p < 0.05; **p < 0.01. (B) The RNA-MVA regimen was used to assess the breadth of T cell responses by using 201 individual peptide pairs of the P1–P10 pools (Figure S7). Only responses over 50 SFUs/10^6^ splenocytes are measured in duplicate; the mean of the duplicates for each mouse was calculated and are data shown as median + IQR of the means for n = 5 mice per group. (C) Although all 26 stimulatory 15-mer peptides contained minimal epitopes present already in the LANL-HSD, in tHIVconsvX mosaics, 13 of these epitopes had unique amino acids (red) and recognition of 18 9-mers was confirmed. (C and D) For three minimal peptides, their H-2^d^ class I restriction was confirmed by refolding functional MHC class I/peptide monomers. These were assembled into tetramers and used for T cell analysis. (D) (Left) Gating strategy and (right) tetramer reactivity are shown. The best of 4 mice for each tetramer is shown to demonstrate the correct epitope/H-2 class I definition; all four animals had reactive splenocytes. Representative data are shown from a single analyzed mouse out of four mice. Data were acquired in duplicate. (E) *In vivo* killing. (Left) Differentially CMTMR/CFSE labeled and peptide-pulsed target cells transferred into naive (M0), RNA (M1–M3), or RNA-MVA (M4–M6) vaccinated BALB/c mice, re-isolated 10 hr later, and analyzed using flow cytometry are shown. Targets are as follows: 1—AMQMLKETI; 2—AMQMLKDTI; 3—VLVGPTPVNI; and 4—ILVGPGPVNI. (Right) % *in vivo* killing is shown. Flow cytometry plots are shown for all three mice. All data are analyzed in duplicate; the mean of the duplicates for each mouse was calculated and data in graphs shown as median ± IQR of the means for n = 3 mice per group.

Heterologous prime boost combinations of non-replicating subunit genetic vaccines are currently the leading strategies for induction of robust anti-microbial T cell responses in humans.^8^, ^9^, ^51^ Here, single deliveries of saRNA (RNA), ChAdOx1 (ChAdV), and MVA were compared with homologous boost (RNA-RNA) and heterologous boosts with MVA (RNA-MVA) and ChAdOx1 (RNA-ChAdV) delivering the same bivalent-mosaic tHIVconsvX immunogens. IFN-γ ELISPOT assay employing the ten peptide pools P1–P10, BALB/c pool, and immunodominant peptide pairs VLV and AMQ to assess CD8^+^ T cell induction, whereby the VLV/AMQ readout was from separately immunized mice. High frequencies of tHIVconsvX-specific T cells were detected with the RNA-RNA < RNA-ChAdV < RNA-MVA hierarchy peaking for the last at median of 9,343 tHIVconsvX-specific SFU/10^6^ splenocytes (Figure 6A). Thus, the highest frequencies were induced by the RNA-MVA regimen.

### The CD8^+^ T Cell Response Breadth and Specificity

The most efficient regimen for induction of CD8^+^ T cells was AIR.tHIVconsv1 + AIR-tHIVconsv2 prime and MVA.tHIVconsv3 + MVA.tHIVconsv4 boost, or RNA-MVA, and was used to assess the breadth of the responses and map the stimulatory peptides in the BALB/c mice. Overall, responses over 50 SFUs/10^6^ splenocytes were induced to 25 15-mer peptide pairs and one 15-mer common between the two mosaics, which likely corresponded to at least 16 CD8^+^ and/or CD4^+^ T cell epitopes considering the peptide overlap (Figures 6B and S7). Although this analysis confirmed high frequencies of T cells responding to VLV and AMQ, strong responses were also detected to other known epitopes, although many of the epitope variants used here were not previously described (Figure 6C). We used the NetMHCpan 4.0 software to predict optimal binder peptides for the three major histocompatibility complex (MHC) class I H-2^d^ alleles and confirmed recognition of 19 out of 20 in an IFN-γ ELISPOT assay by RM-immune splenocytes (Figure S8). Refolding of stable MHC-peptide monomers confirmed the allele restriction for AMQMLKETI/H-2K^d^, VGPTPVNII/H-2D^d^, and REHLLKWGF/H-2L^d^ and showed tetramer reactivity with RNA-MVA-immune CD8^+^ splenocytes (Figure 6D). Overall, we demonstrated recognition of 11 novel HIV-1-derived epitope variants in the H-2^d^ haplotype. Finally, two variants of the AMQ and VLV epitopes were pulsed individually and the target cells were differentially labeled and transferred back into RNA and RNA-MVA vaccinated animals to assess the cell *in vivo* survival. This assay confirmed *in vivo* cytolytic activity of the vaccine-elicited effectors and indicated superiority of the heterologous regimen and immunodominance of AMQMLKETI over its AMQMLKDTI variant but similarity of the two VLV epitope variants (Figure 6E).

### High Immunogenicity of the RNA-RNA and RNA-MVA Regimens in an Outbred Mouse Stock

**Figure 7:**
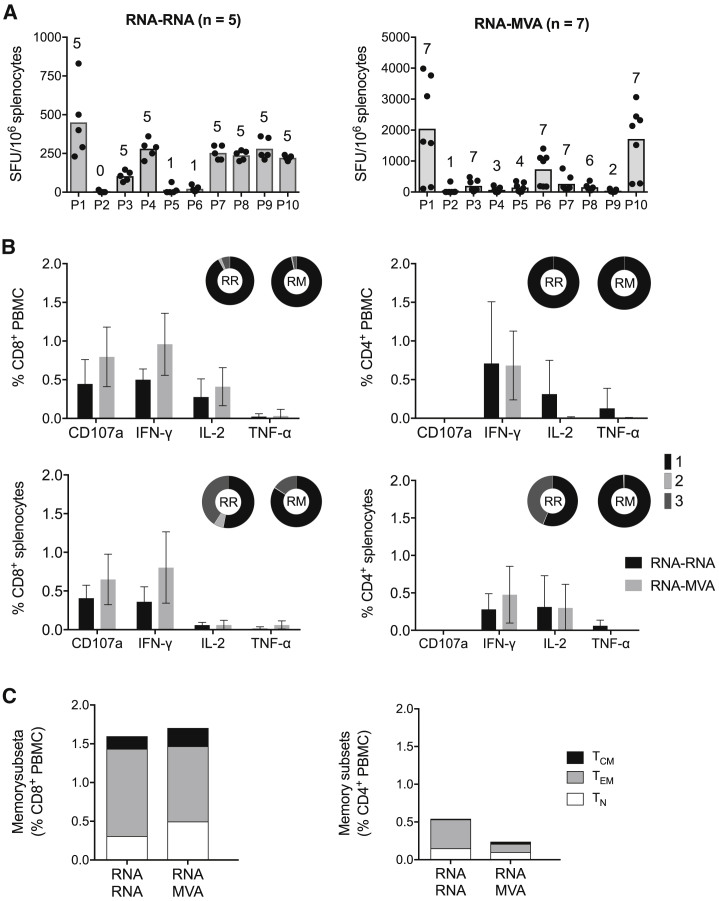
T Cell Induction in an Outbred Mouse Stock CD1-SWISS mice were immunized with 5 μg of formulated AIR.tHIVconsv1 + AIR.tHIVconsv2, boosted at 4 or 2 weeks later with either the same (RR) or with a total of 5 × 10^6^ PFUs of MVA.tHIVconsv3 + MVA.tHIVconsv4 (RM) vaccines and sacrificed at peak responses either at 4 or 1 week later, respectively (Figure S1). (A) INF-γ ELISPOT frequencies of T cells responding to tHIVconsvX peptide pools P1–P10 were measured in triplicates; the mean of the triplicates for each mouse was calculated. The number of animals responding to each pool is shown above bars. (B) The functionality and proportion of CD8^+^ and CD4^+^ T cells expressing multiple functions were assessed in an ICS assay using flow cytometry and performed in duplicate; the mean of the duplicates for each mouse was calculated, whereby 1 (black)—one function; 2 (light gray)—two functions; and 3 (dark gray)—three functions (see Figure S5 for the gating strategy). (C) Memory T cell subtypes were defined as T_CM_, central memory (CD44^hi^CD62L^hi^); T_EM_, effector memory (CD44^hi^CD62L^lo^); and T_N_, naive T cells (CD44^lo^CD62L^hi^) and all samples were analyzed in duplicate; the mean of the duplicates for each mouse was calculated. Graph data in (A) and (B) are shown as median ± IQR of the means, and pies in (B) and graphs in (C) show median of the means only (RR: n = 5 and RM: n = 7 mice).

Finally, we compared the performance of the RNA-RNA and RNA-MVA regimens in outbred animals. Groups of CD1-SWISS mice were primed with formulated AIR.tHIVconsv1 + AIR.tHIVconsv2 and boosted with the same or MVA.tHIVconsv3 + MVA.tHIVconsvsv4 using optimal timing for both regimens. Characterizing the vaccine-elicited responses in splenocytes using the IFN-γ ELISPOT assays, the RNA-RNA and RNA-MVA regimens induced total magnitudes (as sum of individual pool responses) of 1,868 and 5,411 SFUs/10^6^ splenocytes, respectively. For the RNA-RNA regimen, all 5 mice responded to 7 pools (P1, P3, P4, and P7–P10), only 1 mouse responded to 2 pools (P5 and P6), and 1 pool (P2) remained without any responder. For the RNA-MVA regimen, all pools had at least one responding animal, whereby all 7 mice responded to 5 pools (P1, P3, P6, P7, and P10) and varying numbers responded to the remaining pools: 1 (P2); 3 (P4); 4 (P5); 6 (P8); and 2 (P9; Figure 7A). Although the overall magnitudes may reflect the overall potency of the regimens, individual pool differences in the number of responding animals is likely attributed to the differences in individual animal haplotypes. All four functions IFN-γ, IL-2, TNF-α, and CD107a were detected in CD8^+^ T cells in blood and spleen, and CD4^+^ T cells lacked degranulation (Figure 7B). The overall functionality of tHIVconsvX-specific CD8^+^ T cell responses were similar between the two regimens (Figure 7B) and so were the T cell memory subtypes (Figure 7C). Thus, in outbred animals, the RNA-MVA regimen induced 2.9-fold higher frequency of HIV-1 conserved region-specific T cells relative to the RNA-RNA regimen, but the RNA-RNA responses were more plurifunctional.

## Discussion

In the present work, we characterized comprehensively the CD8^+^ and CD4^+^ T cell responses elicited in regimens involving formulated AIR saRNA-vectored subunit genetic vaccine candidates against HIV-1. In the BALB/c mice, we found that a single 5-μg saRNA dose of a bivalent-mosaic immunogen tHIVconsvX elicited over two thousand broadly specific T cells per million of splenocytes in total; these frequencies took at least 5 weeks to peak and were well maintained up to week 22 after vaccination. Furthermore, elicited T cells performed *in vitro* several effector functions upon peptide restimulation, killed *in vivo* epitope variants, and displayed structured memory subpopulations with well-represented effector and central memory. Homologous saRNA boost did not improve the induced T cell frequencies significantly, and no obvious effect of a time span between the prime and boost was readily discernible within the 2 and 6 weeks tested. In contrast, heterologous boosts with non-replicating virus vectors were able to at least double the HIV-1-specific T cell frequencies. The current superiority of the heterologous regimen over two saRNA deliveries was also demonstrated in a more rigorous model system of outbred animals. Thus, although a direct comparison of self-amplifying and non-self-amplifying mRNA showed 64-fold higher immunogenicity of the former,^52^ we see a great scope for all variations of this still very young platform to improve and shape future vaccine induction of both antibody and T cell responses.

We find the increase in T cell responses over 5 weeks and their long, sustained persistence intriguing. We are not aware of any studies suggesting, e.g., slow vaccine mRNA release from a depo, persistence of immunogenic protein, or requirement for any particular cell type uniquely explored by mRNA vaccines. We hypothesized that no immunogen is present for priming of T cell responses until after cell entry of saRNA and its translation. This would explain a delay of immune cell activation compared to protein- and/or live-virus-based treatments that already come with foreign and/or “danger” antigens. As saRNA translation begins, the immune responses can be generated with a steady trigger of ongoing protein expression up to the transfected cell death, which delivers another important trigger for immune response activation and releases immunogen for cross-presentation. Overall, we believe that these features generate a different immune response kinetic compared to other platforms. Further studies are warranted.

For HIV-1, there is a broad consensus on the importance of supporting development of vaccines aiming at induction of antibody-mediated protection. However, evidence of T-cell-mediated control of HIV-1 infection is ample. This comes from the temporal association of CD8^+^ T cell appearance and control of primary viremia,^53^, ^54^, ^55^, ^56^, ^57^, ^58^ extensive virus escape in targeted epitopes,^55^, ^59^, ^60^, ^61^ association of certain HLA class I alleles with HIV-1 control *in vivo*,^54^, ^59^, ^60^, ^62^, ^63^, ^64^ and definition of protective CD8^+^ T cell epitopes in patient cohorts.^21^, ^26^, ^65^, ^66^, ^67^ Also model infection of rhesus macaques with simian immunodeficiency virus supports importance of CD8^+^ T cells.^68^, ^69^, ^70^, ^71^, ^72^, ^73^, ^74^ Thus, inducing highly effective T cell responses by vaccination is likely to help broadly neutralizing antibodies reduce acquisition of HIV-1, as achieving truly sterilizing protection will be challenging and may be central to HIV cure. However, definition of beneficial qualities and quantity of T cells remain elusive. This is because HIV-1-specific T cells are a heterogeneous population, which, in natural HIV-1 infection, target both protective and non-protective viral epitopes.^26^, ^65^, ^66^ Thus, any attempts to associate a single CD8^+^ T cell property with HIV-1 control have to be carried out in the context of T cell specificity. Furthermore, HIV-1 control likely depends on a number of critical T cell traits required to be optimal at the same time, and if any one of these traits is suboptimal, the T cells, and therefore vaccines, may fail to protect.^9^, ^26^ These important parameters include specificity for protective epitopes,^26^, ^65^, ^66^ parallel recognition of multiple protective epitopes,^8^, ^75^, ^76^ optimal interaction with HLA-peptide complexes,^77^ rapid proliferation upon exposure to cognate peptides to reach protective numbers,^78^, ^79^ lysis of infected cells, and production of soluble antiviral and intercellular signaling molecules.^78^, ^79^, ^80^, ^81^ The CD8^+^ T cells induced by regimens involving saRNA displayed in the mouse model a number of the desired properties. Whether or not these will translate to humans and be beneficial remains to be seen, but the presented data encourage and deserve such translational studies. The BALB/c inbred mouse strain allowed detailed, sophisticated T cell analyses, and the CD1-SWISS mice demonstrated vaccine elicitation of desired responses in an outbred animal stock closer to a human population. However, many critical costimulatory signals, including stimulation through the toll-like receptors, differ between mice and humans, and therefore, having shown here the great potential of the saRNA technology to stimulate T cell responses, further optimization for human vaccines has to be carried out in humans. It follows that, to advance our understanding of T cell protection against HIV-1 infection, we need to measure multiple parameters in human vaccine studies.

Great promise of the mRNA vaccine platform comes from the ample possibilities to manipulate mRNA backbone, 5′ cap, UTRs, codon usage, nucleoside-base modifications, self-amplification, delivery systems, co-delivery of immunomodulatory molecules, safety, and manufacturing ease, which collectively provide enormous flexibility and room for optimization.^30^, ^35^, ^37^, ^42^, ^44^, ^82^ At this point of time, there are many more promises than human data and optimization of all these parameters in humans for each disease target may take some time.^42^ For example, it was demonstrated for self-amplifying mRNA that potent initial induction of type I interferons by double-stranded RNA (dsRNA) was counterproductive to immunogen expression and subsequent immunogenicity.^83^, ^84^ Thus, any rational exploitation of the innate signals and/or immunomodulation will require careful evaluation, which will ultimately need to be confirmed in the target species, i.e., humans.^85^ Nevertheless, new technologies can often precipitate the long-awaited breakthroughs in difficult challenges of public health, such as prevention and treatment of HIV-1, malaria, tuberculosis, cancer, and allergies, and the mRNA platform has the potential to be such a transforming technology. Many commercial programs have already started moving into clinic,^44^ and first results are emerging. We argue that the tHIVconsvX conserved regions and their match to global pandemic HIV-1s is currently state of the art and that the specificity of T cells and their breadth we aim to induce has a real potential to hurt HIV-1 where it is most vulnerable. This is strongly supported by our studies in ART treatment-naive, HIV-1-infected patients in Japan, whereby both the magnitude and breadth of tHIVconsvX-specific responses induced in natural HIV-1 infection correlated directly with high CD4 T cell count and indirectly with low plasma viral load.^26^, ^67^ saRNA is now poised to accelerate the iterative process of identifying and optimizing induction of the necessary protective T cell traits controlling HIV-1 infection in human clinical trials.

## Materials and Methods

### Preparation and Formulation of AIR.tHIVconsv1 and AIR.tHIVconsv2 saRNA Vaccines

The synthesis of the AIR.tHIVconsv1 and AIR.tHIVconsv2 saRNAs is based on T7 *in vitro* transcription protocols provided by MEGAscript T7 Transcription Kit (Thermo Fisher Scientific, Schwerte, Germany) described before.^83^ Briefly, a linear DNA template was generated containing the Semliki Forest virus isolate L10 saRNA vector (accession number AJ251359), and modifications were described elsewhere. The different tHIVconsvX sequences, including the co-transcriptional capping, with the synthetic cap analog γ-S-ARCA(D1). The *in vitro* transcription followed an already described process^86^ and was optimized with respect to saRNA length of up to 10,000 nt. For *in vivo* application, saRNA was formulated with a polyethylenimine-based reagent (Polyplus Tranfection, Illkirch, France). The formulation protocol followed to the supplier’s protocol and particle generation was confirmed by particle size measurement using a Dynamic Light Scattering instrument (Wyatt Technology, Dernbach, Germany). Only particles below 200 nm were released for *in vivo* application. All saRNA preparations were tested for their RNA integrity using a Bioanalyzer instrument internally optimized with respect to saRNA length (Agilent Technologies, Waldbronn, Germany). The preparation of other vaccines used in this study was described before.^26^, ^27^

### Mice and Immunizations and Preparation of Splenocytes

Six-week-old female BALB/c or CD1-SWISS mice were purchased from Envigo (UK) or Charles River Laboratories (UK), respectively, and housed at the Functional Genomics Facility, University of Oxford. Mice were immunized intramuscularly under general anesthesia either with varying amounts of saRNA as indicated in figures, a total 10^8^ infectious units (IUs) of rChAdOx1s, and a total of 5 × 10^6^ plaque-forming units (PFUs) of rMVAs. Immunization schedules are listed in Figure S1. On the day of sacrifice, spleens were collected and cells isolated by pressing organs individually through a 70-μm nylon mesh sterile cell strainer (Fisher Scientific) using a 5-mL syringe rubber plunger. Following the removal of red blood cells with RBC Lysing Buffer Hybri-Max (Sigma), splenocytes were washed and resuspended in R10 (RPMI 1640 supplemented with 10% fetal calf serum [FCS], penicillin and streptomycin, and β-mercaptoethanol) for ELISPOT, ICS assays, and other procedures. All procedures and care were approved by the local Clinical Medicine Ethical Review Committee, University of Oxford and conformed strictly to the United Kingdom Home Office Guidelines under the Animals (Scientific Procedures) Act 1986. Experiments were conducted under project license 30/3387 held by T.H.

### Peptides and Peptide Pools

All peptides were at least 90% pure by mass spectrometry (Ana Spec, San Jose, CA, USA and Synpeptide, Shanghai, China), dissolved in DMSO (Sigma-Aldrich) to yield a stock of 10 mg/mL, and stored at −80°C. Four hundred and one tHIVconsvX-derived peptides 15-mer overlapping by 11 amino acids were divided into 10 pools P1–P10 of 34–47 individual peptides in a way that variant peptides were always present in the same pool for use in ICS and ELISPOT assays. Also peptides pairs AMQ (AMQMLKETI and AMQMLKDTI) and VLV (VLVGPTPVNI and VLIGPTPVNI), a pool of 17 pairs of stimulatory “BALB/c” peptides, were employed as specified in each figure. The peptides were used at a final concentration of 2 μg/mL.

### The IFN-γ ELISPOT Assay

The ELISPOT assay was performed using the Mouse IFN-γ ELISpot kit (Mabtech) according to the manufacturer’s instructions. Immune splenocytes were collected and tested separately from individual mice. Peptides were used at 2 μg/mL each, and splenocytes at 2 × 10^5^ cells/well were added to 96-well high-protein-binding Immobilon-P membrane plates (Millipore) that had been precoated with 5 μg/mL anti-IFN-γ monoclonal antibody (mAb) AN18 (Mabtech, Stockholm, Sweden). The plates were incubated at 37°C in 5% CO_2_ for 18 hr and washed with PBS before the addition of 1 μg/mL biotinylated anti-IFN-γ Mab (Mabtech) at room temperature for 2 hr. The plates were then washed with PBS, incubated with 1 μg/mL streptavidin-conjugated alkaline phosphatase (Mabtech) at room temperature for 1 hr, washed with PBS, and individual cytokine-producing units were detected as dark spots after a 10-min reaction with 5-bromo-4-chloro-3-idolyl phosphate and nitro blue tetrazolium using an alkaline-phosphatase-conjugate substrate (Bio-Rad, Richmond, CA, USA). Spot-forming units were counted using the AID ELISpot Reader System (Autoimmun Diagnostika). The frequencies of responding cells were expressed as a number of spot-forming units/10^6^ splenocytes.

### ICS Assay

Splenocytes or PBMCs isolated from whole blood were stimulated with peptide at 2 μg/mL; ionomycin and phorbol myristate acetate (PMA) at 2.0 μg/mL and 0.5 μg/mL, respectively; or tissue culture media with 1% DMSO as a negative control. The cultures were supplemented with anti-CD107a phycoerythrin (PE)-conjugated mAb (eBioscience). The cells were incubated at 37°C, 5% CO_2_ for 2 hr prior to the addition of brefeldin A and monensin (BD Biosciences) and then left in culture overnight. The cells were centrifuged briefly, washed in PBS plus 5% BSA (Sigma-Aldrich), and the pellet resuspended in 40 μL of CD16/32 with LIVE/DEAD fixable aqua stain (Molecular Probes, Invitrogen). Cells were washed; a mastermix of anti-membrane marker mAbs was prepared containing CD4 allophycocyanin (APC)/Cy7 (BioLegend), CD3 PerCP-eFluor710, and CD8a eFluor 450 (both from eBioscience); and 40 μL added to each tube. The cells were incubated at 4°C for 30 min and then permeabilized using Fix/Perm solution (Becton Dickinson) for 20 min at 4°C. The cells were washed with Perm Wash buffer (Becton Dickinson), and a mastermix of anti-intracellular molecule mAbs was prepared containing IFN-γ PE-Cy7, IL-2 APC, and TNF-α fluorescein isothiocyanate (FITC) (all from eBioscience). The cells were incubated at 4°C for 30 min, washed, and resuspended in Perm Wash buffer prior to running on an LSRII flow cytometer (Becton Dickinson).

### Memory Subtype Assay

Splenocytes and PBMCs isolated from whole blood were stimulated with specific tHIVconsvX-derived BALB/c peptide pool and stained with 100 μL of a mastermix of anti-membrane marker mAbs containing LIVE/DEAD fixable aqua stain (Molecular Probes, Invitrogen), CD3-APC, CD4-FITC, CD8a-eFluor 450, CD44-PE, and CD62-L-PE-Cy7 605 (all from eBioscience). The cells were incubated at 4°C for 30 min, washed, and fixed with 1% paraformaldehyde in PBS prior to running on an LSRII flow cytometer (Becton Dickinson). The frequencies of the subtypes in CD8^+^ and CD4^+^ T cells represent the differences in stimulated and unstimulated immune cells.

### MHC Class I Tetramer Staining

Refolded MHC-peptide monomers (immunAware, Denmark) were tetramerized by adding streptavidin-conjugate-APC (Life Technologies) at 4°C. Splenocytes were stained with 30 μL of the optimal tetramer concentration for 20 min at room temperature and washed followed by the addition of 40 μL of a mastermix of anti-membrane marker mAbs containing LIVE/DEAD fixable aqua stain (Molecular Probes, Invitrogen), CD4 APC/Cy7 (BioLegend), CD3 PerCP-eFluor710, and CD8a eFluor 450 (both from eBioscience). The cells were incubated at 4°C for 30 min, washed, and fixed with 1% paraformaldehyde in PBS prior to running on an LSRII flow cytometer (Becton Dickinson).

### *In Vivo* Killing Assay

Syngeneic splenocytes were incubated with or without 2 μg/mL peptides at 37°C, 5% CO_2_ for 90 min and thoroughly washed. Unpulsed cells were labeled with 5-(and-6)-([(4-chloromethyl)benzoyl]amino)tetramethylrhodamine (CMTMR; Molecular Probes, Invitrogen) only, and peptide-pulsed cells were labeled with 750 nM (AMQMLKETI - 1 and VLVGPTPVNI - 3) and 150 nM (AMQMLKDTI - 2 and ILVGPTPVNI - 4) 5(6)-carboxyfluorescein diacetate N-succinimidyl ester (CFSE) (Molecular Probes; Invitrogen); in addition, the VLVGPTPVNI and ILVGPTPVNI peptide-pulsed cells were incubated with 10 μM CMTMR at 37°C for 15 min and a further 15 min in fresh medium. Five differentially labeled cell cultures were combined for intravenous adoptive transfer 5 weeks after saRNA and 1 week after rMVA immunizations, with each animal receiving approximately 2 × 10^6^ cells of each population. Ten hours later, splenocytes were isolated and analyzed using flow cytometry. Cytolytic activity was estimated using the following formula: adjusted % survival = 100 × (% survival of peptide-pulsed cells/mean % survival of peptide unpulsed cells), followed by the calculation of % specific lysis = 100 − adjusted % survival.

### CSFE Proliferation Assay

Cryopreserved splenocytes were thawed, resuspended in pre-warmed PBS with 0.1% BSA at a final concentration of 1 × 10^6^ cells/mL, and labeled with 750 nM CFSE for 10 min at 37°C, 5% CO_2_. The staining was quenched by adding 5 volumes of ice-cold R10 followed by a 5-min incubation on ice. The cells were pelleted, washed, and plated in 96-well round-bottom plates at a concentration of 1 × 10^6^ cells/well. The CFSE-labeled cells were then stimulated for 5 days with 2 μg/mL of peptide, 2.0 μg/mL ionomycin, and 0.5 μg/mL PMA (positive control) or tissue culture media with 1% DMSO (negative control). The cells were stained with a mastermix containing the dead cell marker (LIVE/DEAD Fixable Aqua stain; Invitrogen) and anti-membrane marker mAbs anti-CD4-APC/Cy7 (BioLegend), anti-CD3-PerCPeFluor710, and anti-CD8-eFluor450 (both from eBioscience), fixed and acquired on a BD LSR II flow cytometer. Data analysis was performed using FlowJo software (Tree Star) with gating shown in Figure S6.

### Statistical Analysis

Statistical analyses were performed using Graph Pad Prism version 7. ELISPOT and flow cytometry results were assumed to be non-Gaussian in distribution; thus, non-parametric tests were used throughout and medians (range) are shown. Multiple comparisons were performed using the Kruskal-Wallis test with Dunn’s multiple comparison post test for nonparametric data. Groups with the same *in vitro* restimulations were compared using two-tailed Mann-Whitney U tests. Two-tailed p values were used, and p values of less than 0.05 were considered statistically significant.

## Author Contributions

N.M. designed and conducted the experiments and analyzed the data, A.B.V. planned and prepared the saRNA vaccines, S.B. predicted BALB/c epitopes and prepared tetramers, S.E. prepared the saRNA vaccines, U.S. supervised the study and experimental strategy, and T.H. designed the experiments, analyzed data, and wrote the manuscript with contribution from all authors.

## Conflicts of Interest

T.H. is one of the inventors on PCT Application No. PCT/US2014/058422 and EP14846993.5 concerning the tHIVconsvX immunogen. A.B.V., S.E., and U.S. are inventors on patents and/or patent applications that cover parts of this article. U.S. is co-founder and CEO of BioNTech AG (Mainz, Germany), a privately held biopharmaceutical company developing RNA-based vaccines and immunotherapeutics. A.B.V. and S.E. are employees of BioNTech RNA Pharmaceuticals GmbH. The other authors declare no conflicts of interest.

## Supplemental Information

Document S1. Figures S1–S8

Document S2. Article plus Supplemental Information
