# Supplementary material for: Efficient Induction of T Cells against Conserved HIV-1 Regions by Mosaic Vaccines Delivered as Self-Amplifying mRNA
Source: Mol Ther Methods Clin Dev. 2018 Oct 26;12:32–46. doi: 10.1016/j.omtm.2018.10.010 (PMC6258890; doi:10.1016/j.omtm.2018.10.010)
Supplement: Document S1. Figures S1–S8 [file mmc1.pdf]

OMTM, Volume *12*

Supplemental Information

Efficient Induction of T Cells against Conserved HIV-1 Regions by Mosaic Vaccines Delivered as Self-Amplifying mRNA

Nathifa Moyo, Annette B. Vogel, Søren Buus, Stephanie Erbar, Edmund G. Wee, Ugur Sahin, and Tomáš Hanke

Figure 2**a** – Dose response in BALB/c mice

Group	n	Regimen	Dose	Week 0	Week 5
1	3	R1	5 µg	AIR.tHIVconsv1	Cull
2	3	R1	1 µg	AIR.tHIVconsv1	Cull
3	3	R1	200 ng	AIR.tHIVconsv1	Cull
4	3	R1	40 ng	AIR.tHIVconsv1	Cull
5	3	R2	5 µg	AIR.tHIVconsv2	Cull
6	3	R2	1 µg	AIR.tHIVconsv2	Cull
7	3	R2	200 ng	AIR.tHIVconsv2	Cull
8	3	R2	40 ng	AIR.tHIVconsv2	Cull
9	3	R	2.5 µg + 2.5 µg	AIR.tHIVconsv1 + AIR.tHIVconsv2	Cull
10	3	R	0.5 µg + 0.5 µg	AIR.tHIVconsv1 + AIR.tHIVconsv2	Cull
11	3	R	100 ng + 100 ng	AIR.tHIVconsv1 + AIR.tHIVconsv2	Cull
12	3	R	20 ng + 20 ng	AIR.tHIVconsv1 + AIR.tHIVconsv2	Cull

Figure 2**b** – Adjuvantation in BALB/c mice

Groups	n	Regimen	Week 0	Week 2 or Week 5
1 & 4	2x3	R	AIR.tHIVconsv1 + AIR.tHIVconsv2 Polymer	Cull
2 & 5	2x3	R	AIR.tHIVconsv1 + AIR.tHIVconsv2	Cull
3 & 6	2x3	-	Polymer	Cull

Figure 3a and **c** – Kinetics of responses up to 5 weeks in BALB/c mice

Groups	n	Regimen	Week 0	Week 1 – Week 5 Weekly
1 - 5	5x5	R1	AIR.tHIVconsv1	Cull
6 - 10	5x5	R2	AIR.tHIVconsv2	Cull
11 - 15	5x5	R	AIR.tHIVconsv1 + AIR.tHIVconsv2	Cull

Figure 3**b** and 4 - Extended kinetics of responses up to 22 weeks in BALB/c mice

Groups	n	Regimen	Week 0	Week 6 - Week 22 Biweekly
1 - 9	9x5	R	AIR.tHIVconsv1 + AIR.tHIVconsv2	Cull

Figure 5 - Optimizing the time of homologous boost in BALB/c mice

Groups	n	Reg[a]	Week 0	Week 2	Week 4	Week 6	Week 10/14/18
1 & 4	3x5	R2R	-	-	AIR.tHIVconsv1 + AIR.tHIVconsv2	AIR.tHIVconsv1 + AIR.tHIVconsv2	Cull
2 & 5	3x5	R4R	-	AIR.tHIVconsv1 + AIR.tHIVconsv2	-	AIR.tHIVconsv1 + AIR.tHIVconsv2	Cull
3 & 6	3x5	R6R	AIR.tHIVconsv1 + AIR.tHIVconsv2	-	-	AIR.tHIVconsv1 + AIR.tHIVconsv2	Cull

Figure 6**a** Left & Middle – Heterologous regimens in BALB/c mice

Group	n	Reg	Week 0	Week 1	Week 2	Week 5	Week 6	Week 7
1	4	R	-	-	R	-	-	Cull
2	4	C	-	-	-	C	-	Cull
3	4	M	-	-	-	-	M	Cull
4	4	RR	R	-	R	-	-	Cull
5	4	RM	-	R	-	-	M	Cull
6	4	RC	R	-	-	C	-	Cull

Figure 6**a** Right– Heterologous regimens in BALB/c mice

Group	n	Reg	Week 0	Week 4	Week 6	Week 7
1	5	R	-	AIR.tHIVconsv1 + AIR.tHIVconsv2	-	Cull
2	5	RR	AIR.tHIVconsv1 + AIR.tHIVconsv2	AIR.tHIVconsv1 + AIR.tHIVconsv2	-	Cull
3	5	RM	-	AIR.tHIVconsv1 + AIR.tHIVconsv2	MVA.tHIVconsv3 + MVA.tHIVconsv4	Cull
4	5	RC	-	AIR.tHIVconsv1 + AIR.tHIVconsv2	ChAdOx1.tHIVconsv5 + ChAdOx1.tHIVconsv6	Cull

Figure 6**b** – $CD8^+$ T-cell peptide mapping in BALB/c mice

Group	n	Reg	Week 0	Week 4	Week 5
1	5	RM	AIR.tHIVconsv1 + AIR.tHIVconsv2	MVA.tHIVconsv3 + MVA.tHIVconsv4	Cull

Figure 6**c** and **d** – $CD8^+$ T-cell peptide mapping in BALB/c mice

Group	n	Reg	Week 0	Week 4	Week 5
1	4	RM	AIR.tHIVconsv1 + AIR.tHIVconsv2	MVA.tHIVconsv3 + MVA.tHIVconsv4	Cull

Figure 6**e** – In vivo killing in BALB/c mice

Group	n	Reg	Week 0	Week 2	Week 4	Week 5
1	3	R	AIR.tHIVconsv1 + AIR.tHIVconsv2	-	-	Cull
2	3	RM	-	AIR.tHIVconsv1 + AIR.tHIVconsv2	MVA.tHIVconsv3 + MVA.tHIVconsv4	Cull

Figure 7 Heterologous regimens in outbred CD1-SWISS mice

Group	n	Reg	Week 0	Week 4	Week 5	Week 7	Week 8
1	5[b]	RR	AIR.tHIVconsv1 + AIR.tHIVconsv2	AIR.tHIVconsv1 + AIR.tHIVconsv2	-	-	Cull
2	7	RM	-	-	AIR.tHIVconsv1 + AIR.tHIVconsv2	MVA.tHIVconsv3 + MVA.tHIVconsv4	Cull

Figure S1 Immunization regimens. Mice were immunized using the indicated regimens. All vaccines were delivered intramuscularly. R - 2.5 µg of AIR.tHIVconsv1 RNA + 2.5 µg of AIR.tHIVconsv2 RNA formulated; M – 2.5×10^6 PFU of MVA.tHIVconsv3 + 2.5×10^6 PFU of MVA.tHIVconsv4; C - 5×10^7 IU of ChAdOx1.tHIVconsv5 + 5×10^7 IU of ChAdOx1.tHIVconsv6. a – In R2R, R4R and R6R, the number indicates the gap in weeks between prime and boost administrations. b - 2 out of 7 mice died of vaccine-unrelated causes.

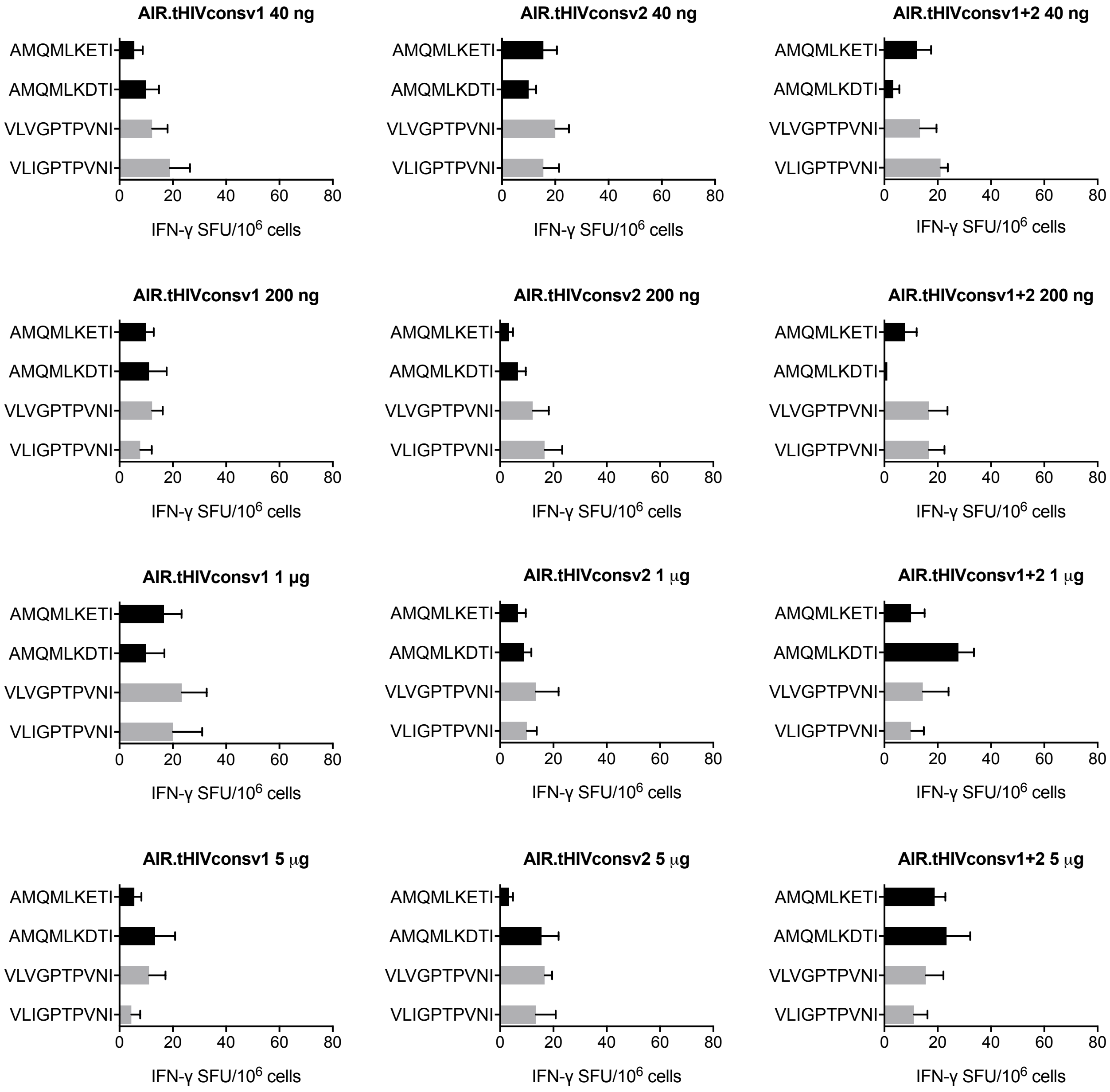

Figure S2 Dose response to saRNA vaccine at 1 week after vaccination. Groups of BALB/c mice were immunized with increasing doses of saRNA vaccine as indicated above the graphs, sacrificed 1 week later and their splenocytes were assessed in an IFN-γ ELISPOT assay using the two variants of the two most immunodominant epitopes for the vaccine-elicited frequencies of specific $CD8^+$ T cells. Data are shown as mean ± SD (n = 3).

**Paired peptide IDs**	**Variant IDs**	**Sequences**	
1	C001	PIVQNLQGQMVHQAI	**POOL 1**
	C002	PIVQNAQGQMVHQAL	**44 peptides**
2	C003	NLQGQMVHQAISPRT	
	C004	NAQGQMVHQALSPRT	
3	C005	QMVHQAISPRTLNAW	
	C006	QMVHQALSPRTLNAW	
4	C007	QAISPRTLNAWVKVI	
	C008	QALSPRTLNAWVKVV	
5	C009	PRTLNAWVKVIEEKA	
	C010	PRTLNAWVKVVEEKA	
6	C011	NAWVKVIEEKAFSPE	
	C012	NAWVKVVEEKAFSPE	
7	C013	KVIEEKAFSPEVIPM	
	C014	KVVEEKAFSPEVIPM	
8	C015	EKAFSPEVIPMFTAL	
	C016	EKAFSPEVIPMFSAL	
9	C017	SPEVIPMFTALSEGA	
	C018	SPEVIPMFSALSEGA	
10	C019	IPMFTALSEGATPQD	
	C020	IPMFSALSEGATPQD	
11	C021	TALSEGATPQDLNTM	
	C022	SALSEGATPQDLNMM	
12	C023	EGATPQDLNTMLNTV	
	C024	EGATPQDLNMMLNIV	
13	C025	PQDLNTMLNTVGGHQ	
	C026	PQDLNMMLNIVGGHQ	
14	C027	NTMLNTVGGHQAAMQ	
	C028	NMMLNIVGGHQAAMQ	
15	C029	NTVGGHQAAMQMLKD	
	C030	NIVGGHQAAMQMLKE	
16	C031	GHQAAMQMLKDTINE	
	C032	GHQAAMQMLKETINE	
17	C033	AMQMLKDTINEEAAE	
	C034	AMQMLKETINEEAAE	
18	C035	LKDTINEEAAEWDRV	
	C036	LKETINEEAAEWDRL	
19	C037	INEEAAEWDRVHPVH	
	C038	INEEAAEWDRLHPVH	
20	C039	AAEWDRVHPVHAGPI	
	C040	AAEWDRLHPVHAGPI	
21	C041	DRVHPVHAGPIAPGQ	
	C042	DRLHPVHAGPIPPGQ	
22	C043	PVHAGPIAPGQMREP	
	C044	PVHAGPIPPGQMREP	
23	C045	GPIAPGQMREPRGSD	**POOL 2**
	C046	GPIPPGQMREPRGSD	**47 peptides**
24	C047	PGQMREPRGSDIAGT	
25	C048	REPRGSDIAGTTSNL	

	C049	REPRGSDIAGTTSTL	
26	C050	GSDIAGTTSNLQEQI	
	C051	GSDIAGTTSTLQEQI	
27	C052	AGTTSNLQEQIGWMT	
	C053	AGTTSTLQEQIGWMT	
28	C054	SNLQEQIGWMTSNPP	
	C055	STLQEQIGWMTNNPP	
29	C056	EQIGWMTSNPPIPVG	
	C057	EQIGWMTNNPPIPVG	
30	C058	WMTSNPPIPVGDIYK	
	C059	WMTNNPPIPVGEIYK	
31	C060	NPPIPVGDIYKRWII	
	C061	NPPIPVGEIYKRWII	
32	C062	PVGDIYKRWIILGLN	
	C063	PVGEIYKRWIIMGLN	
33	C064	IYKRWIILGLNKIVR	
	C065	IYKRWIIMGLNKIVR	
34	C066	WIILGLNKIVRMYSP	
	C067	WIIMGLNKIVRMYSP	
35	C068	GLNKIVRMYSPVSIL	
	C069	GLNKIVRMYSPTSIL	
36	C070	IVRMYSPVSILDIRQ	
	C071	IVRMYSPTSILDIKQ	
37	C072	YSPVSILDIRQGPKE	
	C073	YSPTSILDIKQGPKE	
38	C074	SILDIRQGPKEPFRD	
	C075	SILDIKQGPKEPFRD	
39	C076	IRQGPKEPFRDYVDR	
	C077	IKQGPKEPFRDYVDR	
40	C078	PKEPFRDYVDRFFKT	
	C079	PKEPFRDYVDRFYKT	
41	C080	FRDYVDRFFKTLRAE	
	C081	FRDYVDRFYKTLRAE	
42	C082	VDRFFKTLRAEQATQ	
	C083	VDRFYKTLRAEQASQ	
43	C084	FKTLRAEQATQEVKN	
	C085	YKTLRAEQASQEVKN	
44	C086	RAEQATQEVKNWMTD	
	C087	RAEQASQEVKNWMTE	
45	C088	ATQEVKNWMTDTLLV	
	C089	ASQEVKNWMTETLLV	
46	C090	VKNWMTDTLLVQNAN	
	C091	VKNWMTETLLVQNAN	
47	C092	MTDTLLVQNANPDCK	**POOL 3**
	C093	MTETLLVQNANPDCK	**47 peptides**
48	C094	LLVQNANPDCKTILR	
	C095	LLVQNANPDCKTILK	
49	C096	NANPDCKTILRALGP	
	C097	NANPDCKTILKALGP	
50	C098	DCKTILRALGPGATL	
	C099	DCKTILKALGPAATL	
51	C100	ILRALGPGATLEEMM	
	C101	ILKALGPAATLEEMM	

52	C102	LGPGATLEEMMTACQ	
	C103	LGPAATLEEMMTACQ	
53	C104	ATLEEMMTACQGVGG	
54	C105	EMMTACQGVGGPGHK	
	C106	EMMTACQGVGGPSHK	
55	C107	ACQGVGGPGHKARVL	
	C108	ACQGVGGPSHKARVL	
56	C109	KCFNCGKEGHIAKNC	
	C110	KCFNCGKEGHLARNC	
57	C111	CGKEGHIAKNCRAPR	
	C112	CGKEGHLARNCRAPR	
58	C113	GHIAKNCRAPRKRGC	
	C114	GHLARNCRAPRKKGC	
59	C115	KNCRAPRKRGCWKCG	
	C116	RNCRAPRKKGCWKCG	
60	C117	APRKRGCWKCGREGH	
	C118	APRKKGCWKCGKEGH	
61	C119	RGCWKCGREGHQMKD	
	C120	KGCWKCGKEGHQMKD	
62	C121	KCGREGHQMKDCNER	
	C122	KCGKEGHQMKDCTER	
63	C123	EGHQMKDCNERQANF	
	C124	EGHQMKDCTERQANF	
64	C125	MKDCNERQANFLGKI	
	C126	MKDCTERQANFLGKI	
65	C127	NERQANFLGKIWPSH	
	C128	TERQANFLGKIWPSN	
66	C129	ANFLGKIWPSHKGRP	
	C130	ANFLGKIWPSNKGRP	
67	C131	GKIWPSHKGRPGNFL	
	C132	GKIWPSNKGRPGNFP	
68	C133	PSHKGRPGNFLQSRP	
	C134	PSNKGRPGNFPQSRP	
69	C135	GRPGNFLQSRPEPTA	**135+137**
	C137	GRPGNFPQSRPEPSA	
70	C136	NFLQSRPEPTAPP	**136+138**
	C138	NFPQSRPEPSAPP	
71	C139	LPGKWKPKMIGGIGG	**POOL 4**
	C140	LPGRWKPKMIGGIGG	**41 peptides**
72	C141	WKPKMIGGIGGFIKV	
73	C142	MIGGIGGFIKVKQYD	
	C143	MIGGIGGFIKVRQYD	
74	C144	IGGFIKVKQYDQILI	
	C145	IGGFIKVRQYDQIPI	
75	C146	IKVKQYDQILIEICG	
	C147	IKVRQYDQIPIEICG	
76	C148	QYDQILIEICGKKAI	
	C149	QYDQIPIEICGHKAI	
77	C150	ILIEICGKKAIGTVL	
	C151	IPIEICGHKAIGTVL	
78	C152	ICGKKAIGTVLVGPT	
	C153	ICGHKAIGTVLIGPT	
79	C154	KAIGTVLVGPTPVNI	

	C155	KAIGTVLIGPTPVNI	
80	C156	TVLVGPTPVNIIGRN	
	C157	TVLIGPTPVNIIGRN	
81	C158	GPTPVNIIGRNMLTQ	
	C159	GPTPVNIIGRNLLTQ	
82	C160	VNIIGRNMLTQLGCT	
	C161	VNIIGRNLLTQIGCT	
83	C162	GRNMLTQLGCTLNFP	
	C163	GRNLLTQIGCTLNFP	
84	C164	LTQLGCTLNFPISPI	
	C165	LTQIGCTLNFPISPI	
85	C166	GCTLNFPISPIDTVP	
	C167	GCTLNFPISPIETVP	
86	C168	NFPISPIDTVPVTLK	
	C169	NFPISPIETVPVKLK	
87	C170	SPIDTVPVTLKPGMD	
	C171	SPIETVPVKLKPGMD	
88	C172	TVPVTLKPGMDGPRV	
	C173	TVPVKLKPGMDGPKV	
89	C174	TLKPGMDGPRVKQWP	
	C175	KLKPGMDGPKVKQWP	
90	C176	GMDGPRVKQWPLTEE	
	C177	GMDGPKVKQWPLTEE	
91	C178	PRVKQWPLTEEKIKA	
	C179	PKVKQWPLTEEKIKA	
92	C180	QWPLTEEKIKALTEI	**POOL 5**
	C181	QWPLTEEKIKALVEI	**40 peptides**
93	C182	TEEKIKALTEICKEM	
	C183	TEEKIKALVEICTEM	
94	C184	IKALTEICKEMEKEG	
	C185	IKALVEICTEMEKEG	
95	C186	TEICKEMEKEGKITK	
	C187	VEICTEMEKEGKISK	
96	C188	KEMEKEGKITKIGPE	
	C189	TEMEKEGKISKIGPE	
97	C190	KEGKITKIGPENPYN	
	C191	KEGKISKIGPENPYN	
98	C192	ITKIGPENPYNTPIF	
	C193	ISKIGPENPYNTPVF	
99	C194	GPENPYNTPIFAIKK	
	C195	GPENPYNTPVFAIKK	
100	C196	PYNTPIFAIKKKDST	
	C197	PYNTPVFAIKKKDST	
101	C198	PIFAIKKKDSTKWRK	
	C199	PVFAIKKKDSTRWRK	
102	C200	IKKKDSTKWRKLVDF	
	C201	IKKKDSTRWRKLVDF	
103	C202	DSTKWRKLVDFRELN	
	C203	DSTRWRKLVDFRELN	
104	C204	WRKLVDFRELNKKTQ	
	C205	WRKLVDFRELNKRTQ	
105	C206	VDFRELNKKTQDFWE	
	C207	VDFRELNKRTQDFWE	

106	C208	ELNKKTQDFWEVQLG	
	C209	ELNKRTQDFWEVQLG	
107	C210	KTQDFWEVQLGIPHP	
	C211	RTQDFWEVQLGIPHP	
108	C212	FWEVQLGIPHPAGLK	
	C213	FWEVQLGIPHPSGLK	
109	C214	QLGIPHPAGLKKKKS	
	C215	QLGIPHPSGLKKKRS	
110	C216	PHPAGLKKKKSVTVL	
	C217	PHPSGLKKKRSVTVL	
111	C218	GLKKKKSVTVLDVGD	
	C219	GLKKKRSVTVLDVGD	
112	C220	KKSVTVLDVGDAYFS	**POOL 6**
	C221	KRSVTVLDVGDAYFS	**39 peptides**
113	C222	TVLDVGDAYFSVPLD	
114	C224	VGDAYFSVPLDESFR	
	C225	VGDAYFSVPLDKDFR	
115	C226	YFSVPLDESFRKYTA	
	C227	YFSVPLDKDFRKYTA	
116	C228	PLDESFRKYTAFTIP	
	C229	PLDKDFRKYTAFTIP	
117	C230	SFRKYTAFTIPSINN	
	C231	DFRKYTAFTIPSTNN	
118	C232	YTAFTIPSINNETPG	
	C233	YTAFTIPSTNNETPG	
119	C234	TIPSINNETPGIRYQ	
	C235	TIPSTNNETPGVRYQ	
120	C236	INNETPGIRYQYNVL	
	C237	TNNETPGVRYQYNVL	
121	C238	TPGIRYQYNVLPQGW	
	C239	TPGVRYQYNVLPMGW	
122	C240	RYQYNVLPQGWKGSP	
	C241	RYQYNVLPMGWKGSP	
123	C242	NVLPQGWKGSPAIFQ	
	C243	NVLPMGWKGSPAIFQ	
124	C244	QGWKGSPAIFQSSMT	
	C245	MGWKGSPAIFQCSMT	
125	C246	GSPAIFQSSMTKILE	
	C247	GSPAIFQCSMTKILE	
126	C248	IFQSSMTKILEPFRA	
	C249	IFQCSMTKILEPFRK	
127	C250	SMTKILEPFRAKNPE	
	C251	SMTKILEPFRKQNPD	
128	C252	ILEPFRAKNPEIVIY	
	C253	ILEPFRKQNPDIVIY	
129	C254	FRAKNPEIVIYQYMD	
	C255	FRKQNPDIVIYQYMD	
130	C256	NPEIVIYQYMDDLYV	
	C257	NPDIVIYQYMDDLYI	
131	C258	VIYQYMDDLYVGSDL	
	C259	VIYQYMDDLYIGSDL	
132	C260	YMDDLYVGSDLEIGQ	
	C261	YMDDLYIGSDLEIGQ	

133	C262	LYVGSDLEIGQHRAK	**POOL 7**
	C263	LYIGSDLEIGQHRTK	**39 peptides**
134	C264	SDLEIGQHRAKIEEL	
	C265	SDLEIGQHRTKIEEL	
135	C266	IGQHRAKIEELREHL	
	C267	IGQHRTKIEELRQHL	
136	C268	RAKIEELREHLLKWG	
	C269	RTKIEELRQHLLRWG	
137	C270	EELREHLLKWGFTTP	
	C271	EELRQHLLRWGFTTP	
138	C272	EHLLKWGFTTPDKKH	
	C273	QHLLRWGFTTPDKKH	
139	C274	KWGFTTPDKKHQKEP	
	C275	RWGFTTPDKKHQKEP	
140	C276	TTPDKKHQKEPPFLW	
	C277	TTPDKKHQKEPPFHW	
141	C278	KKHQKEPPFLWMGYE	
	C279	KKHQKEPPFHWMGYE	
142	C280	KEPPFLWMGYELHPD	
	C281	KEPPFHWMGYELHPD	
143	C282	FLWMGYELHPDRWTV	
	C283	FHWMGYELHPDKWTV	
144	C284	GYELHPDRWTVQPIQ	
	C285	GYELHPDKWTVQPIV	
145	C286	HPDRWTVQPIQLPEK	
	C287	HPDKWTVQPIVLPEK	
146	C288	WTVQPIQLPEKESWT	
	C289	WTVQPIVLPEKDSWT	
147	C290	PIQLPEKESWTVNDI	
	C291	PIVLPEKDSWTVNDI	
148	C292	PEKESWTVNDIQKLI	
	C293	PEKDSWTVNDIQKLV	
149	C294	SWTVNDIQKLIGKLN	
	C295	SWTVNDIQKLVGKLN	
150	C296	NDIQKLIGKLNWASQ	
	C297	NDIQKLVGKLNWASQ	
151	C298	KLIGKLNWASQIY	
	C299	KLVGKLNWASQIY	
152	C300	AEIQKQGQDQWTYQI	**POOL 8**
	C301	AEIQKQGQGQWTYQI	**42 peptides**
153	C302	KQGQDQWTYQIYQEP	
	C303	KQGQGQWTYQIYQEP	
154	C304	DQWTYQIYQEPFKNL	
	C305	GQWTYQIYQEPYKNL	
155	C306	YQIYQEPFKNLKTGK	
	C307	YQIYQEPYKNLKTGK	
156	C308	QEPFKNLKTGKYA	
	C309	QEPYKNLKTGKYA	
157	C310	FNLPPIVAKEIVANC	
	C311	FNLPPVVAKEIVASC	
158	C312	PIVAKEIVANCDKCQ	
	C313	PVVAKEIVASCDKCQ	
159	C314	KEIVANCDKCQLKGE	

	C315	KEIVASCDKCQLKGE	
160	C316	ANCDKCQLKGEAMHG	
	C317	ASCDKCQLKGEAIHG	
161	C318	KCQLKGEAMHGQVDC	
	C319	KCQLKGEAIHGQVDC	
162	C320	KGEAMHGQVDCSPGI	
	C321	KGEAIHGQVDCSPGI	
163	C322	MHGQVDCSPGIWQLD	
	C323	IHGQVDCSPGMWQLD	
164	C324	VDCSPGIWQLDCTHL	
	C325	VDCSPGMWQLDCTHL	
165	C326	PGIWQLDCTHLEGKV	
	C327	PGMWQLDCTHLEGKV	
166	C328	QLDCTHLEGKVILVA	
	C329	QLDCTHLEGKIILVA	
167	C330	THLEGKVILVAVHVA	
	C331	THLEGKIILVAVHVA	
168	C332	GKVILVAVHVASGY	
	C333	GKIILVAVHVASGY	
169	C334	QEFGIPYNPQSQGVV	**POOL 9**
170	C335	IPYNPQSQGVVESMN	**34 peptides**
171	C336	PQSQGVVESMNKELK	
	C337	PQSQGVVESMNNELK	
172	C338	GVVESMNKELKKIIG	
	C339	GVVESMNNELKKIIG	
173	C340	SMNKELKKIIGQVRE	
	C341	SMNNELKKIIGQVRD	
174	C342	ELKKIIGQVREQAEH	
	C343	ELKKIIGQVRDQAEH	
175	C344	IIGQVREQAEHLKTA	
	C345	IIGQVRDQAEHLKTA	
176	C346	VREQAEHLKTAVQMA	
	C347	VRDQAEHLKTAVQMA	
177	C348	AEHLKTAVQMAVFIH	
	C349	AEHLKTAVQMAVLIH	
178	C350	KTAVQMAVFIHNFKR	
	C351	KTAVQMAVLIHNFKR	
179	C352	QMAVFIHNFKRKGGI	
	C353	QMAVLIHNFKRRGGI	
180	C354	FIHNFKRKGGIGGYS	
	C355	LIHNFKRRGGIGGYS	
181	C356	FKRKGGIGGYSAGER	
	C357	FKRRGGIGGYSAGER	
182	C358	GGIGGYSAGERIIDI	
	C359	GGIGGYSAGERIVDI	
183	C360	GYSAGERIIDIIATD	
	C361	GYSAGERIVDIIATD	
184	C362	GERIIDIIATDIQTK	
	C363	GERIVDIIATDIQTR	
185	C364	IDIIATDIQTKELQK	
	C365	VDIIATDIQTRELQK	
186	C366	ATDIQTKELQKQITK	
	C367	ATDIQTRELQKQIIK	

187	C368	QTKELQKQITKIQNF	**POOL 10**
	C369	QTRELQKQIIKIQNF	**34 peptides**
188	C370	LQKQITKIQNFRVYY	
	C371	LQKQIIKIQNFRVYY	
189	C372	ITKIQNFRVYYRDSR	
	C373	IIKIQNFRVYYRDSR	
190	C374	QNFRVYYRDSRDPIW	
	C375	QNFRVYYRDSRDPLW	
191	C376	VYYRDSRDPIWKGPA	
	C377	VYYRDSRDPLWKGPA	
192	C378	DSRDPIWKGPAKLLW	
	C379	DSRDPLWKGPAKLLW	
193	C380	PIWKGPAKLLWKGEG	
	C381	PLWKGPAKLLWRGEG	
194	C382	GPAKLLWKGEGAVVI	
	C383	GPAKLLWRGEGAVVI	
195	C384	LLWKGEGAVVIQDNS	
	C385	LLWRGEGAVVIQDNS	
196	C386	GEGAVVIQDNSDIKV	
	C387	GEGAVVIQDNSEIKV	
197	C388	VVIQDNSDIKVVPRR	
	C389	VVIQDNSEIKVVPRR	
198	C390	DNSDIKVVPRRKVKI	
	C391	DNSEIKVVPRRKAKI	
199	C392	IKVVPRRKVKIIKDY	
	C393	IKVVPRRKAKIIRDY	
200	C394	PRRKVKIIKDYGKQM	
	C395	PRRKAKIIRDYGKQM	
201	C396	VKIIKDYGKQMAGAD	
	C397	AKIIRDYGKQMAGDD	
202	C398	KDYGKQMAGADCVAG	
	C399	RDYGKQMAGDDCVAS	
203	C400	KQMAGADCVAGRQDED	
	C401	KQMAGDDCVASRQDED	

Figure S3 The tHIVconsvX-derived 15-mer peptides and their pairing and pooling. The tHIVconsvX immunogens contain 2 Gag and 4 Pol conserved regions and are designed as a bi-valent mosaic. This means that with a few exceptions, each of the selected 15-amino acid windows has two versions, which differ in about 10% of amino acids. The altogether 401 overlapping 15-mer peptides are organized into 10 pools such, that each related peptide pair is present in the same pool. This way, individual pool frequencies can be added to estimate the overall magnitude of the T-cell response to the tHIVconsvX conserved regions without counting the same responses twice. Peptides highlighted blue have no variant pair; these are C047, C104, C141, C222, C334, C335. Peptides highlighted yellow do not follow in sequential order of its variant.

C031	GHQAAMQMLKDTINE
C032	GHQAAMQMLKETINE
C033	AMQMLKDTINEEAAE
C034	AMQMLKETINEEAAE
C062	PVGDIYKRWIILGLN
C063	PVGEIYKRWIIMGLN
C082	VDRFFKTLRAEQATQ
C083	VDRFYKTLRAEQASQ
C154	KAIGTVLVGPTPVNI
C155	KAIGTVLIGPTPVNI
C156	TVLVGPTPVNIIGRN
C157	TVLIGPTPVNIIGRN
C180	QWPLTEEKIKALTEI
C181	QWPLTEEKIKALVEI
C246	GSPAIFQSSMTKILE
C247	GSPAIFQCSMTKILE
C248	IFQSSMTKILEPFRA
C249	IFQCSMTKILEPFRK
C262	LYVGSDLEIGQHRAK
C263	LYIGSDLEIGQHRTK
C270	EELREHLLKWGFTTP
C271	EELRQHLLRWGFTTP
C272	EHLLKWGFTTPDKKH
C273	QHLLRWGFTTPDKKH
C350	KTAVQMAVFIHNFKR
C351	KTAVQMAVLIHNFKR
C352	QMAVFIHNFKRKGGI
C353	QMAVLIHNFKRRGGI
C372	ITKIQNFRVYYRDSR
C373	IIKIQNFRVYYRDSR
C374	QNFRVYYRDSRDPIW
C375	QNFRVYYRDSRDPLW
C376	VYYRDSRDPIWKGPA
C377	VYYRDSRDPLWKGPA

Figure S4 The BALB/c pool of peptides recognized by T cells induced by a single dose saRNA.

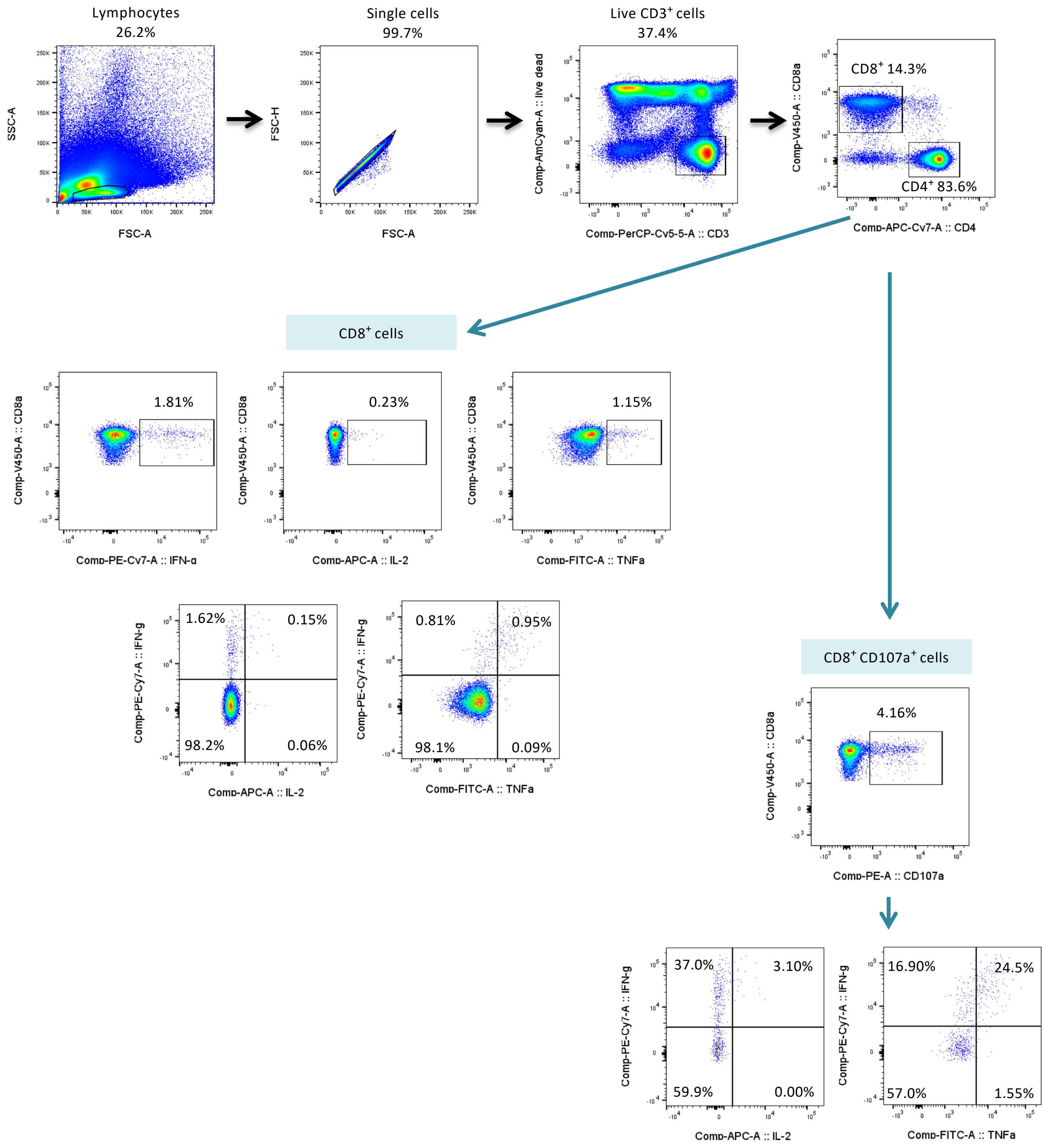

Figure S5 Gating strategy for the T-cell functional phenotype analysis.

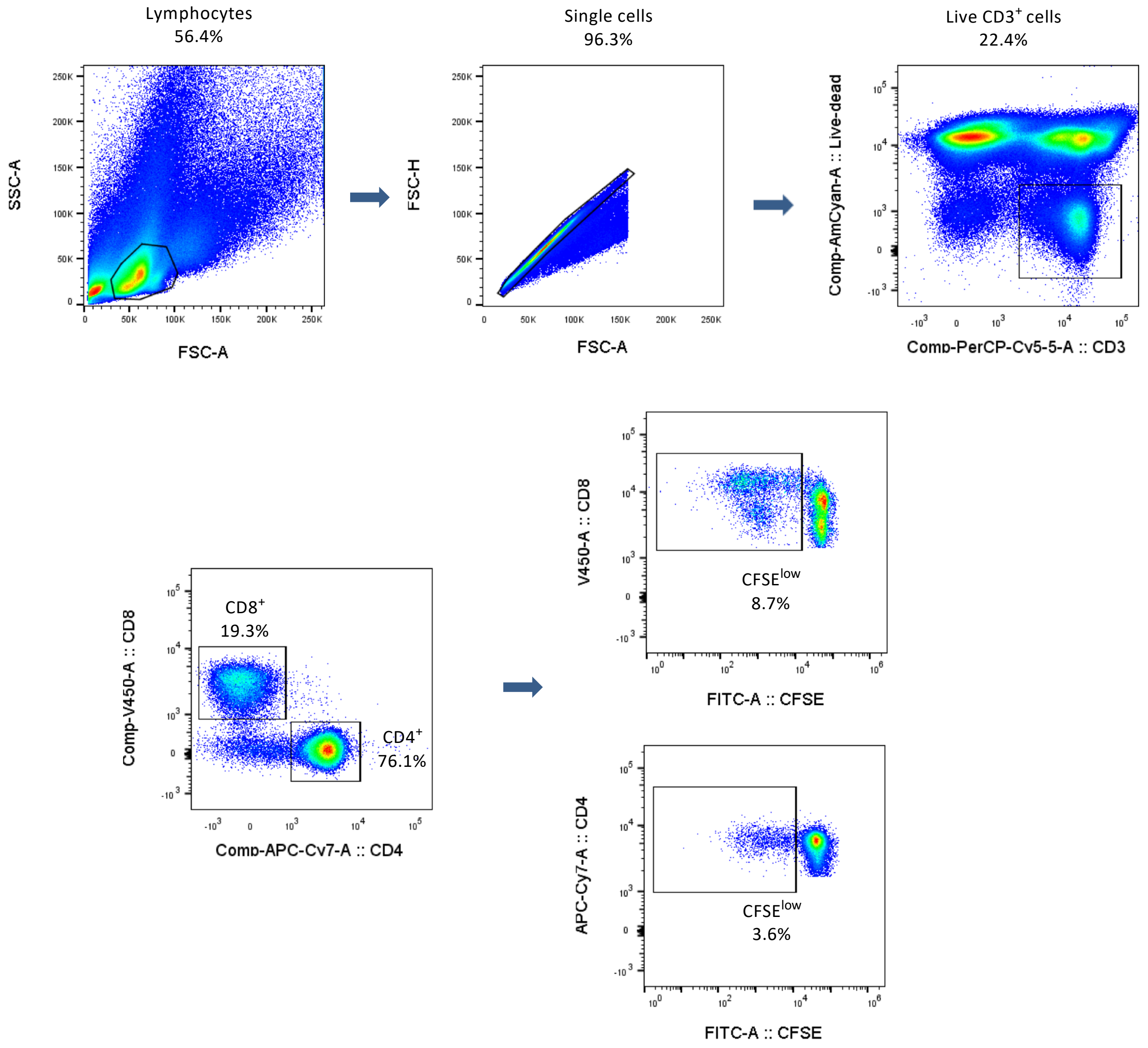

Figure S6 Gating strategy for the CFSE proliferation assay.

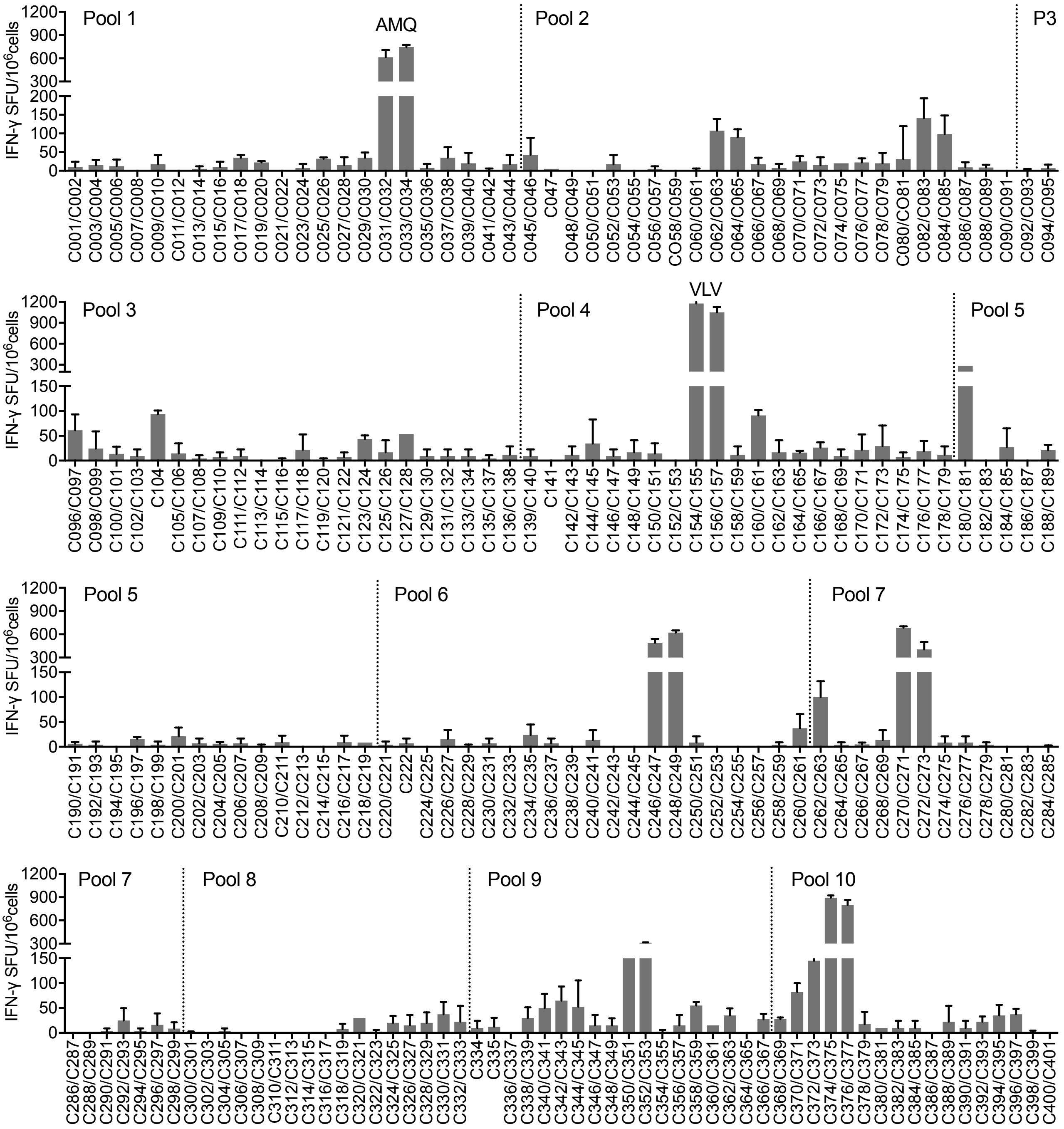

Figure S7 RNA-MVA-elicited responses to individual peptide pairs across the tHIVconsvX immunogens. Groups of BALB/c mice were immunized with AIR.tHIVconsv1+AIR.tHIVconsv2 saRNA followed by MVA.tHIVconsv3+MVA.tHIVconsv4, sacrificed 1 week later and their splenocytes were assessed in an IFN-γ ELSPOT assay using individual 15-mer peptide pairs, whereby one peptide was from mosaic 1 and the other peptide was derived from mosaic 2 (See Figure S3 for the peptide sequences).

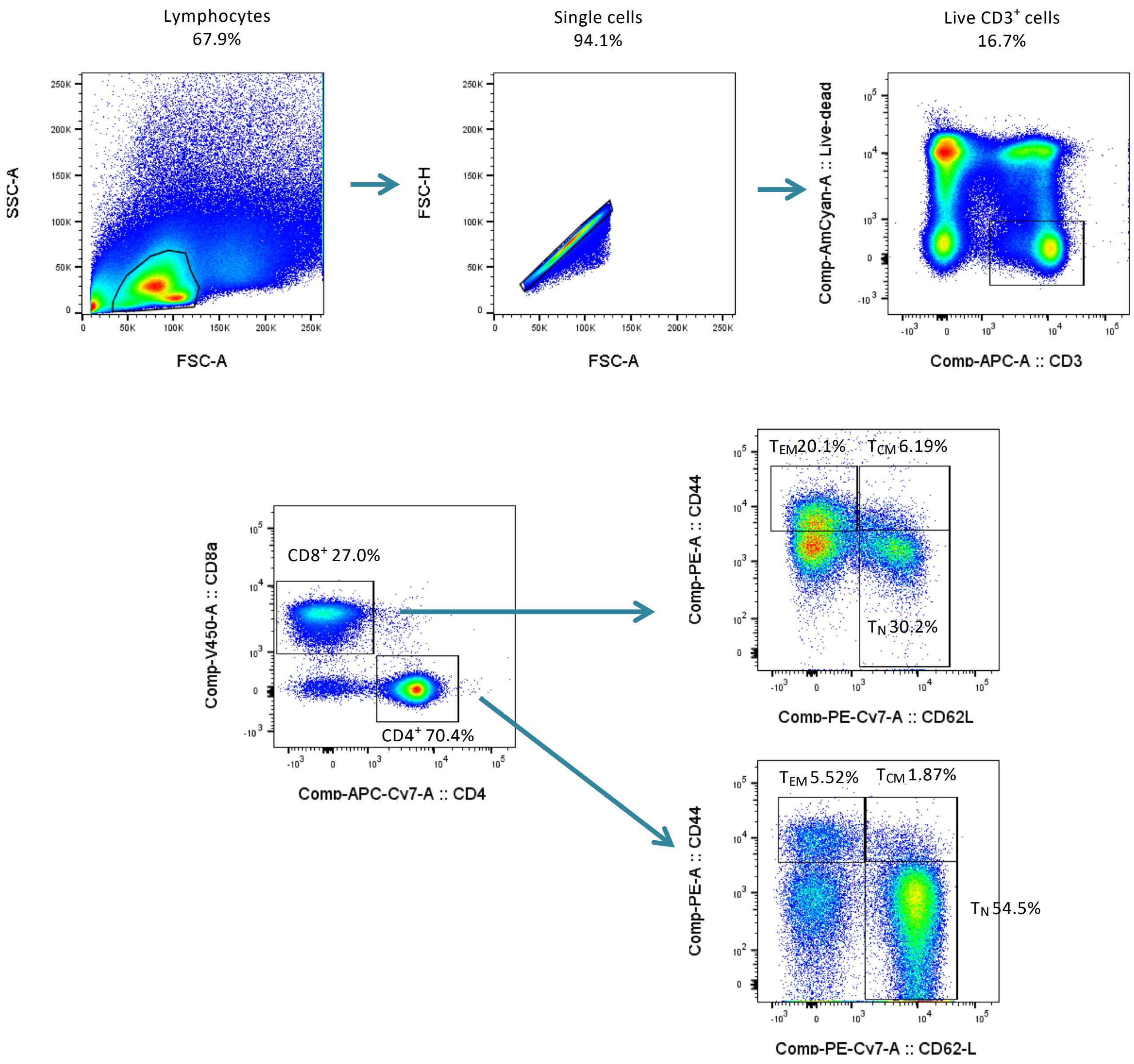

Figure S8 Gating strategy for the T-cell memory subtype analysis.
