# Supplementary material for: Efficient Induction of T Cells against Conserved HIV-1 Regions by Mosaic Vaccines Delivered as Self-Amplifying mRNA
Source: Mol Ther Methods Clin Dev. 2018 Oct 26;12:32–46. doi: 10.1016/j.omtm.2018.10.010 (PMC6258890; doi:10.1016/j.omtm.2018.10.010)
Supplement: Document S2. Article plus Supplemental Information [file mmc2.pdf]

Molecular Therapy
Methods & Clinical Development
Original Article

AMERICAN SOCIETY of GENE & CELL THERAPY

Efficient Induction of T Cells against Conserved HIV-1 Regions by Mosaic Vaccines Delivered as Self-Amplifying mRNA

Nathifa Moyo,[1] Annette B. Vogel,[2] Søren Buus,[3] Stephanie Erbar,[2] Edmund G. Wee,[1] Ugur Sahin,[2] and Tomáš Hanke[1,4]

[1]The Jenner Institute, Nuffield Department of Medicine, University of Oxford, Oxford OX3 7DQ, UK; [2]Biopharmaceutical New Technologies (BioNTech) Corporation, Mainz 55131, Germany; [3]Department of Immunology and Microbiology, University of Copenhagen, Copenhagen 2200, Denmark; [4]International Research Center for Medical Sciences, Kumamoto University, Kumamoto 860-0811, Japan

Focusing T cell responses on the most vulnerable parts of HIV-1, the functionally conserved regions of HIV-1 proteins, is likely a key prerequisite for vaccine success. For a T cell vaccine to efficiently control HIV-1 replication, the vaccine-elicited individual CD8$^+$ T cells and as a population have to display a number of critical traits. If any one of these traits is suboptimal, the vaccine is likely to fail. Fine-tuning of individual protective characteristics of T cells will require iterative stepwise improvements in clinical trials. Although the second-generation tHIVconsvX immunogens direct CD8$^+$ T cells to predominantly protective and conserved epitopes, in the present work, we have used formulated self-amplifying mRNA (saRNA) to deliver tHIVconsvX to the immune system. We demonstrated in BALB/c and outbred mice that regimens employing saRNA vaccines induced broadly specific, plurifunctional CD8$^+$ and CD4$^+$ T cells, which displayed structured memory subpopulations and were maintained at relatively high frequencies over at least 22 weeks post-administration. This is one of the first thorough analyses of mRNA vaccine-elicited T cell responses. The combination of tHIVconsvX immunogens and the highly versatile and easily manufacturable saRNA platform may provide a long-awaited opportunity to define and optimize induction of truly protective CD8$^+$ T cell parameters in human volunteers.

INTRODUCTION

Control of the HIV-1 epidemic remains one of the leading global health priorities. Remarkable gains have been achieved in decreasing HIV-1 transmission and AIDS-related deaths due to development of over 30 antiretroviral drugs.[1] However, still almost half of people who are HIV-1 positive are unaware of their status. In addition, antiretroviral drugs are not available on a regular reliable basis in many resource-poor settings, their effective administration requires rigorous daily compliance,[2,3] there are toxicities associated with their long-term use,[4–6] and viruses develop resistance. Also, there is unwillingness to take drugs in a surprisingly large proportion of infected e.g., adolescent, individuals. Thus, an effective, prophylactic HIV-1 vaccine will always be the best solution and possibly key to any strategy for halting the AIDS epidemic.[7] For the most efficient control of HIV-1, a vaccine will likely have to induce both broadly neutralizing antibodies and effective CD8$^+$ T cells.[8] Our aim is to understand and induce protective T cell responses, which will have a role in control of HIV-1 following initial transmission and in HIV-1 cure.

We have pioneered a T cell vaccine strategy, which employs highly conserved regions of the HIV-1 proteome.[9] The first-generation immunogen HIVconsv uses 14 regions designed as a clade-alternating consensus.[10] HIVconsv was tested extensively in pre-clinical settings.[11–19] To date in regimens involving plasmid DNA, simian (chimpanzee) adenovirus (ChAdV-63), and poxvirus-modified vaccinia virus Ankara (MVA), the HIVconsv vaccines have been tested in eight clinical trials, showed promising immunogenicity and *in vitro* control of replication of four major clades of HIV-1 and, in combination with latency-reverting agent, produced a signal of viremic control during monitored antiretroviral treatment (ART) pause in early treated patients (Fidler et al., 2018, Intern. AIDS Soc., abstract; Mothe et al., 2017, Intern. Antivir. Soc., abstract; B. Mothe, C. Manzardo, A. Snachez-Bernabeau, P. Coll, S. Moron-Lopez, M.C. Puertas, M. Rosas, P. Cobarsi, R. Escrig, N. Perez-Alvarez, I. Ruiz, C. Rovira, M. Meulbroek, A. Crook, N. Bothwick, E.G. Wee, H. Yang, J.M. Miró, L. Dorrell, B. Clotet, J. Martinez, Picado, C. Brander, and T.H., unpublished data).[20–25] Six immunogens of the second generation, collectively designated tHIVconsvX, further improved the first-generation conserved-region design by bioinformatics-assisted definition of conserved regions, including protective epitopes defined in patient cohorts on four continents and maximizing a perfect potential T cell epitope match of the vaccines to the circulating global HIV-1 isolates through using a bivalent

Received 24 August 2018; accepted 21 October 2018;
https://doi.org/10.1016/j.omtm.2018.10.010.

Correspondence: Tomáš Hanke, The Jenner Institute, Nuffield Department of Medicine, University of Oxford, Old Road Campus Research Building, Roosevelt Drive, Oxford OX3 7DQ, UK.
E-mail: tomas.hanke@ndm.ox.ac.uk

 © 2019 The Authors.
This is an open access article under the CC BY license (http://creativecommons.org/licenses/by/4.0/).

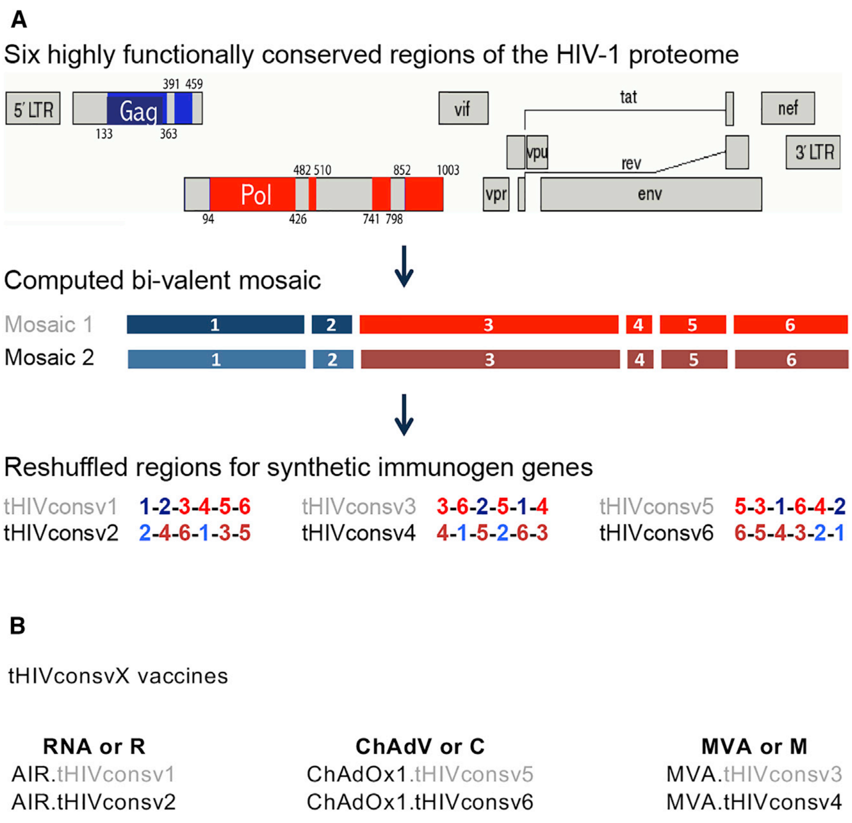

Figure 1. The tHIVconsvX Vaccines

(A) Curated full-protein amino-acid HIV-1 sequences present in the LANL-HSD database (September 2013) were used to compute bivalent mosaic and select 6 highly conserved regions of the HIV-1 proteome.[26] Mosaic 1 and mosaic 2 (color-coded) differ in approximately 10% of amino acids and together maximize match of the vaccines to globally circulating HIV-1 group M isolates. Each tHIVconsvX vaccine component of the multi-component regimens uses a different order of the 6 conserved regions to minimize induction of T cell responses to potential neo-epitopes irrelevant for HIV-1 generated by two juxtaposed regions.[20] (B) Vaccines used in this work. The 6 differentially ordered transgenes were inserted into vaccine vectors saRNA (RNA or R), non-replicating simian adenovirus ChAdOx1 (ChAdV or C), and non-replicating poxvirus (MVA or M). Unless specified, for each immunization, both mosaics were used together.

mosaic.[26] The second-generation immunogens delivered by DNA, ChAdOx1, MVA, and integration-defective lentivirus vectors demonstrated good immunogenicity in animal models,[26,27] and recombinant ChAdOx1 and MVA are in the pipeline to enter human trials.

It is our belief that eventual development of effective vaccines against HIV-1 is more likely to happen through iterative multiple small but significant steps forward rather than a new “out-of-box” idea. The most relevant developments will always be those made in human trials, where acceleration of iterative improvements will be greatly facilitated by easily adaptable, affordable, and quickly manufacturable vaccine modalities. One such vector currently in the spotlight is mRNA. The use of naked RNA molecules was hampered for a long time by its instability, inefficient crossing of the cell membrane, and potent induction of innate responses, which, e.g., cease cellular translation.[28,29] Over the last decade, there have been great leaps toward solving these challenges through structural and chemical modifications to the RNA molecule itself,[30–36] formulation into various nanoparticles or nanoemulsion,[33,37–39] and use of polymers and conjugation.[40,41] These advances enhanced by the excellent safety features of mRNA vaccines, and their fully synthetic and relatively cheap, fast, and scalable GMP manufacture have generated lots of hopes and indeed investment into this emerging platform.[42] mRNA vaccines in pre-clinical models showed protective efficacy against a number of viruses, such as influenza, rabies, Ebola, and Zika,[37,39,43] and an increasing list of preventive and therapeutic vaccine candidates have entered clinical evaluation in humans for cancer, allergy, and infectious diseases.[44] Although mRNA vaccines to date focused not exclusively but predominantly on induction of neutralizing antibodies, in the present work, we explore the potential of polymer-formulated self-amplifying mRNA (saRNA) vaccines to induce alone and in a combination with other vaccine vectors $CD8^+$ T cell responses. The results are discussed in the context of the current state of HIV-1 vaccine development.

RESULTS

Design and Construction of the AIR.tHIVconsv1 and AIR.tHIVconsv2 Candidate Vaccines

Novel candidate HIV-1 vaccines were vectored by a self-amplifying RNA-based Ribological RNA amplicon derived from Semliki Forest virus (SFV),[45] which was developed by BioNTech and designated Amplified Immune Response (AIR). AIR.tHIVconsv1 and AIR.tHIVconsv2 mRNA vaccines express the second-generation conserved-region immunogens tHIVconsv1 and tHIVconsv2, respectively (Figure 1). These immunogens consist of 6 highly conserved regions of the HIV-1 Gag and Pol proteins computed into a bivalent mutually complementing mosaic 1 and mosaic 2,[26] which are two versions of the same regions differing in approximately 10% of amino acids (Figure 1A). Mosaic 1 (tHIVconsv1&3&5) and mosaic 2 (tHIVconsv2&4&6) are always co-administered together for each vaccine dosing, whereby any mosaic 1 can pair with any mosaic 2 without compromising the regimen’s immunogenicity. Note that the choice of tHIVconsvX immunogens for vectors was arbitrary. tHIVconsv5&6 and tHIVconsv3&4 were inserted into ChAdOx1 and MVA, respectively[26]; thus, tHIVconsv1&2 were used for other vaccine modalities (Figure 1B). All genes coding for the tHIVconsvX immunogens employ humanized codons. After a completely cell-free *in vitro* transcription, the

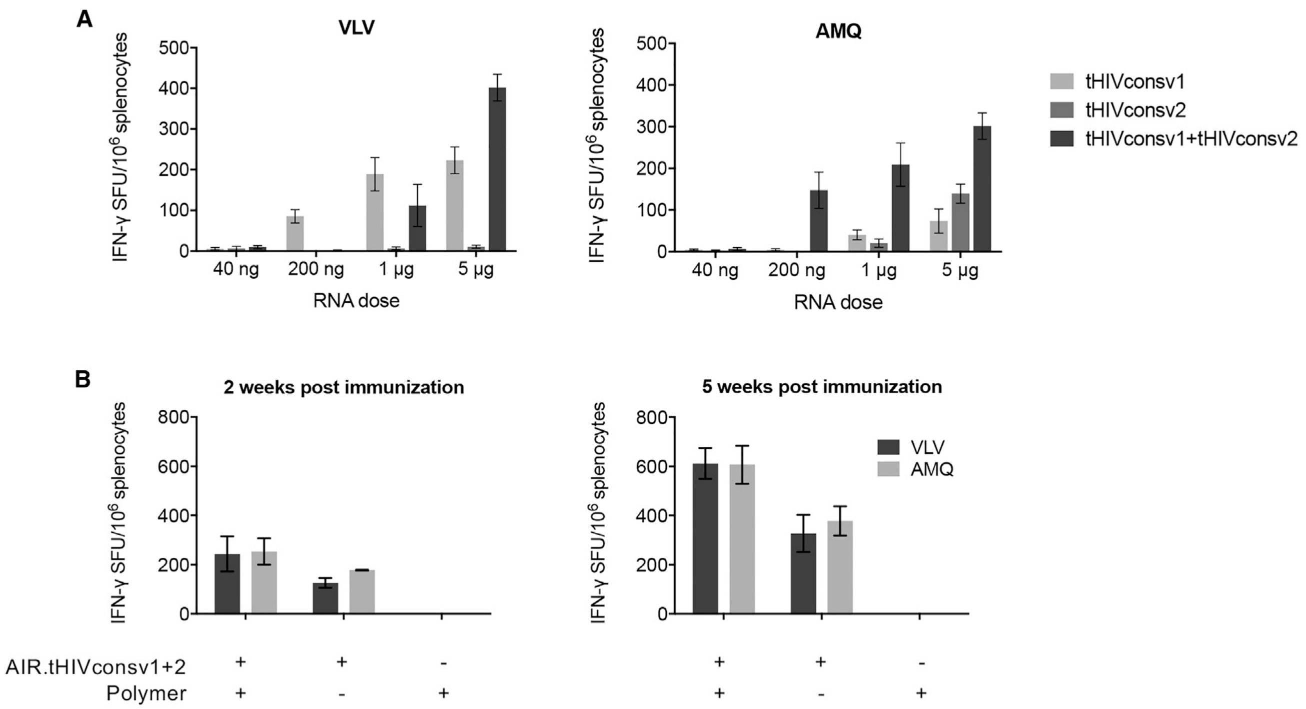

Figure 2. Dose Response of Mosaic saRNA Vaccines

(A) Groups of BALB/c mice were administered increasing vaccine doses of either individual mosaic vaccines or their half-dose combination. (B) Effect of saRNA formulation. A single saRNA dose of AIR.tHIVconsv1 + AIR.tHIVconsv2, 5 μg in total, was administered to mice with or without polymer and also polymer alone was used to assess the immunogenicity of each vaccine component. Mice were sacrificed either 2 or 5 weeks after vaccination (Figure S1). For both panels, vaccine-elicited T cell responses in the spleen were enumerated in an IFN-γ ELISPOT assay, performed in triplicate, using two immunodominant peptide pairs VLV and AMQ; the mean of the triplicates for each mouse was calculated. Data are shown as median ± interquartile range (IQR) of the means for n = 3 mice per group.

saRNA was purified and polymer formulated for enhanced cellular delivery and translation rate. The construction of the viral vaccines was described previously.[26]

saRNA Vaccine Dose Optimization for T Cell Induction

All vaccine dosings were delivered by intramuscular needle injection into the quadriceps muscle of the hind legs. BALB/c mice were injected with a total of 40 ng, 200 ng, 1μg, and 5 μg of saRNA of either AIR.tHIVconsv1 alone, AIR.tHIVconsv2 alone, or as two half-doses of AIR.tHIVconsv1 + AIR.tHIVconsv2 together. We took advantage of two known H-2^d immunodominant epitopes AMQ (AMQMLKD/ETI; pool P1) and VLV (VLV/IGPTPVNI; pool P4)[17,27] and used only those two peptide pairs, one from each mosaic, in an interferon γ (IFN-γ) ELISPOT assay for enumeration of vaccine-elicited responses. We found that, 1 week after administration, the saRNA vaccines induced only weak responses below 50 spot-forming units (SFUs)/10^6 splenocytes (Figure S2). It was through analysis of the kinetics of the T cell response induction shown below that we realized that, following saRNA vaccination, T cell frequencies keep increasing beyond the first week, which is the peak time for many viral vectors. Thus, the same dose-response experiment was repeated, but this time, mice were sacrificed 5 weeks post-vaccination. First, we confirmed the immunogenicity of both AIR.tHIVconsv1 and AIR.tHIVconsv2 individually (Figure 2A). For the combined half-doses, the frequencies of specific T cells were increasing up to 5 μg dose, and the total of 5 μg of saRNA (2.5 μg AIR.tHIVconsv1 + 2.5 μg AIR.tHIVconsv2) was chosen as the standard intramuscular dose. Next, we assessed the importance of the saRNA vaccine polymer formulation. Although complexing saRNA with polymer approximately doubled the elicited T cell frequencies relative to naked saRNA and reached approximately 600 IFN-γ SFUs/10^6 splenocytes for both the VLV and AMQ epitopes at 5 weeks post-administration, the polymer without saRNA failed to induce any HIV-1-specific responses (Figure 2B).

Characterization of T Cells Induced by a Single saRNA Dose

Next, we characterized the T cell responses induced by a single saRNA dose. Groups of BALB/c mice were immunized using 5 μg of saRNA of the single or combined vaccines, sacrificed weekly between 1 and 5 weeks post-delivery, and the vaccine-elicited frequencies were enumerated using the two VLV and AMQ pairs of peptides. First, each mosaic induced different frequencies of VLV- and AMQ-specific CD8$^+$ T cells depending on whether it contained immunodominant or subdominant epitope variants. Notably for the late time points, the mixed delivery of AIR.tHIVconsv1 + AIR.tHIVconsv2 was not additive in respect to the T cell frequencies induced by individual mosaics but synergized (Figure 3A). Second, the frequencies of

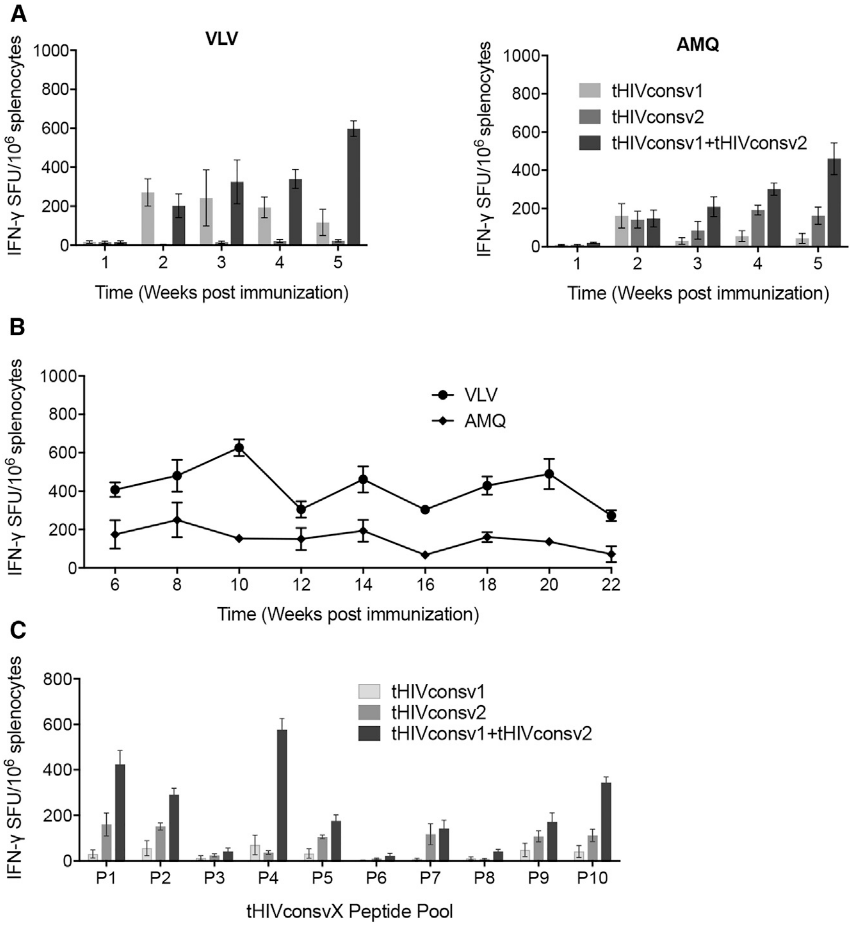

Figure 3. Induction of tHIVconsvX-Specific T Cells by a Single Dose of the saRNA Vaccine

Groups of BALB/c mice were immunized with 5 μg of formulated AIR.tHIVconsv1, AIR.tHIVconsv2, or half-doses of AIR.tHIVconsv1 + AIR.tHIVconsv2 (Figure S1), and the vaccine-elicited responses were analyzed using the IFN-γ ELISPOT assay, performed in triplicate; the mean of the triplicates for each mouse was calculated. (A and B) Kinetics of T cell responses between weeks 1 and 5 (A) and weeks 6 and 22 (B) was examined employing two immunodominant peptide pairs VLV and AMQ indicated above the graphs. The latter interval only employed the combined vaccine. (C) Splenocytes from 5 weeks after saRNA vaccination were tested for recognition of ten peptide pools P1–P10 across the entire length of the tHIVconsvX immunogen to provide the total magnitude and initial estimation of the response breadth. All data are shown as median ± IQR of the means for n = 5 mice per group.

tHIVconsvX T cells increased with time up to 5 weeks post-delivery. This observation prompted an extended experiment, which demonstrated sustained levels of the T cell frequencies following a single vaccine delivery until week 22 (Figure 3B). At week 5, we also assessed the breath of saRNA-induced T cell responses by using 10 peptide pools P1–P10 across the entire length of the two mosaic immunogens and also individual peptide pairs (Figure S3). The saRNA-vaccine-elicited T cells recognized 17 peptide pairs (Figure S4) in 8 peptide pools, confirming induction of broad "immunodemocratic" (broad of similar magnitude) responses (Figure 3C). Next, we examined the evolution of T cell functions at 2, 12, and 22 weeks after a single vaccine administration, assessing upon specific-peptide stimulation the production of IFN-γ, tumor necrosis factor α (TNF-α), interleukin 2 (IL-2), and degranulation measured by surface expression of CD107a, which correlates well with cytolytic activity[46,47] in an intracellular cytokine staining assay (ICS). All four functions were detectable for $CD8^+$ T cells, and $CD4^+$ T cells displayed only three functions because they typically do not degranulate (Figure 4A). In broad agreement with the IFN-γ ELISPOT data, the functionality was the highest of the three time points measured at week 12. Putting all these parameters together for single cells, we found over two-thirds of cells to be monofunctional and 8%–9% trifunctional $CD8^+$ T cells detected at weeks 12 and 22 (Figure 4B). Both $CD8^+$ and $CD4^+$ T cells showed no proliferation at 2 weeks, peaked at 12 weeks post-vaccination, and declined thereafter (Figure 4C). Finally, at the same late time points, both $CD8^+$ and $CD4^+$ T cells showed structured memory phenotypes with significant proportions of effector memory ($CD44^{hi}CD62L^{lo}$) and central memory ($CD44^{hi}CD62L^{hi}$) T cells and, for $CD8^+$ T cells, a smaller fraction of naive T cells ($CD44^{lo}CD62L^{hi}$; Figure 4D). Thus, the AIR.tHIVconsv1 + AIR.tHIVconsv2 saRNA vaccines induce conserved region-specific $CD8^+$ and $CD4^+$ T cells with desirable phenotypic properties.

Optimal Timing for Homologous saRNA Boost

Given the unusual kinetics of induction and long persistence of the saRNA-elicited T cell responses, we set out to carefully determine the optimal time for a homologous saRNA boost. Groups of BALB/c mice were immunized twice with a 2-, 4-, and 6-week gap between the prime and boost administrations and sacrificed 4, 8, or 12 weeks later. Using the IFN-γ ELISPOT assay and the BALB/c pool of 17 pairs of responder peptides, both splenocytes and peripheral blood mononuclear cells (PBMCs) gave similar results, and there was no significant difference between the T cell frequencies among the three gaps with the exception of week 8 post-administration, whereby the difference between a 2- and 6-week gap reached significance ($p < 0.05$). For all gaps, specific T cell frequencies declined by 12 weeks post-boost (Figure 5A). This broadly agreed with the frequencies of IFN-γ-producing cells measured using ICS, which again did not differ among the three prime-boost gaps. However, there were peak frequencies of IFN-γ^+ cells at 4 weeks post-immunization, which declined thereafter (Figure 5B). The differences observed among gaps were in the $CD8^+$ T cell CD107a expression, which peaked for a 4-week gap at

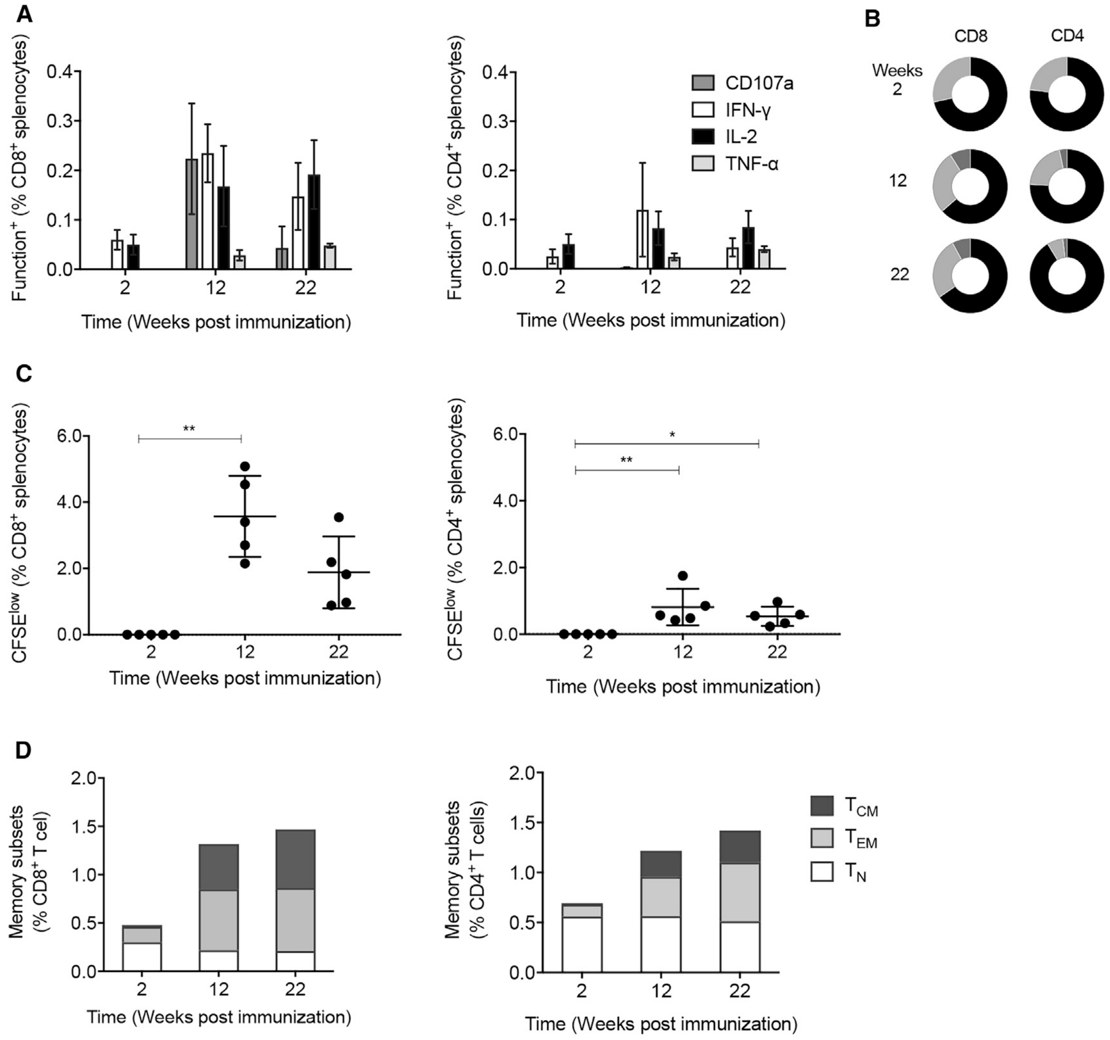

Figure 4. Evolution of saRNA-Induced T Cell Functions and Memory Subtypes

Groups of BALB/c mice were immunized with total of 5 μg of formulated AIR.tHIVconsv1 + AIR.tHIVconsv2 (Figure S1), and the vaccine-elicited responses were analyzed using polychromatic flow cytometry at weeks 2, 12, and 22. (A) Total frequencies of cells with indicated functions at 3 time points post-immunization are shown for vaccine-elicited $CD8^+$ and $CD4^+$ T cells in graphs, and (B) the pie charts provide the plurifunctionality, whereby 1 (black)—one function; 2 (light gray)—two functions; and 3 (dark gray)—three functions (see Figure S5 for the gating strategy). (C) Proliferative capacity assessed in a CFSE-dilution assay (see Figure S7 for the gating strategy) is shown. (D) Memory subtypes in total $CD8^+$ and $CD4^+$ T cells responding to the BALB/c peptide pool by expression of CD44 and CD62-L (see Figure S6 for the gating strategy) are shown. T_{CM}, central memory ($CD44^{hi}CD62L^{hi}$); T_{EM}, effector memory ($CD44^{hi}CD62L^{lo}$); T_N, naive T cells ($CD44^{lo}CD62L^{hi}$). The expression of indicated markers was analyzed in duplicate; the mean of the duplicates for each mouse was calculated. In (A) and (C), data are shown as median ± IQR and in (B) and (D) are shown as the median of the means for n = 5 mice per group. Results were analyzed using the Mann-Whitney U test for comparison between the long-term samples versus the early time point. Significant p values are indicated by asterisks: *$p < 0.05$; **$p < 0.01$.

8 weeks post-boost. We also noted the generally low production of TNF-α, the promoter of inflammation, apoptosis, and immunity.[48–50] For $CD4^+$ T cell IL-2 production, there was a peak for the 6-week gap at 4 weeks after saRNA boost, which was much less pronounced for the shorter gaps (Figure 5B). There were not any obvious differences among the three gaps in the number of functions displayed by individual $CD8^+$ and $CD4^+$ T cells, with the highest plurifunctionality measured 4 weeks post-vaccination (Figure 5C). The best proliferative capacity was observed for a 4-week gap (Figure 5D). Finally, we determined the structure of memory subtypes. The highest frequencies of T cells were detected for the 4-week gap at 8 weeks after vaccination, reaching 6% and 4% of $CD8^+$ and $CD4^+$ T cells, respectively, and effector memory T cells were the predominant memory subtype (Figure 5E). Overall, homologous saRNA boosts between 2 and 6 weeks after the saRNA priming induced very similar frequencies and qualities of effector $CD8^+$ and $CD4^+$ T cells. Possibly the regimen of choice would deliver two saRNA doses 4 weeks apart, and the T cells would be expected to peak between 4 and 8 weeks later.

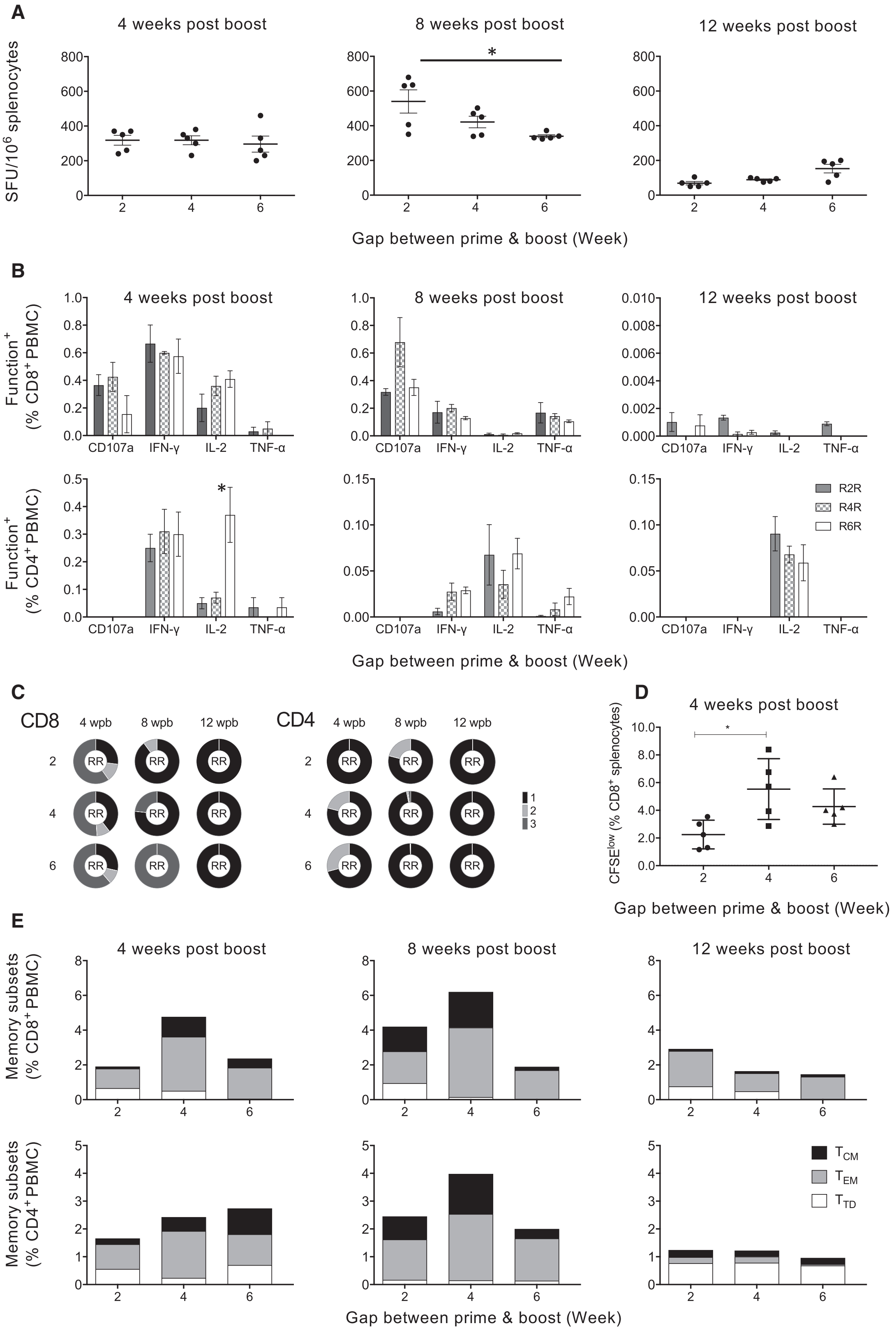

(legend on next page)

saRNA Prime Combines Well with Heterologous Viral Vector Boost

Heterologous prime boost combinations of non-replicating subunit genetic vaccines are currently the leading strategies for induction of robust anti-microbial T cell responses in humans.[8,9,51] Here, single deliveries of saRNA (RNA), ChAdOx1 (ChAdV), and MVA were compared with homologous boost (RNA-RNA) and heterologous boosts with MVA (RNA-MVA) and ChAdOx1 (RNA-ChAdV) delivering the same bivalent-mosaic tHIVconsvX immunogens. IFN-γ ELISPOT assay employing the ten peptide pools P1–P10, BALB/c pool, and immunodominant peptide pairs VLV and AMQ to assess CD8$^+$ T cell induction, whereby the VLV/AMQ readout was from separately immunized mice. High frequencies of tHIVconsvX-specific T cells were detected with the RNA-RNA < RNA-ChAdV < RNA-MVA hierarchy peaking for the last at median of 9,343 tHIVconsvX-specific SFU/10^6 splenocytes (Figure 6A). Thus, the highest frequencies were induced by the RNA-MVA regimen.

The CD8$^+$ T Cell Response Breadth and Specificity

The most efficient regimen for induction of CD8$^+$ T cells was AIR.tHIVconsv1 + AIR-tHIVconsv2 prime and MVA.tHIVconsv3 + MVA.tHIVconsv4 boost, or RNA-MVA, and was used to assess the breadth of the responses and map the stimulatory peptides in the BALB/c mice. Overall, responses over 50 SFUs/10^6 splenocytes were induced to 25 15-mer peptide pairs and one 15-mer common between the two mosaics, which likely corresponded to at least 16 CD8$^+$ and/or CD4$^+$ T cell epitopes considering the peptide overlap (Figures 6B and S7). Although this analysis confirmed high frequencies of T cells responding to VLV and AMQ, strong responses were also detected to other known epitopes, although many of the epitope variants used here were not previously described (Figure 6C). We used the NetMHCpan 4.0 software to predict optimal binder peptides for the three major histocompatibility complex (MHC) class I H-2^d alleles and confirmed recognition of 19 out of 20 in an IFN-γ ELISPOT assay by RM-immune splenocytes (Figure S8). Refolding of stable MHC-peptide monomers confirmed the allele restriction for AMQMLKETI/H-2K^d, VGPTPVNII/H-2D^d, and REHLLKWGF/H-2L^d and showed tetramer reactivity with RNA-MVA-immune CD8$^+$ splenocytes (Figure 6D). Overall, we demonstrated recognition of 11 novel HIV-1-derived epitope variants in the H-2^d haplotype. Finally, two variants of the AMQ and VLV epitopes were pulsed individually and the target cells were differentially labeled and transferred back into RNA and RNA-MVA vaccinated animals to assess the cell *in vivo* survival. This assay confirmed *in vivo* cytolytic activity of the vaccine-elicited effectors and indicated superiority of the heterologous regimen and immunodominance of AMQMLKETI over its AMQMLKDTI variant but similarity of the two VLV epitope variants (Figure 6E).

High Immunogenicity of the RNA-RNA and RNA-MVA Regimens in an Outbred Mouse Stock

Finally, we compared the performance of the RNA-RNA and RNA-MVA regimens in outbred animals. Groups of CD1-SWISS mice were primed with formulated AIR.tHIVconsv1 + AIR.tHIVconsv2 and boosted with the same or MVA.tHIVconsv3 + MVA.tHIVconsvsv4 using optimal timing for both regimens. Characterizing the vaccine-elicited responses in splenocytes using the IFN-γ ELISPOT assays, the RNA-RNA and RNA-MVA regimens induced total magnitudes (as sum of individual pool responses) of 1,868 and 5,411 SFUs/10^6 splenocytes, respectively. For the RNA-RNA regimen, all 5 mice responded to 7 pools (P1, P3, P4, and P7–P10), only 1 mouse responded to 2 pools (P5 and P6), and 1 pool (P2) remained without any responder. For the RNA-MVA regimen, all pools had at least one responding animal, whereby all 7 mice responded to 5 pools (P1, P3, P6, P7, and P10) and varying numbers responded to the remaining pools: 1 (P2); 3 (P4); 4 (P5); 6 (P8); and 2 (P9; Figure 7A). Although the overall magnitudes may reflect the overall potency of the regimens, individual pool differences in the number of responding animals is likely attributed to the differences in individual animal haplotypes. All four functions IFN-γ, IL-2, TNF-α, and CD107a were detected in CD8$^+$ T cells in blood and spleen, and CD4$^+$ T cells lacked degranulation (Figure 7B). The overall functionality of tHIVconsvX-specific CD8$^+$ T cell responses were similar between the two regimens (Figure 7B) and so were the T cell memory subtypes (Figure 7C). Thus, in outbred animals, the RNA-MVA regimen induced 2.9-fold higher frequency of HIV-1 conserved region-specific T cells relative to the RNA-RNA regimen, but the RNA-RNA responses were more plurifunctional.

DISCUSSION

In the present work, we characterized comprehensively the CD8$^+$ and CD4$^+$ T cell responses elicited in regimens involving formulated AIR saRNA-vectored subunit genetic vaccine candidates against HIV-1. In the BALB/c mice, we found that a single 5-μg saRNA dose of a bivalent-mosaic immunogen tHIVconsvX elicited over two thousand

Figure 5. Optimizing Repeated saRNA Vaccine Boost

Groups of BALB/c mice were immunized with 5 μg of formulated AIR.tHIVconsv1 + AIR.tHIVconsv2 (RNA or R) and boosted at 2, 4, or 6 weeks later with the same dose of the same vaccines. At 4, 8, and 12 weeks post-boost (Figure S1), the frequencies of responding PBMC and/or splenocytes were determined using one BALB/c pool of 17 responder peptide pairs in (A) an IFN-γ ELISPOT assay performed in triplicate and (B–E) multicolor flow cytometry analysis performed in duplicate; the mean of the triplicates or duplicates for each mouse was calculated and a panel of mAbs (B) used for the functional phenotypes and (C) the proportion of CD8$^+$ T cells (left panel) and CD4$^+$ T cells (right panel) expressing multiple functions are shown as pie charts, whereby the expression of one, two, or three markers is represented as 1 (black)—one function; 2 (light gray)—two functions; and 3 (dark gray)—three functions (see Figure S5 for the gating strategy); (D) CFSE proliferation assay and (E) using a panel of mAbs characterizing T cell memory subtypes is shown, whereby T_{CM}, central memory (CD44^{hi}CD62L^{hi}); T_{EM}, effector memory (CD44^{hi}CD62L^{lo}); and T_N, naive T cells (CD44^{lo}CD62L^{hi}). Data in (A), (B), and (D) are shown as median ± IQR of the means for n = 5 mice per group, and data in (C) and (E) are represented as median of the means for n = 5 mice per group. Groups were compared using two-tailed Mann-Whitney U tests, and two-tailed p values were used. Significant p values are indicated by asterisks: *$p < 0.05$.

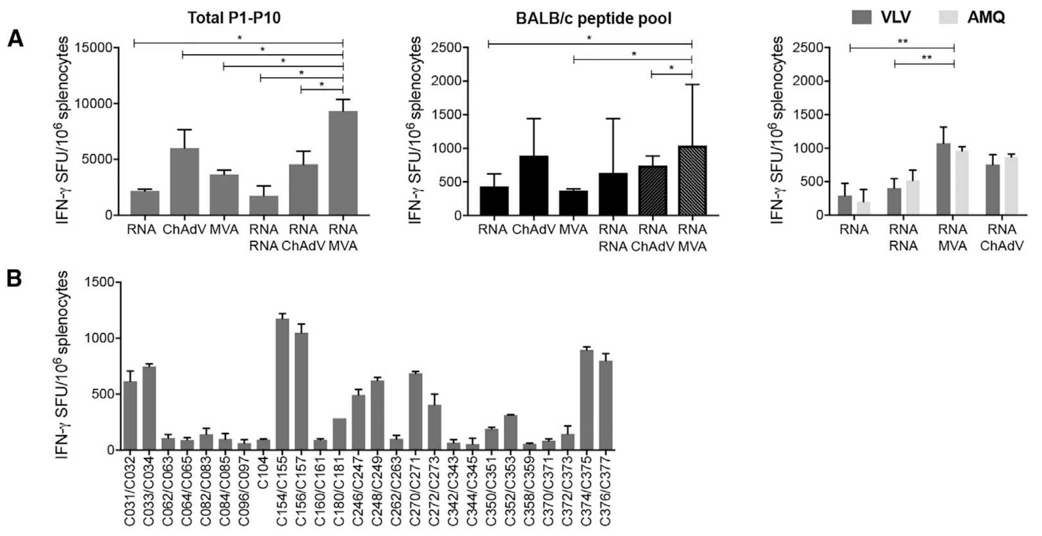

C

ID	15-mer Peptide	Known Epitope	Predicted Tested	SFU/10^6	Tetramer
C031	GHQAAMQMLKDTINE	GHQAAMQML	GHQAAMQML	200	Failed
C033	AMQMLKDTINEEAAE	AMQMLKETI	AMQMLKDTI	385	
C034	AMQMLKETINEEAAE	AMQMLKETI	AMQMLKETI	395	✔
C154	KAIGTVLVGPTPVNI	VGPTPVNI	VGPTPVNI	400	
C155	KAIGTVLIGPTPVNI	VGPTPVNI	IGPTPVNI	365	
C156	TVLVGPTPVNIIGRN	VGPTPVNII	VGPTPVNII	620	✔
C157	TVLIGPTPVNIIGRN	VGPTPVNII	IGPTPVNII	255	
C246	GSPAIFQSSMTKILE	IFQSSMTKI	IFQSSMTKI	275	
C247	GSPAIFQCSMTKILE	IFQSSMTKI	IFQCSMTKI	15	
		SPAIFQSSM	SPAIFQCSM	430	
C270	EELREHLLKWGFTTP	RQHLLRWGL	REHLLKWGF	480	✔
C271	EELRQHLLRWGFTTP	RQHLLRWGL	RQHLLRWGF	225	
C374	QNFRVYYRDSRDPIW	YYRDSRNPL	YYRDSRDPI	80	
		VYYRDSRNPL	VYYRDSRDPI	135	
		RVYYRDSRNPL	RVYYRDSRDPI	80	
		VYYRDSRNPLW	VYYRDSRDPIW	770	Failed
C375	QNFRVYYRDSRDPLW	YYRDSRNPL	YYRDSRDPL	90	
		RVYYRDSRNPL	RVYYRDSRDPL	55	
		VYYRDSRNPL	VYYRDSRDPL	250	

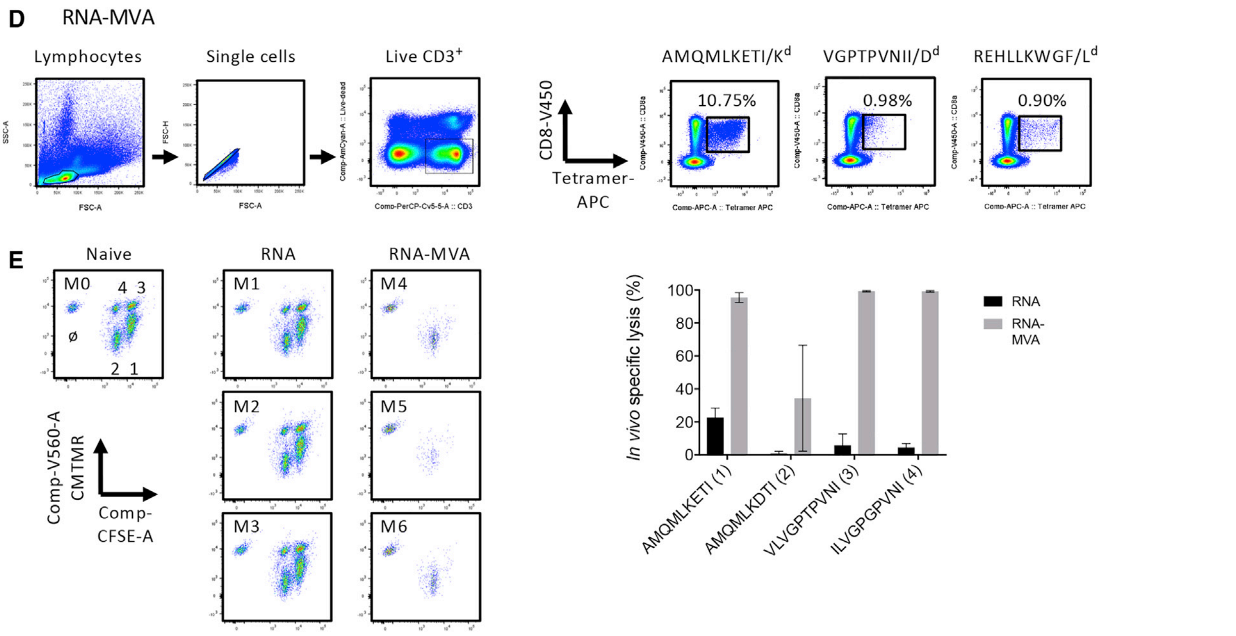

(legend on next page)

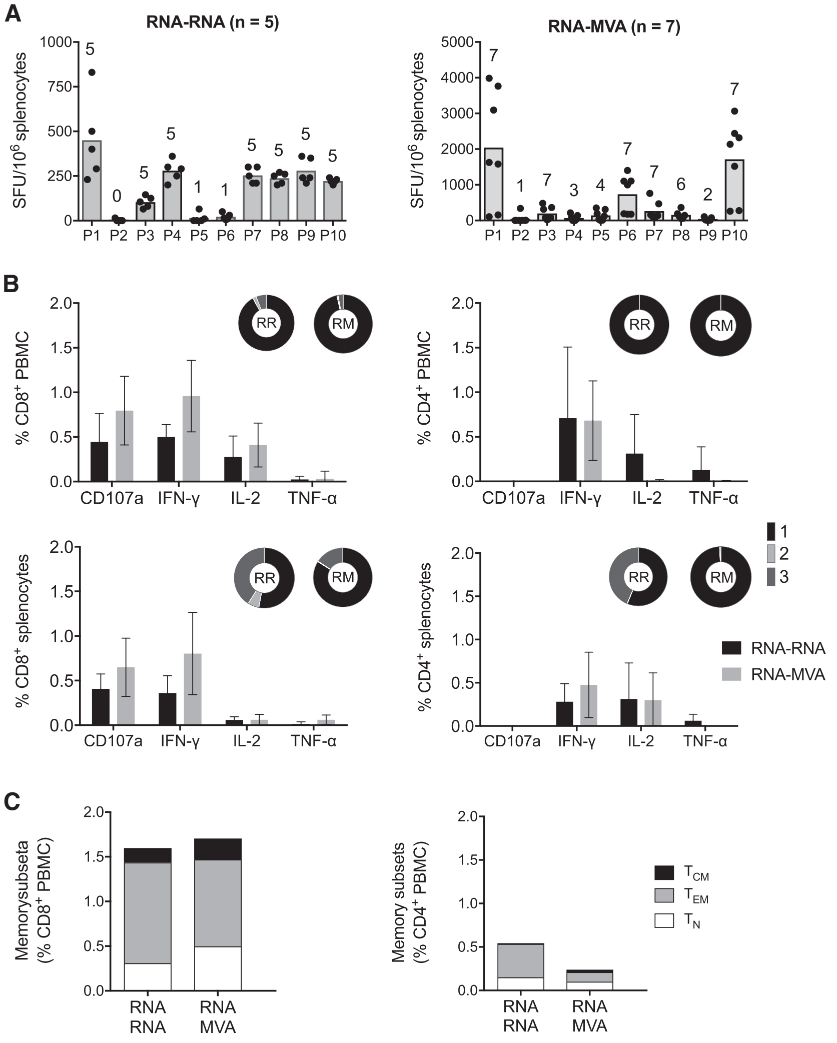

Figure 7. T Cell Induction in an Outbred Mouse Stock

CD1-SWISS mice were immunized with 5 μg of formulated AIR.tHIVconsv1 + AIR.tHIVconsv2, boosted at 4 or 2 weeks later with either the same (RR) or with a total of 5 × 10^6 PFUs of MVA.tHIVconsv3 + MVA.tHIVconsv4 (RM) vaccines and sacrificed at peak responses either at 4 or 1 week later, respectively (Figure S1). (A) INF-γ ELISPOT frequencies of T cells responding to tHIVconsvX peptide pools P1–P10 were measured in triplicates; the mean of the triplicates for each mouse was calculated. The number of animals responding to each pool is shown above bars. (B) The functionality and proportion of $CD8^+$ and $CD4^+$ T cells expressing multiple functions were assessed in an ICS assay using flow cytometry and performed in duplicate; the mean of the duplicates for each mouse was calculated, whereby 1 (black)—one function; 2 (light gray)—two functions; and 3 (dark gray)—three functions (see Figure S5 for the gating strategy). (C) Memory T cell subtypes were defined as T_{CM}, central memory ($CD44^{hi}CD62L^{hi}$); T_{EM}, effector memory ($CD44^{hi}CD62L^{lo}$); and T_N, naive T cells ($CD44^{lo}CD62L^{hi}$) and all samples were analyzed in duplicate; the mean of the duplicates for each mouse was calculated. Graph data in (A) and (B) are shown as median ± IQR of the means, and pies in (B) and graphs in (C) show median of the means only (RR: n = 5 and RM: n = 7 mice).

broadly specific T cells per million of splenocytes in total; these frequencies took at least 5 weeks to peak and were well maintained up to week 22 after vaccination. Furthermore, elicited T cells performed *in vitro* several effector functions upon peptide restimulation, killed *in vivo* epitope variants, and displayed structured memory subpopulations with well-represented effector and central memory. Homologous saRNA boost did not improve the induced T cell frequencies significantly, and no obvious effect of a time span between the prime and boost was readily discernible within the 2 and 6 weeks tested. In contrast, heterologous boosts with non-replicating virus vectors were able to at least double the HIV-1-specific T cell frequencies. The current superiority of the heterologous regimen over two saRNA deliveries was also demonstrated in a more rigorous model system of outbred animals. Thus, although a direct comparison of

Figure 6. saRNA Vaccine in Heterologous Regimens with Viral Vectors

(A) Groups of BALB/c mice were immunized using the indicated regimens (Figure S1). An IFN-γ ELISPOT assay, performed in triplicate, enumerated the elicited T cells using ten overlapping peptide pools P1–P10 and BALB/c pool (left and middle; n = 4 mice) and two pairs of immunodominant epitopes VLV and AMQMLKETI (right; n = 5 mice). The mean of the triplicates for each mouse was calculated. Data are shown as median + IQR of the means of "n" of mice per group. Groups were compared using Kruskal-Wallis test with Dunn's multiple comparison post-test. Significant p values are indicated by asterisks: *$p < 0.05$; **$p < 0.01$. (B) The RNA-MVA regimen was used to assess the breadth of T cell responses by using 201 individual peptide pairs of the P1–P10 pools (Figure S7). Only responses over 50 SFUs/10^6 splenocytes are measured in duplicate; the mean of the duplicates for each mouse was calculated and are data shown as median + IQR of the means for n = 5 mice per group. (C) Although all 26 stimulatory 15-mer peptides contained minimal epitopes present already in the LANL-HSD, in tHIVconsvX mosaics, 13 of these epitopes had unique amino acids (red) and recognition of 18 9-mers was confirmed. (C and D) For three minimal peptides, their H-2^d class I restriction was confirmed by refolding functional MHC class I/peptide monomers. These were assembled into tetramers and used for T cell analysis. (D) (Left) Gating strategy and (right) tetramer reactivity are shown. The best of 4 mice for each tetramer is shown to demonstrate the correct epitope/H-2 class I definition; all four animals had reactive splenocytes. Representative data are shown from a single analyzed mouse out of four mice. Data were acquired in duplicate. (E) *In vivo* killing. (Left) Differentially CMTMR/CFSE labeled and peptide-pulsed target cells transferred into naive (M0), RNA (M1–M3), or RNA-MVA (M4–M6) vaccinated BALB/c mice, re-isolated 10 hr later, and analyzed using flow cytometry are shown. Targets are as follows: 1—AMQMLKETI; 2—AMQMLKDTI; 3—VLVGPTPVNI; and 4—ILVGPGPVNI. (Right) % *in vivo* killing is shown. Flow cytometry plots are shown for all three mice. All data are analyzed in duplicate; the mean of the duplicates for each mouse was calculated and data in graphs shown as median ± IQR of the means for n = 3 mice per group.

self-amplifying and non-self-amplifying mRNA showed 64-fold higher immunogenicity of the former,[52] we see a great scope for all variations of this still very young platform to improve and shape future vaccine induction of both antibody and T cell responses.

We find the increase in T cell responses over 5 weeks and their long, sustained persistence intriguing. We are not aware of any studies suggesting, e.g., slow vaccine mRNA release from a depo, persistence of immunogenic protein, or requirement for any particular cell type uniquely explored by mRNA vaccines. We hypothesized that no immunogen is present for priming of T cell responses until after cell entry of saRNA and its translation. This would explain a delay of immune cell activation compared to protein- and/or live-virus-based treatments that already come with foreign and/or "danger" antigens. As saRNA translation begins, the immune responses can be generated with a steady trigger of ongoing protein expression up to the transfected cell death, which delivers another important trigger for immune response activation and releases immunogen for cross-presentation. Overall, we believe that these features generate a different immune response kinetic compared to other platforms. Further studies are warranted.

For HIV-1, there is a broad consensus on the importance of supporting development of vaccines aiming at induction of antibody-mediated protection. However, evidence of T-cell-mediated control of HIV-1 infection is ample. This comes from the temporal association of $CD8^+$ T cell appearance and control of primary viremia,[53–58] extensive virus escape in targeted epitopes,[55,59–61] association of certain HLA class I alleles with HIV-1 control *in vivo*,[54,59,60,62–64] and definition of protective $CD8^+$ T cell epitopes in patient cohorts.[21,26,65–67] Also model infection of rhesus macaques with simian immunodeficiency virus supports importance of $CD8^+$ T cells.[68–74] Thus, inducing highly effective T cell responses by vaccination is likely to help broadly neutralizing antibodies reduce acquisition of HIV-1, as achieving truly sterilizing protection will be challenging and may be central to HIV cure. However, definition of beneficial qualities and quantity of T cells remain elusive. This is because HIV-1-specific T cells are a heterogeneous population, which, in natural HIV-1 infection, target both protective and non-protective viral epitopes.[26,65,66] Thus, any attempts to associate a single $CD8^+$ T cell property with HIV-1 control have to be carried out in the context of T cell specificity. Furthermore, HIV-1 control likely depends on a number of critical T cell traits required to be optimal at the same time, and if any one of these traits is suboptimal, the T cells, and therefore vaccines, may fail to protect.[9,26] These important parameters include specificity for protective epitopes,[26,65,66] parallel recognition of multiple protective epitopes,[8,75,76] optimal interaction with HLA-peptide complexes,[77] rapid proliferation upon exposure to cognate peptides to reach protective numbers,[78,79] lysis of infected cells, and production of soluble antiviral and intercellular signaling molecules.[78–81] The $CD8^+$ T cells induced by regimens involving saRNA displayed in the mouse model a number of the desired properties. Whether or not these will translate to humans and be beneficial remains to be seen, but the presented data encourage and deserve such translational studies. The BALB/c inbred mouse strain allowed detailed, sophisticated T cell analyses, and the CD1-SWISS mice demonstrated vaccine elicitation of desired responses in an outbred animal stock closer to a human population. However, many critical costimulatory signals, including stimulation through the toll-like receptors, differ between mice and humans, and therefore, having shown here the great potential of the saRNA technology to stimulate T cell responses, further optimization for human vaccines has to be carried out in humans. It follows that, to advance our understanding of T cell protection against HIV-1 infection, we need to measure multiple parameters in human vaccine studies.

Great promise of the mRNA vaccine platform comes from the ample possibilities to manipulate mRNA backbone, 5′ cap, UTRs, codon usage, nucleoside-base modifications, self-amplification, delivery systems, co-delivery of immunomodulatory molecules, safety, and manufacturing ease, which collectively provide enormous flexibility and room for optimization.[30,35,37,42,44,82] At this point of time, there are many more promises than human data and optimization of all these parameters in humans for each disease target may take some time.[42] For example, it was demonstrated for self-amplifying mRNA that potent initial induction of type I interferons by double-stranded RNA (dsRNA) was counterproductive to immunogen expression and subsequent immunogenicity.[83,84] Thus, any rational exploitation of the innate signals and/or immunomodulation will require careful evaluation, which will ultimately need to be confirmed in the target species, i.e., humans.[85] Nevertheless, new technologies can often precipitate the long-awaited breakthroughs in difficult challenges of public health, such as prevention and treatment of HIV-1, malaria, tuberculosis, cancer, and allergies, and the mRNA platform has the potential to be such a transforming technology. Many commercial programs have already started moving into clinic,[44] and first results are emerging. We argue that the tHIVconsvX conserved regions and their match to global pandemic HIV-1s is currently state of the art and that the specificity of T cells and their breadth we aim to induce has a real potential to hurt HIV-1 where it is most vulnerable. This is strongly supported by our studies in ART treatment-naive, HIV-1-infected patients in Japan, whereby both the magnitude and breadth of tHIVconsvX-specific responses induced in natural HIV-1 infection correlated directly with high CD4 T cell count and indirectly with low plasma viral load.[26,67] saRNA is now poised to accelerate the iterative process of identifying and optimizing induction of the necessary protective T cell traits controlling HIV-1 infection in human clinical trials.

MATERIALS AND METHODS

Preparation and Formulation of AIR.tHIVconsv1 and AIR.tHIVconsv2 saRNA Vaccines

The synthesis of the AIR.tHIVconsv1 and AIR.tHIVconsv2 saRNAs is based on T7 *in vitro* transcription protocols provided by MEGAscript T7 Transcription Kit (Thermo Fisher Scientific, Schwerte, Germany) described before.[83] Briefly, a linear DNA template was generated containing the Semliki Forest virus isolate L10 saRNA vector (accession

number AJ251359), and modifications were described elsewhere. The different tHIVconsvX sequences, including the co-transcriptional capping, with the synthetic cap analog γ-S-ARCA(D1). The *in vitro* transcription followed an already described process[86] and was optimized with respect to saRNA length of up to 10,000 nt. For *in vivo* application, saRNA was formulated with a polyethylenimine-based reagent (Polyplus Tranfection, Illkirch, France). The formulation protocol followed to the supplier's protocol and particle generation was confirmed by particle size measurement using a Dynamic Light Scattering instrument (Wyatt Technology, Dernbach, Germany). Only particles below 200 nm were released for *in vivo* application. All saRNA preparations were tested for their RNA integrity using a Bioanalyzer instrument internally optimized with respect to saRNA length (Agilent Technologies, Waldbronn, Germany). The preparation of other vaccines used in this study was described before.[26,27]

Mice and Immunizations and Preparation of Splenocytes

Six-week-old female BALB/c or CD1-SWISS mice were purchased from Envigo (UK) or Charles River Laboratories (UK), respectively, and housed at the Functional Genomics Facility, University of Oxford. Mice were immunized intramuscularly under general anesthesia either with varying amounts of saRNA as indicated in figures, a total 10^8 infectious units (IUs) of rChAdOx1s, and a total of 5×10^6 plaque-forming units (PFUs) of rMVAs. Immunization schedules are listed in Figure S1. On the day of sacrifice, spleens were collected and cells isolated by pressing organs individually through a 70-μm nylon mesh sterile cell strainer (Fisher Scientific) using a 5-mL syringe rubber plunger. Following the removal of red blood cells with RBC Lysing Buffer Hybri-Max (Sigma), splenocytes were washed and resuspended in R10 (RPMI 1640 supplemented with 10% fetal calf serum [FCS], penicillin and streptomycin, and β-mercaptoethanol) for ELISPOT, ICS assays, and other procedures. All procedures and care were approved by the local Clinical Medicine Ethical Review Committee, University of Oxford and conformed strictly to the United Kingdom Home Office Guidelines under the Animals (Scientific Procedures) Act 1986. Experiments were conducted under project license 30/3387 held by T.H.

Peptides and Peptide Pools

All peptides were at least 90% pure by mass spectrometry (Ana Spec, San Jose, CA, USA and Synpeptide, Shanghai, China), dissolved in DMSO (Sigma-Aldrich) to yield a stock of 10 mg/mL, and stored at −80°C. Four hundred and one tHIVconsvX-derived peptides 15-mer overlapping by 11 amino acids were divided into 10 pools P1–P10 of 34–47 individual peptides in a way that variant peptides were always present in the same pool for use in ICS and ELISPOT assays. Also peptides pairs AMQ (AMQMLKETI and AMQMLKDTI) and VLV (VLVGPTPVNI and VLIGPTPVNI), a pool of 17 pairs of stimulatory "BALB/c" peptides, were employed as specified in each figure. The peptides were used at a final concentration of 2 μg/mL.

The IFN-γ ELISPOT Assay

The ELISPOT assay was performed using the Mouse IFN-γ ELISpot kit (Mabtech) according to the manufacturer's instructions. Immune splenocytes were collected and tested separately from individual mice. Peptides were used at 2 μg/mL each, and splenocytes at 2×10^5 cells/well were added to 96-well high-protein-binding Immobilon-P membrane plates (Millipore) that had been precoated with 5 μg/mL anti-IFN-γ monoclonal antibody (mAb) AN18 (Mabtech, Stockholm, Sweden). The plates were incubated at 37°C in 5% CO_2 for 18 hr and washed with PBS before the addition of 1 μg/mL biotinylated anti-IFN-γ Mab (Mabtech) at room temperature for 2 hr. The plates were then washed with PBS, incubated with 1 μg/mL streptavidin-conjugated alkaline phosphatase (Mabtech) at room temperature for 1 hr, washed with PBS, and individual cytokine-producing units were detected as dark spots after a 10-min reaction with 5-bromo-4-chloro-3-idolyl phosphate and nitro blue tetrazolium using an alkaline-phosphatase-conjugate substrate (Bio-Rad, Richmond, CA, USA). Spot-forming units were counted using the AID ELISpot Reader System (Autoimmun Diagnostika). The frequencies of responding cells were expressed as a number of spot-forming units/10^6 splenocytes.

ICS Assay

Splenocytes or PBMCs isolated from whole blood were stimulated with peptide at 2 μg/mL; ionomycin and phorbol myristate acetate (PMA) at 2.0 μg/mL and 0.5 μg/mL, respectively; or tissue culture media with 1% DMSO as a negative control. The cultures were supplemented with anti-CD107a phycoerythrin (PE)-conjugated mAb (eBioscience). The cells were incubated at 37°C, 5% CO_2 for 2 hr prior to the addition of brefeldin A and monensin (BD Biosciences) and then left in culture overnight. The cells were centrifuged briefly, washed in PBS plus 5% BSA (Sigma-Aldrich), and the pellet resuspended in 40 μL of CD16/32 with LIVE/DEAD fixable aqua stain (Molecular Probes, Invitrogen). Cells were washed; a mastermix of anti-membrane marker mAbs was prepared containing CD4 allophycocyanin (APC)/Cy7 (BioLegend), CD3 PerCP-eFluor710, and CD8a eFluor 450 (both from eBioscience); and 40 μL added to each tube. The cells were incubated at 4°C for 30 min and then permeabilized using Fix/Perm solution (Becton Dickinson) for 20 min at 4°C. The cells were washed with Perm Wash buffer (Becton Dickinson), and a mastermix of anti-intracellular molecule mAbs was prepared containing IFN-γ PE-Cy7, IL-2 APC, and TNF-α fluorescein isothiocyanate (FITC) (all from eBioscience). The cells were incubated at 4°C for 30 min, washed, and resuspended in Perm Wash buffer prior to running on an LSRII flow cytometer (Becton Dickinson).

Memory Subtype Assay

Splenocytes and PBMCs isolated from whole blood were stimulated with specific tHIVconsvX-derived BALB/c peptide pool and stained with 100 μL of a mastermix of anti-membrane marker mAbs containing LIVE/DEAD fixable aqua stain (Molecular Probes, Invitrogen), CD3-APC, CD4-FITC, CD8a-eFluor 450, CD44-PE, and CD62-L-PE-Cy7 605 (all from eBioscience). The cells were incubated at 4°C for 30 min, washed, and fixed with 1% paraformaldehyde in PBS prior to running on an LSRII flow cytometer (Becton Dickinson). The frequencies of the subtypes in $CD8^+$ and $CD4^+$ T cells represent the differences in stimulated and unstimulated immune cells.

MHC Class I Tetramer Staining

Refolded MHC-peptide monomers (immunAware, Denmark) were tetramerized by adding streptavidin-conjugate-APC (Life Technologies) at 4°C. Splenocytes were stained with 30 μL of the optimal tetramer concentration for 20 min at room temperature and washed followed by the addition of 40 μL of a mastermix of anti-membrane marker mAbs containing LIVE/DEAD fixable aqua stain (Molecular Probes, Invitrogen), CD4 APC/Cy7 (BioLegend), CD3 PerCP-eFluor710, and CD8a eFluor 450 (both from eBioscience). The cells were incubated at 4°C for 30 min, washed, and fixed with 1% paraformaldehyde in PBS prior to running on an LSRII flow cytometer (Becton Dickinson).

In Vivo Killing Assay

Syngeneic splenocytes were incubated with or without 2 μg/mL peptides at 37°C, 5% CO_2 for 90 min and thoroughly washed. Unpulsed cells were labeled with 5-(and-6)-([(4-chloromethyl)benzoyl]amino)tetramethylrhodamine (CMTMR; Molecular Probes, Invitrogen) only, and peptide-pulsed cells were labeled with 750 nM (AMQMLKETI - 1 and VLVGPTPVNI - 3) and 150 nM (AMQMLKDTI - 2 and ILVGPTPVNI - 4) 5(6)-carboxyfluorescein diacetate N-succinimidyl ester (CFSE) (Molecular Probes; Invitrogen); in addition, the VLVGPTPVNI and ILVGPTPVNI peptide-pulsed cells were incubated with 10 μM CMTMR at 37°C for 15 min and a further 15 min in fresh medium. Five differentially labeled cell cultures were combined for intravenous adoptive transfer 5 weeks after saRNA and 1 week after rMVA immunizations, with each animal receiving approximately 2×10^6 cells of each population. Ten hours later, splenocytes were isolated and analyzed using flow cytometry. Cytolytic activity was estimated using the following formula: adjusted % survival = 100 × (% survival of peptide-pulsed cells/mean % survival of peptide unpulsed cells), followed by the calculation of % specific lysis = 100 − adjusted % survival.

CSFE Proliferation Assay

Cryopreserved splenocytes were thawed, resuspended in pre-warmed PBS with 0.1% BSA at a final concentration of 1×10^6 cells/mL, and labeled with 750 nM CFSE for 10 min at 37°C, 5% CO_2. The staining was quenched by adding 5 volumes of ice-cold R10 followed by a 5-min incubation on ice. The cells were pelleted, washed, and plated in 96-well round-bottom plates at a concentration of 1×10^6 cells/well. The CFSE-labeled cells were then stimulated for 5 days with 2 μg/mL of peptide, 2.0 μg/mL ionomycin, and 0.5 μg/mL PMA (positive control) or tissue culture media with 1% DMSO (negative control). The cells were stained with a mastermix containing the dead cell marker (LIVE/DEAD Fixable Aqua stain; Invitrogen) and anti-membrane marker mAbs anti-CD4-APC/Cy7 (BioLegend), anti-CD3-PerCPeFluor710, and anti-CD8-eFluor450 (both from eBioscience), fixed and acquired on a BD LSR II flow cytometer. Data analysis was performed using FlowJo software (Tree Star) with gating shown in Figure S6.

Statistical Analysis

Statistical analyses were performed using Graph Pad Prism version 7. ELISPOT and flow cytometry results were assumed to be non-Gaussian in distribution; thus, non-parametric tests were used throughout and medians (range) are shown. Multiple comparisons were performed using the Kruskal-Wallis test with Dunn's multiple comparison post test for nonparametric data. Groups with the same *in vitro* restimulations were compared using two-tailed Mann-Whitney U tests. Two-tailed p values were used, and p values of less than 0.05 were considered statistically significant.

SUPPLEMENTAL INFORMATION

Supplemental Information includes eight figures and can be found with this article online at https://doi.org/10.1016/j.omtm.2018.10.010.

AUTHOR CONTRIBUTIONS

N.M. designed and conducted the experiments and analyzed the data, A.B.V. planned and prepared the saRNA vaccines, S.B. predicted BALB/c epitopes and prepared tetramers, S.E. prepared the saRNA vaccines, U.S. supervised the study and experimental strategy, and T.H. designed the experiments, analyzed data, and wrote the manuscript with contribution from all authors.

CONFLICTS OF INTEREST

T.H. is one of the inventors on PCT Application No. PCT/US2014/058422 and EP14846993.5 concerning the tHIVconsvX immunogen. A.B.V., S.E., and U.S. are inventors on patents and/or patent applications that cover parts of this article. U.S. is co-founder and CEO of BioNTech AG (Mainz, Germany), a privately held biopharmaceutical company developing RNA-based vaccines and immunotherapeutics. A.B.V. and S.E. are employees of BioNTech RNA Pharmaceuticals GmbH. The other authors declare no conflicts of interest.

ACKNOWLEDGMENTS

We thank Jonathan Mottl for excellent technical assistance. The work is jointly funded by the Medical Research Council (MRC) UK and the UK Department for International Development (DFID) under the MRC/DFID Concordat agreements (MR/N023668/1) and European Union's Horizon 2020 research and innovation programme under grant agreement no. 681137; T.H. is the Jenner Institute Investigator. The funders had no role in study design, data collection and analysis, decision to publish, or preparation of the manuscript.

REFERENCES

1. UNAIDS (2015). AIDS by the numbers. UNAIDS, http://www.unaids.org/en/resources/documents/2016/AIDS-by-the-numbers.
2. Kibengo, F.M., Ruzagira, E., Katende, D., Bwanika, A.N., Bahemuka, U., Haberer, J.E., Bangsberg, D.R., Barin, B., Rooney, J.F., Mark, D., et al. (2013). Safety, adherence and acceptability of intermittent tenofovir/emtricitabine as HIV pre-exposure prophylaxis (PrEP) among HIV-uninfected Ugandan volunteers living in HIV-serodiscordant relationships: a randomized, clinical trial. PLoS ONE *8*, e74314.
3. Van der Elst, E.M., Mbogua, J., Operario, D., Mutua, G., Kuo, C., Mugo, P., Kanungi, J., Singh, S., Haberer, J., Priddy, F., and Sanders, E.J. (2013). High acceptability of HIV pre-exposure prophylaxis but challenges in adherence and use: qualitative insights from a phase I trial of intermittent and daily PrEP in at-risk populations in Kenya. AIDS Behav. *17*, 2162–2172.
4. Bonjoch, A., Figueras, M., Estany, C., Perez-Alvarez, N., Rosales, J., del Rio, L., di Gregorio, S., Puig, J., Gómez, G., Clotet, B., and Negredo, E.; Osteoporosis Study

Group (2010). High prevalence of and progression to low bone mineral density in HIV-infected patients: a longitudinal cohort study. AIDS *24*, 2827–2833.

5. Worm, S.W., Sabin, C., Weber, R., Reiss, P., El-Sadr, W., Dabis, F., De Wit, S., Law, M., Monforte, A.D., Friis-Møller, N., et al. (2010). Risk of myocardial infarction in patients with HIV infection exposed to specific individual antiretroviral drugs from the 3 major drug classes: the data collection on adverse events of anti-HIV drugs (D:A:D) study. J. Infect. Dis. *201*, 318–330.

6. Zimmermann, A.E., Pizzoferrato, T., Bedford, J., Morris, A., Hoffman, R., and Braden, G. (2006). Tenofovir-associated acute and chronic kidney disease: a case of multiple drug interactions. Clin. Infect. Dis. *42*, 283–290.

7. McMichael, A.J., and Haynes, B.F. (2012). Lessons learned from HIV-1 vaccine trials: new priorities and directions. Nat. Immunol. *13*, 423–427.

8. Haynes, B.F., Shaw, G.M., Korber, B., Kelsoe, G., Sodroski, J., Hahn, B.H., Borrow, P., and McMichael, A.J. (2016). HIV-host interactions: implications for vaccine design. Cell Host Microbe *19*, 292–303.

9. Hanke, T. (2014). Conserved immunogens in prime-boost strategies for the next-generation HIV-1 vaccines. Expert Opin. Biol. Ther. *14*, 601–616.

10. Létourneau, S., Im, E.-J., Mashishi, T., Brereton, C., Bridgeman, A., Yang, H., Dorrell, L., Dong, T., Korber, B., McMichael, A.J., and Hanke, T. (2007). Design and pre-clinical evaluation of a universal HIV-1 vaccine. PLoS ONE *2*, e984.

11. Abdul-Jawad, S., Ondondo, B., van Hateren, A., Gardner, A., Elliott, T., Korber, B., and Hanke, T. (2016). Increased valency of conserved-mosaic vaccines enhances the breadth and depth of epitope recognition. Mol. Ther. *24*, 375–384.

12. Borthwick, N.J., Rosario, M., Schiffner, T., Bowles, E., Ahmed, T., Liljeström, P., Stewart-Jones, G.E., Drijfhout, J.W., Melief, C.J., and Hanke, T. (2015). Humoral responses to HIVconsv induced by heterologous vaccine modalities in rhesus macaques. Immun. Inflamm. Dis. *3*, 82–93.

13. Clutton, G., Bridgeman, A., Reyes-Sandoval, A., Hanke, T., and Dorrell, L. (2015). Transient IL-10 receptor blockade can enhance CD8(+) T cell responses to a simian adenovirus-vectored HIV-1 conserved region immunogen. Hum. Vaccin. Immunother. *11*, 1030–1035.

14. Clutton, G., Carpov, A., Parks, C.L., Dean, H.J., Montefiori, D.C., and Hanke, T. (2014). Optimizing parallel induction of HIV type 1-specific antibody and T-cell responses by multicomponent subunit vaccines. AIDS *28*, 2495–2504.

15. Im, E.-J., Hong, J.P., Roshorm, Y., Bridgeman, A., Létourneau, S., Liljeström, P., Potash, M.J., Volsky, D.J., McMichael, A.J., and Hanke, T. (2011). Protective efficacy of serially up-ranked subdominant CD8+ T cell epitopes against virus challenges. PLoS Pathog. *7*, e1002041.

16. Knudsen, M.L., Mbewe-Mvula, A., Rosario, M., Johansson, D.X., Kakoulidou, M., Bridgeman, A., Reyes-Sandoval, A., Nicosia, A., Ljungberg, K., Hanke, T., and Liljeström, P. (2012). Superior induction of T cell responses to conserved HIV-1 regions by electroporated alphavirus replicon DNA compared to that with conventional plasmid DNA vaccine. J. Virol. *86*, 4082–4090.

17. Ondondo, B., Abdul-Jawad, S., Bridgeman, A., and Hanke, T. (2014). Characterization of T-cell responses to conserved regions of the HIV-1 proteome in BALB/c mice. Clin. Vaccine Immunol. *21*, 1565–1572.

18. Ondondo, B., Brennan, C., Nicosia, A., Crome, S.J., and Hanke, T. (2013). Absence of systemic toxicity changes following intramuscular administration of novel pSG2.HIVconsv DNA, ChAdV63.HIVconsv and MVA.HIVconsv vaccines to BALB/c mice. Vaccine *31*, 5594–5601.

19. Rosario, M., Bridgeman, A., Quakkelaar, E.D., Quigley, M.F., Hill, B.J., Knudsen, M.L., Ammendola, V., Ljungberg, K., Borthwick, N., Im, E.J., et al. (2010). Long peptides induce polyfunctional T cells against conserved regions of HIV-1 with superior breadth to single-gene vaccines in macaques. Eur. J. Immunol. *40*, 1973–1984.

20. Borthwick, N., Ahmed, T., Ondondo, B., Hayes, P., Rose, A., Ebrahimsa, U., Hayton, E.J., Black, A., Bridgeman, A., Rosario, M., et al. (2014). Vaccine-elicited human T cells recognizing conserved protein regions inhibit HIV-1. Mol. Ther. *22*, 464–475.

21. Borthwick, N., Lin, Z., Akahoshi, T., Llano, A., Silva-Arrieta, S., Ahmed, T., Dorrell, L., Brander, C., Murakoshi, H., Takiguchi, M., and Hanke, T. (2017). Novel, in-natural-infection subdominant HIV-1 CD8+ T-cell epitopes revealed in human recipients of conserved-region T-cell vaccines. PLoS ONE *12*, e0176418.

22. Hancock, G., Morón-López, S., Kopycinski, J., Puertas, M.C., Giannoulatou, E., Rose, A., Salgado, M., Hayton, E.J., Crook, A., Morgan, C., et al. (2017). Evaluation of the immunogenicity and impact on the latent HIV-1 reservoir of a conserved region vaccine, MVA.HIVconsv, in antiretroviral therapy-treated subjects. J. Int. AIDS Soc. *20*, 21171.

23. Hancock, G., Yang, H., Yorke, E., Wainwright, E., Bourne, V., Frisbee, A., Payne, T.L., Berrong, M., Ferrari, G., Chopera, D., et al. (2015). Identification of effective subdominant anti-HIV-1 CD8+ T cells within entire post-infection and post-vaccination immune responses. PLoS Pathog. *11*, e1004658.

24. Hayton, E.J., Rose, A., Ibrahimsa, U., Del Sorbo, M., Capone, S., Crook, A., Black, A.P., Dorrell, L., and Hanke, T. (2014). Safety and tolerability of conserved region vaccines vectored by plasmid DNA, simian adenovirus and modified vaccinia virus ankara administered to human immunodeficiency virus type 1-uninfected adults in a randomized, single-blind phase I trial. PLoS ONE *9*, e101591.

25. Mutua, G., Farah, B., Langat, R., Indangasi, J., Ogola, S., Onsembe, B., Kopycinski, J.T., Hayes, P., Borthwick, N.J., Ashraf, A., et al. (2016). Broad HIV-1 inhibition in vitro by vaccine-elicited CD8(+) T cells in African adults. Mol. Ther. Methods Clin. Dev. *3*, 16061.

26. Ondondo, B., Murakoshi, H., Clutton, G., Abdul-Jawad, S., Wee, E.G., Gatanaga, H., Oka, S., McMichael, A.J., Takiguchi, M., Korber, B., and Hanke, T. (2016). Novel conserved-region T-cell mosaic vaccine with high global HIV-1 coverage is recognized by protective responses in untreated infection. Mol. Ther. *24*, 832–842.

27. Wee, E.G., Ondondo, B., Berglund, P., Archer, J., McMichael, A.J., Baltimore, D., Ter Meulen, J.H., and Hanke, T. (2017). HIV-1 conserved mosaics delivered by regimens with integration-deficient DC-targeting lentiviral vector induce robust T cells. Mol. Ther. *25*, 494–503.

28. Pichlmair, A., and Reis e Sousa, C. (2007). Innate recognition of viruses. Immunity *27*, 370–383.

29. Pollard, C., Rejman, J., De Haes, W., Verrier, B., Van Gulck, E., Naessens, T., De Smedt, S., Bogaert, P., Grooten, J., Vanham, G., and De Koker, S. (2013). Type I IFN counteracts the induction of antigen-specific immune responses by lipid-based delivery of mRNA vaccines. Mol. Ther. *21*, 251–259.

30. Holtkamp, S., Kreiter, S., Selmi, A., Simon, P., Koslowski, M., Huber, C., Türeci, O., and Sahin, U. (2006). Modification of antigen-encoding RNA increases stability, translational efficacy, and T-cell stimulatory capacity of dendritic cells. Blood *108*, 4009–4017.

31. Karikó, K., Buckstein, M., Ni, H., and Weissman, D. (2005). Suppression of RNA recognition by Toll-like receptors: the impact of nucleoside modification and the evolutionary origin of RNA. Immunity *23*, 165–175.

32. Karikó, K., Muramatsu, H., Welsh, F.A., Ludwig, J., Kato, H., Akira, S., and Weissman, D. (2008). Incorporation of pseudouridine into mRNA yields superior nonimmunogenic vector with increased translational capacity and biological stability. Mol. Ther. *16*, 1833–1840.

33. Ricci, E.P., Kucukural, A., Cenik, C., Mercier, B.C., Singh, G., Heyer, E.E., Ashar-Patel, A., Peng, L., and Moore, M.J. (2014). Staufen1 senses overall transcript secondary structure to regulate translation. Nat. Struct. Mol. Biol. *21*, 26–35.

34. Sahin, U., Karikó, K., and Türeci, Ö. (2014). mRNA-based therapeutics–developing a new class of drugs. Nat. Rev. Drug Discov. *13*, 759–780.

35. Strenkowska, M., Grzela, R., Majewski, M., Wnek, K., Kowalska, J., Lukaszewicz, M., Zuberek, J., Darzynkiewicz, E., Kuhn, A.N., Sahin, U., and Jemielity, J. (2016). Cap analogs modified with 1,2-dithiodiphosphate moiety protect mRNA from decapping and enhance its translational potential. Nucleic Acids Res. *44*, 9578–9590.

36. Warren, L., Manos, P.D., Ahfeldt, T., Loh, Y.H., Li, H., Lau, F., Ebina, W., Mandal, P.K., Smith, Z.D., Meissner, A., et al. (2010). Highly efficient reprogramming to pluripotency and directed differentiation of human cells with synthetic modified mRNA. Cell Stem Cell *7*, 618–630.

37. Pardi, N., Hogan, M.J., Pelc, R.S., Muramatsu, H., Andersen, H., DeMaso, C.R., Dowd, K.A., Sutherland, L.L., Scearce, R.M., Parks, R., et al. (2017). Zika virus protection by a single low-dose nucleoside-modified mRNA vaccination. Nature *543*, 248–251.

38. Brito, L.A., Chan, M., Shaw, C.A., Hekele, A., Carsillo, T., Schaefer, M., Archer, J., Seubert, A., Otten, G.R., Beard, C.W., et al. (2014). A cationic nanoemulsion for the delivery of next-generation RNA vaccines. Mol. Ther. *22*, 2118–2129.

39. Chahal, J.S., Khan, O.F., Cooper, C.L., McPartlan, J.S., Tsosie, J.K., Tilley, L.D., Sidik, S.M., Lourido, S., Langer, R., Bavari, S., et al. (2016). Dendrimer-RNA nanoparticles generate protective immunity against lethal Ebola, H1N1 influenza, and Toxoplasma gondii challenges with a single dose. Proc. Natl. Acad. Sci. USA *113*, E4133–E4142.

40. Kranz, L.M., Diken, M., Haas, H., Kreiter, S., Loquai, C., Reuter, K.C., Meng, M., Fritz, D., Vascotto, F., Hefesha, H., et al. (2016). Systemic RNA delivery to dendritic cells exploits antiviral defence for cancer immunotherapy. Nature *534*, 396–401.

41. Weide, B., Pascolo, S., Scheel, B., Derhovanessian, E., Pflugfelder, A., Eigentler, T.K., Pawelec, G., Hoerr, I., Rammensee, H.G., and Garbe, C. (2009). Direct injection of protamine-protected mRNA: results of a phase 1/2 vaccination trial in metastatic melanoma patients. J. Immunother. *32*, 498–507.

42. DeFrancesco, L. (2017). The 'anti-hype' vaccine. Nat. Biotechnol. *35*, 193–197.

43. Schnee, M., Vogel, A.B., Voss, D., Petsch, B., Baumhof, P., Kramps, T., and Stitz, L. (2016). An mRNA vaccine encoding rabies virus glycoprotein induces protection against lethal infection in mice and correlates of protection in adult and newborn pigs. PLoS Negl. Trop. Dis. *10*, e0004746.

44. Kaczmarek, J.C., Kowalski, P.S., and Anderson, D.G. (2017). Advances in the delivery of RNA therapeutics: from concept to clinical reality. Genome Med. *9*, 60.

45. Zhou, X., Berglund, P., Rhodes, G., Parker, S.E., Jondal, M., and Liljeström, P. (1994). Self-replicating Semliki Forest virus RNA as recombinant vaccine. Vaccine *12*, 1510–1514.

46. Betts, M.R., Brenchley, J.M., Price, D.A., De Rosa, S.C., Douek, D.C., Roederer, M., and Koup, R.A. (2003). Sensitive and viable identification of antigen-specific CD8+ T cells by a flow cytometric assay for degranulation. J. Immunol. Methods *281*, 65–78.

47. Rubio, V., Stuge, T.B., Singh, N., Betts, M.R., Weber, J.S., Roederer, M., and Lee, P.P. (2003). Ex vivo identification, isolation and analysis of tumor-cytolytic T cells. Nat. Med. *9*, 1377–1382.

48. Aggarwal, B.B. (2003). Signalling pathways of the TNF superfamily: a double-edged sword. Nat. Rev. Immunol. *3*, 745–756.

49. Kull, F.C., Jr. (1988). The TNF receptor in TNF-mediated cytotoxicity. Nat. Immun. Cell Growth Regul. *7*, 254–265.

50. Lazdins, J.K., Grell, M., Walker, M.R., Woods-Cook, K., Scheurich, P., and Pfizenmaier, K. (1997). Membrane tumor necrosis factor (TNF) induced cooperative signaling of TNFR60 and TNFR80 favors induction of cell death rather than virus production in HIV-infected T cells. J. Exp. Med. *185*, 81–90.

51. Baden, L.R., Karita, E., Mutua, G., Bekker, L.G., Gray, G., Page-Shipp, L., Walsh, S.R., Nyombayire, J., Anzala, O., Roux, S., et al.; B003-IPCAVD004-HVTN091 Study Group (2016). Assessment of the safety and immunogenicity of 2 novel vaccine platforms for HIV-1 prevention: a randomized trial. Ann. Intern. Med. *164*, 313–322.

52. Vogel, A.B., Lambert, L., Kinnear, E., Busse, D., Erbar, S., Reuter, K.C., Wicke, L., Perkovic, M., Beissert, T., Haas, H., et al. (2018). Self-amplifying RNA vaccines give equivalent protection against influenza to mRNA vaccines but at much lower doses. Mol. Ther. *26*, 446–455.

53. Borrow, P., Lewicki, H., Hahn, B.H., Shaw, G.M., and Oldstone, M.B. (1994). Virus-specific CD8+ cytotoxic T-lymphocyte activity associated with control of viremia in primary human immunodeficiency virus type 1 infection. J. Virol. *68*, 6103–6110.

54. Fellay, J., Shianna, K.V., Ge, D., Colombo, S., Ledergerber, B., Weale, M., Zhang, K., Gumbs, C., Castagna, A., Cossarizza, A., et al. (2007). A whole-genome association study of major determinants for host control of HIV-1. Science *317*, 944–947.

55. Goonetilleke, N., Liu, M.K., Salazar-Gonzalez, J.F., Ferrari, G., Giorgi, E., Ganusov, V.V., Keele, B.F., Learn, G.H., Turnbull, E.L., Salazar, M.G., et al.; CHAVI Clinical Core B (2009). The first T cell response to transmitted/founder virus contributes to the control of acute viremia in HIV-1 infection. J. Exp. Med. *206*, 1253–1272.

56. Goulder, P.J., Brander, C., Tang, Y., Tremblay, C., Colbert, R.A., Addo, M.M., Rosenberg, E.S., Nguyen, T., Allen, R., Trocha, A., et al. (2001). Evolution and transmission of stable CTL escape mutations in HIV infection. Nature *412*, 334–338.

57. Koup, R.A., Safrit, J.T., Cao, Y., Andrews, C.A., McLeod, G., Borkowsky, W., Farthing, C., and Ho, D.D. (1994). Temporal association of cellular immune responses with the initial control of viremia in primary human immunodeficiency virus type 1 syndrome. J. Virol. *68*, 4650–4655.

58. Ogg, G.S., Jin, X., Bonhoeffer, S., Dunbar, P.R., Nowak, M.A., Monard, S., Segal, J.P., Cao, Y., Rowland-Jones, S.L., Cerundolo, V., et al. (1998). Quantitation of HIV-1-specific cytotoxic T lymphocytes and plasma load of viral RNA. Science *279*, 2103–2106.

59. Carrington, M., and O'Brien, S.J. (2003). The influence of HLA genotype on AIDS. Annu. Rev. Med. *54*, 535–551.

60. Dalmasso, C., Carpentier, W., Meyer, L., Rouzioux, C., Goujard, C., Chaix, M.L., Lambotte, O., Avettand-Fenoel, V., Le Clerc, S., de Senneville, L.D., et al.; ANRS Genome Wide Association 01 (2008). Distinct genetic loci control plasma HIV-RNA and cellular HIV-DNA levels in HIV-1 infection: the ANRS Genome Wide Association 01 study. PLoS ONE *3*, e3907.

61. Salazar-Gonzalez, J.F., Salazar, M.G., Keele, B.F., Learn, G.H., Giorgi, E.E., Li, H., Decker, J.M., Wang, S., Baalwa, J., Kraus, M.H., et al. (2009). Genetic identity, biological phenotype, and evolutionary pathways of transmitted/founder viruses in acute and early HIV-1 infection. J. Exp. Med. *206*, 1273–1289.

62. Carrington, M., Nelson, G.W., Martin, M.P., Kissner, T., Vlahov, D., Goedert, J.J., Kaslow, R., Buchbinder, S., Hoots, K., and O'Brien, S.J. (1999). HLA and HIV-1: heterozygote advantage and B*35-Cw*04 disadvantage. Science *283*, 1748–1752.

63. Goulder, P.J., and Walker, B.D. (2012). HIV and HLA class I: an evolving relationship. Immunity *37*, 426–440.

64. Goulder, P.J.R., Phillips, R.E., Colbert, R.A., McAdam, S., Ogg, G., Nowak, M.A., Giangrande, P., Luzzi, G., Morgan, B., Edwards, A., et al. (1997). Late escape from an immunodominant cytotoxic T-lymphocyte response associated with progression to AIDS. Nat. Med. *3*, 212–217.

65. Mothe, B., Llano, A., Ibarrondo, J., Daniels, M., Miranda, C., Zamarreño, J., Bach, V., Zuniga, R., Pérez-Álvarez, S., Berger, C.T., et al. (2011). Definition of the viral targets of protective HIV-1-specific T cell responses. J. Transl. Med. *9*, 208.

66. Murakoshi, H., Akahoshi, T., Koyanagi, M., Chikata, T., Naruto, T., Maruyama, R., Tamura, Y., Ishizuka, N., Gatanaga, H., Oka, S., and Takiguchi, M. (2015). Clinical control of HIV-1 by cytotoxic T cells specific for multiple conserved epitopes. J. Virol. *89*, 5330–5339.

67. Murakoshi, H., Zou, C., Kuse, N., Akahoshi, T., Chikata, T., Gatanaga, H., Oka, S., Hanke, T., and Takiguchi, M. (2018). $CD8^+$ T cells specific for conserved, cross-reactive Gag epitopes with strong ability to suppress HIV-1 replication. Retrovirology *15*, 46.

68. Matano, T., Shibata, R., Siemon, C., Connors, M., Lane, H.C., and Martin, M.A. (1998). Administration of an anti-CD8 monoclonal antibody interferes with the clearance of chimeric simian/human immunodeficiency virus during primary infections of rhesus macaques. J. Virol. *72*, 164–169.

69. Schmitz, J.E., Kuroda, M.J., Santra, S., Sasseville, V.G., Simon, M.A., Lifton, M.A., Racz, P., Tenner-Racz, K., Dalesandro, M., Scallon, B.J., et al. (1999). Control of viremia in simian immunodeficiency virus infection by CD8+ lymphocytes. Science *283*, 857–860.

70. Hansen, S.G., Ford, J.C., Lewis, M.S., Ventura, A.B., Hughes, C.M., Coyne-Johnson, L., Whizin, N., Oswald, K., Shoemaker, R., Swanson, T., et al. (2011). Profound early control of highly pathogenic SIV by an effector memory T-cell vaccine. Nature *473*, 523–527.

71. Hansen, S.G., Sacha, J.B., Hughes, C.M., Ford, J.C., Burwitz, B.J., Scholz, I., Gilbride, R.M., Lewis, M.S., Gilliam, A.N., Ventura, A.B., et al. (2013). Cytomegalovirus vectors violate CD8+ T cell epitope recognition paradigms. Science *340*, 1237874.

72. Hansen, S.G., Vieville, C., Whizin, N., Coyne-Johnson, L., Siess, D.C., Drummond, D.D., Legasse, A.W., Axthelm, M.K., Oswald, K., Trubey, C.M., et al. (2009). Effector memory T cell responses are associated with protection of rhesus monkeys from mucosal simian immunodeficiency virus challenge. Nat. Med. *15*, 293–299.

73. Hansen, S.G., Wu, H.L., Burwitz, B.J., Hughes, C.M., Hammond, K.B., Ventura, A.B., Reed, J.S., Gilbride, R.M., Ainslie, E., Morrow, D.W., et al. (2016). Broadly targeted $CD8^+$ T cell responses restricted by major histocompatibility complex E. Science *351*, 714–720.

74. Hansen, S.G., Piatak, M., Jr., Ventura, A.B., Hughes, C.M., Gilbride, R.M., Ford, J.C., Oswald, K., Shoemaker, R., Li, Y., Lewis, M.S., et al. (2013). Immune clearance of highly pathogenic SIV infection. Nature *502*, 100–104.

75. Kiepiela, P., Ngumbela, K., Thobakgale, C., Ramduth, D., Honeyborne, I., Moodley, E., Reddy, S., de Pierres, C., Mncube, Z., Mkhwanazi, N., et al. (2007). CD8+ T-cell

responses to different HIV proteins have discordant associations with viral load. Nat. Med. *13*, 46–53.

76. Rolland, M., Heckerman, D., Deng, W., Rousseau, C.M., Coovadia, H., Bishop, K., Goulder, P.J., Walker, B.D., Brander, C., and Mullins, J.I. (2008). Broad and Gag-biased HIV-1 epitope repertoires are associated with lower viral loads. PLoS ONE *3*, e1424.

77. Lee, J.K., Stewart-Jones, G., Dong, T., Harlos, K., Di Gleria, K., Dorrell, L., Douek, D.C., van der Merwe, P.A., Jones, E.Y., and McMichael, A.J. (2004). T cell cross-reactivity and conformational changes during TCR engagement. J. Exp. Med. *200*, 1455–1466.

78. Almeida, J.R., Price, D.A., Papagno, L., Arkoub, Z.A., Sauce, D., Bornstein, E., Asher, T.E., Samri, A., Schnuriger, A., Theodorou, I., et al. (2007). Superior control of HIV-1 replication by CD8+ T cells is reflected by their avidity, polyfunctionality, and clonal turnover. J. Exp. Med. *204*, 2473–2485.

79. Migueles, S.A., Laborico, A.C., Shupert, W.L., Sabbaghian, M.S., Rabin, R., Hallahan, C.W., Van Baarle, D., Kostense, S., Miedema, F., McLaughlin, M., et al. (2002). HIV-specific CD8+ T cell proliferation is coupled to perforin expression and is maintained in nonprogressors. Nat. Immunol. *3*, 1061–1068.

80. Betts, M.R., Nason, M.C., West, S.M., De Rosa, S.C., Migueles, S.A., Abraham, J., Lederman, M.M., Benito, J.M., Goepfert, P.A., Connors, M., et al. (2006). HIV nonprogressors preferentially maintain highly functional HIV-specific CD8+ T cells. Blood *107*, 4781–4789.

81. Yang, H., Wu, H., Hancock, G., Clutton, G., Sande, N., Xu, X., Yan, H., Huang, X., Angus, B., Kuldanek, K., et al. (2012). Antiviral inhibitory capacity of CD8+ T cells predicts the rate of CD4+ T-cell decline in HIV-1 infection. J. Infect. Dis. *206*, 552–561.

82. Kreiter, S., Selmi, A., Diken, M., Sebastian, M., Osterloh, P., Schild, H., Huber, C., Türeci, O., and Sahin, U. (2008). Increased antigen presentation efficiency by coupling antigens to MHC class I trafficking signals. J. Immunol. *180*, 309–318.

83. Beissert, T., Koste, L., Perkovic, M., Walzer, K.C., Erbar, S., Selmi, A., Diken, M., Kreiter, S., Türeci, Ö., and Sahin, U. (2017). Improvement of in vivo expression of genes delivered by self-amplifying RNA using Vaccinia virus immune evasion proteins. Hum. Gene Ther. *28*, 1138–1146.

84. Pepini, T., Pulichino, A.M., Carsillo, T., Carlson, A.L., Sari-Sarraf, F., Ramsauer, K., Debasitis, J.C., Maruggi, G., Otten, G.R., Geall, A.J., et al. (2017). Induction of an IFN-mediated antiviral response by a self-amplifying RNA vaccine: implications for vaccine design. J. Immunol. *198*, 4012–4024.

85. Suntharalingam, G., Perry, M.R., Ward, S., Brett, S.J., Castello-Cortes, A., Brunner, M.D., and Panoskaltsis, N. (2006). Cytokine storm in a phase 1 trial of the anti-CD28 monoclonal antibody TGN1412. N. Engl. J. Med. *355*, 1018–1028.

86. Kuhn, A.N., Diken, M., Kreiter, S., Selmi, A., Kowalska, J., Jemielity, J., Darzynkiewicz, E., Huber, C., Türeci, O., and Sahin, U. (2010). Phosphorothioate cap analogs increase stability and translational efficiency of RNA vaccines in immature dendritic cells and induce superior immune responses in vivo. Gene Ther. *17*, 961–971.

OMTM, Volume *12*

Supplemental Information

Efficient Induction of T Cells against Conserved HIV-1 Regions by Mosaic Vaccines Delivered as Self-Amplifying mRNA

Nathifa Moyo, Annette B. Vogel, Søren Buus, Stephanie Erbar, Edmund G. Wee, Ugur Sahin, and Tomáš Hanke

Figure 2**a** – Dose response in BALB/c mice

Group	n	Regimen	Dose	Week 0	Week 5
1	3	R1	5 µg	AIR.tHIVconsv1	Cull
2	3	R1	1 µg	AIR.tHIVconsv1	Cull
3	3	R1	200 ng	AIR.tHIVconsv1	Cull
4	3	R1	40 ng	AIR.tHIVconsv1	Cull
5	3	R2	5 µg	AIR.tHIVconsv2	Cull
6	3	R2	1 µg	AIR.tHIVconsv2	Cull
7	3	R2	200 ng	AIR.tHIVconsv2	Cull
8	3	R2	40 ng	AIR.tHIVconsv2	Cull
9	3	R	2.5 µg + 2.5 µg	AIR.tHIVconsv1 + AIR.tHIVconsv2	Cull
10	3	R	0.5 µg + 0.5 µg	AIR.tHIVconsv1 + AIR.tHIVconsv2	Cull
11	3	R	100 ng + 100 ng	AIR.tHIVconsv1 + AIR.tHIVconsv2	Cull
12	3	R	20 ng + 20 ng	AIR.tHIVconsv1 + AIR.tHIVconsv2	Cull

Figure 2**b** – Adjuvantation in BALB/c mice

Groups	n	Regimen	Week 0	Week 2 or Week 5
1 & 4	2x3	R	AIR.tHIVconsv1 + AIR.tHIVconsv2 Polymer	Cull
2 & 5	2x3	R	AIR.tHIVconsv1 + AIR.tHIVconsv2	Cull
3 & 6	2x3	-	Polymer	Cull

Figure 3a and **c** – Kinetics of responses up to 5 weeks in BALB/c mice

Groups	n	Regimen	Week 0	Week 1 – Week 5 Weekly
1 - 5	5x5	R1	AIR.tHIVconsv1	Cull
6 - 10	5x5	R2	AIR.tHIVconsv2	Cull
11 - 15	5x5	R	AIR.tHIVconsv1 + AIR.tHIVconsv2	Cull

Figure 3**b** and 4 - Extended kinetics of responses up to 22 weeks in BALB/c mice

Groups	n	Regimen	Week 0	Week 6 - Week 22 Biweekly
1 - 9	9x5	R	AIR.tHIVconsv1 + AIR.tHIVconsv2	Cull

Figure 5 - Optimizing the time of homologous boost in BALB/c mice

Groups	n	Reg[a]	Week 0	Week 2	Week 4	Week 6	Week 10/14/18
1 & 4	3x5	R2R	-	-	AIR.tHIVconsv1 + AIR.tHIVconsv2	AIR.tHIVconsv1 + AIR.tHIVconsv2	Cull
2 & 5	3x5	R4R	-	AIR.tHIVconsv1 + AIR.tHIVconsv2	-	AIR.tHIVconsv1 + AIR.tHIVconsv2	Cull
3 & 6	3x5	R6R	AIR.tHIVconsv1 + AIR.tHIVconsv2	-	-	AIR.tHIVconsv1 + AIR.tHIVconsv2	Cull

Figure 6**a** Left & Middle – Heterologous regimens in BALB/c mice

Group	n	Reg	Week 0	Week 1	Week 2	Week 5	Week 6	Week 7
1	4	R	-	-	R	-	-	Cull
2	4	C	-	-	-	C	-	Cull
3	4	M	-	-	-	-	M	Cull
4	4	RR	R	-	R	-	-	Cull
5	4	RM	-	R	-	-	M	Cull
6	4	RC	R	-	-	C	-	Cull

Figure 6**a** Right– Heterologous regimens in BALB/c mice

Group	n	Reg	Week 0	Week 4	Week 6	Week 7
1	5	R	-	AIR.tHIVconsv1 + AIR.tHIVconsv2	-	Cull
2	5	RR	AIR.tHIVconsv1 + AIR.tHIVconsv2	AIR.tHIVconsv1 + AIR.tHIVconsv2	-	Cull
3	5	RM	-	AIR.tHIVconsv1 + AIR.tHIVconsv2	MVA.tHIVconsv3 + MVA.tHIVconsv4	Cull
4	5	RC	-	AIR.tHIVconsv1 + AIR.tHIVconsv2	ChAdOx1.tHIVconsv5 + ChAdOx1.tHIVconsv6	Cull

Figure 6**b** – $CD8^+$ T-cell peptide mapping in BALB/c mice

Group	n	Reg	Week 0	Week 4	Week 5
1	5	RM	AIR.tHIVconsv1 + AIR.tHIVconsv2	MVA.tHIVconsv3 + MVA.tHIVconsv4	Cull

Figure 6**c** and **d** – $CD8^+$ T-cell peptide mapping in BALB/c mice

Group	n	Reg	Week 0	Week 4	Week 5
1	4	RM	AIR.tHIVconsv1 + AIR.tHIVconsv2	MVA.tHIVconsv3 + MVA.tHIVconsv4	Cull

Figure 6**e** – In vivo killing in BALB/c mice

Group	n	Reg	Week 0	Week 2	Week 4	Week 5
1	3	R	AIR.tHIVconsv1 + AIR.tHIVconsv2	-	-	Cull
2	3	RM	-	AIR.tHIVconsv1 + AIR.tHIVconsv2	MVA.tHIVconsv3 + MVA.tHIVconsv4	Cull

Figure 7 Heterologous regimens in outbred CD1-SWISS mice

Group	n	Reg	Week 0	Week 4	Week 5	Week 7	Week 8
1	5[b]	RR	AIR.tHIVconsv1 + AIR.tHIVconsv2	AIR.tHIVconsv1 + AIR.tHIVconsv2	-	-	Cull
2	7	RM	-	-	AIR.tHIVconsv1 + AIR.tHIVconsv2	MVA.tHIVconsv3 + MVA.tHIVconsv4	Cull

Figure S1 Immunization regimens. Mice were immunized using the indicated regimens. All vaccines were delivered intramuscularly. R - 2.5 µg of AIR.tHIVconsv1 RNA + 2.5 µg of AIR.tHIVconsv2 RNA formulated; M – 2.5 x 10^6 PFU of MVA.tHIVconsv3 + 2.5 x 10^6 PFU of MVA.tHIVconsv4; C - 5 x 10^7 IU of ChAdOx1.tHIVconsv5 + 5 x 10^7 IU of ChAdOx1.tHIVconsv6. a – In R2R, R4R and R6R, the number indicates the gap in weeks between prime and boost administrations. b - 2 out of 7 mice died of vaccine-unrelated causes.

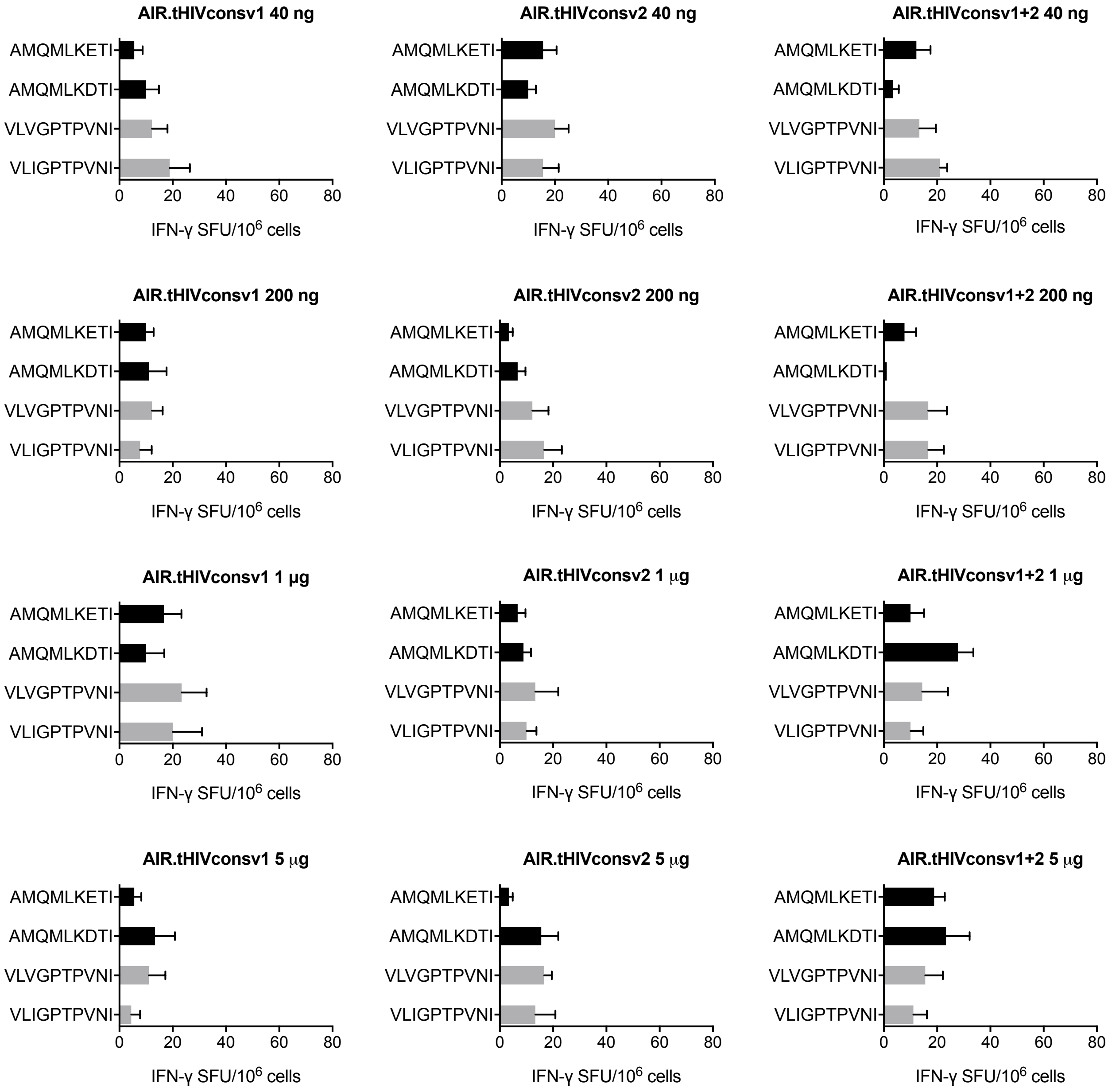

Figure S2 Dose response to saRNA vaccine at 1 week after vaccination. Groups of BALB/c mice were immunized with increasing doses of saRNA vaccine as indicated above the graphs, sacrificed 1 week later and their splenocytes were assessed in an IFN-γ ELISPOT assay using the two variants of the two most immunodominant epitopes for the vaccine-elicited frequencies of specific $CD8^+$ T cells. Data are shown as mean ± SD (n = 3).

**Paired peptide IDs**	**Variant IDs**	**Sequences**	
1	C001	PIVQNLQGQMVHQAI	**POOL 1**
	C002	PIVQNAQGQMVHQAL	**44 peptides**
2	C003	NLQGQMVHQAISPRT	
	C004	NAQGQMVHQALSPRT	
3	C005	QMVHQAISPRTLNAW	
	C006	QMVHQALSPRTLNAW	
4	C007	QAISPRTLNAWVKVI	
	C008	QALSPRTLNAWVKVV	
5	C009	PRTLNAWVKVIEEKA	
	C010	PRTLNAWVKVVEEKA	
6	C011	NAWVKVIEEKAFSPE	
	C012	NAWVKVVEEKAFSPE	
7	C013	KVIEEKAFSPEVIPM	
	C014	KVVEEKAFSPEVIPM	
8	C015	EKAFSPEVIPMFTAL	
	C016	EKAFSPEVIPMFSAL	
9	C017	SPEVIPMFTALSEGA	
	C018	SPEVIPMFSALSEGA	
10	C019	IPMFTALSEGATPQD	
	C020	IPMFSALSEGATPQD	
11	C021	TALSEGATPQDLNTM	
	C022	SALSEGATPQDLNMM	
12	C023	EGATPQDLNTMLNTV	
	C024	EGATPQDLNMMLNIV	
13	C025	PQDLNTMLNTVGGHQ	
	C026	PQDLNMMLNIVGGHQ	
14	C027	NTMLNTVGGHQAAMQ	
	C028	NMMLNIVGGHQAAMQ	
15	C029	NTVGGHQAAMQMLKD	
	C030	NIVGGHQAAMQMLKE	
16	C031	GHQAAMQMLKDTINE	
	C032	GHQAAMQMLKETINE	
17	C033	AMQMLKDTINEEAAE	
	C034	AMQMLKETINEEAAE	
18	C035	LKDTINEEAAEWDRV	
	C036	LKETINEEAAEWDRL	
19	C037	INEEAAEWDRVHPVH	
	C038	INEEAAEWDRLHPVH	
20	C039	AAEWDRVHPVHAGPI	
	C040	AAEWDRLHPVHAGPI	
21	C041	DRVHPVHAGPIAPGQ	
	C042	DRLHPVHAGPIPPGQ	
22	C043	PVHAGPIAPGQMREP	
	C044	PVHAGPIPPGQMREP	
23	C045	GPIAPGQMREPRGSD	**POOL 2**
	C046	GPIPPGQMREPRGSD	**47 peptides**
24	C047	PGQMREPRGSDIAGT	
25	C048	REPRGSDIAGTTSNL	

	C049	REPRGSDIAGTTSTL	
26	C050	GSDIAGTTSNLQEQI	
	C051	GSDIAGTTSTLQEQI	
27	C052	AGTTSNLQEQIGWMT	
	C053	AGTTSTLQEQIGWMT	
28	C054	SNLQEQIGWMTSNPP	
	C055	STLQEQIGWMTNNPP	
29	C056	EQIGWMTSNPPIPVG	
	C057	EQIGWMTNNPPIPVG	
30	C058	WMTSNPPIPVGDIYK	
	C059	WMTNNPPIPVGEIYK	
31	C060	NPPIPVGDIYKRWII	
	C061	NPPIPVGEIYKRWII	
32	C062	PVGDIYKRWIILGLN	
	C063	PVGEIYKRWIIMGLN	
33	C064	IYKRWIILGLNKIVR	
	C065	IYKRWIIMGLNKIVR	
34	C066	WIILGLNKIVRMYSP	
	C067	WIIMGLNKIVRMYSP	
35	C068	GLNKIVRMYSPVSIL	
	C069	GLNKIVRMYSPTSIL	
36	C070	IVRMYSPVSILDIRQ	
	C071	IVRMYSPTSILDIKQ	
37	C072	YSPVSILDIRQGPKE	
	C073	YSPTSILDIKQGPKE	
38	C074	SILDIRQGPKEPFRD	
	C075	SILDIKQGPKEPFRD	
39	C076	IRQGPKEPFRDYVDR	
	C077	IKQGPKEPFRDYVDR	
40	C078	PKEPFRDYVDRFFKT	
	C079	PKEPFRDYVDRFYKT	
41	C080	FRDYVDRFFKTLRAE	
	C081	FRDYVDRFYKTLRAE	
42	C082	VDRFFKTLRAEQATQ	
	C083	VDRFYKTLRAEQASQ	
43	C084	FKTLRAEQATQEVKN	
	C085	YKTLRAEQASQEVKN	
44	C086	RAEQATQEVKNWMTD	
	C087	RAEQASQEVKNWMTE	
45	C088	ATQEVKNWMTDTLLV	
	C089	ASQEVKNWMTETLLV	
46	C090	VKNWMTDTLLVQNAN	
	C091	VKNWMTETLLVQNAN	
47	C092	MTDTLLVQNANPDCK	**POOL 3**
	C093	MTETLLVQNANPDCK	**47 peptides**
48	C094	LLVQNANPDCKTILR	
	C095	LLVQNANPDCKTILK	
49	C096	NANPDCKTILRALGP	
	C097	NANPDCKTILKALGP	
50	C098	DCKTILRALGPGATL	
	C099	DCKTILKALGPAATL	
51	C100	ILRALGPGATLEEMM	
	C101	ILKALGPAATLEEMM	

52	C102	LGPGATLEEMMTACQ	
	C103	LGPAATLEEMMTACQ	
53	C104	ATLEEMMTACQGVGG	
54	C105	EMMTACQGVGGPGHK	
	C106	EMMTACQGVGGPSHK	
55	C107	ACQGVGGPGHKARVL	
	C108	ACQGVGGPSHKARVL	
56	C109	KCFNCGKEGHIAKNC	
	C110	KCFNCGKEGHLARNC	
57	C111	CGKEGHIAKNCRAPR	
	C112	CGKEGHLARNCRAPR	
58	C113	GHIAKNCRAPRKRGC	
	C114	GHLARNCRAPRKKGC	
59	C115	KNCRAPRKRGCWKCG	
	C116	RNCRAPRKKGCWKCG	
60	C117	APRKRGCWKCGREGH	
	C118	APRKKGCWKCGKEGH	
61	C119	RGCWKCGREGHQMKD	
	C120	KGCWKCGKEGHQMKD	
62	C121	KCGREGHQMKDCNER	
	C122	KCGKEGHQMKDCTER	
63	C123	EGHQMKDCNERQANF	
	C124	EGHQMKDCTERQANF	
64	C125	MKDCNERQANFLGKI	
	C126	MKDCTERQANFLGKI	
65	C127	NERQANFLGKIWPSH	
	C128	TERQANFLGKIWPSN	
66	C129	ANFLGKIWPSHKGRP	
	C130	ANFLGKIWPSNKGRP	
67	C131	GKIWPSHKGRPGNFL	
	C132	GKIWPSNKGRPGNFP	
68	C133	PSHKGRPGNFLQSRP	
	C134	PSNKGRPGNFPQSRP	
69	C135	GRPGNFLQSRPEPTA	**135+137**
	C137	GRPGNFPQSRPEPSA	
70	C136	NFLQSRPEPTAPP	**136+138**
	C138	NFPQSRPEPSAPP	
71	C139	LPGKWKPKMIGGIGG	**POOL 4**
	C140	LPGRWKPKMIGGIGG	**41 peptides**
72	C141	WKPKMIGGIGGFIKV	
73	C142	MIGGIGGFIKVKQYD	
	C143	MIGGIGGFIKVRQYD	
74	C144	IGGFIKVKQYDQILI	
	C145	IGGFIKVRQYDQIPI	
75	C146	IKVKQYDQILIEICG	
	C147	IKVRQYDQIPIEICG	
76	C148	QYDQILIEICGKKAI	
	C149	QYDQIPIEICGHKAI	
77	C150	ILIEICGKKAIGTVL	
	C151	IPIEICGHKAIGTVL	
78	C152	ICGKKAIGTVLVGPT	
	C153	ICGHKAIGTVLIGPT	
79	C154	KAIGTVLVGPTPVNI	

	C155	KAIGTVLIGPTPVNI	
80	C156	TVLVGPTPVNIIGRN	
	C157	TVLIGPTPVNIIGRN	
81	C158	GPTPVNIIGRNMLTQ	
	C159	GPTPVNIIGRNLLTQ	
82	C160	VNIIGRNMLTQLGCT	
	C161	VNIIGRNLLTQIGCT	
83	C162	GRNMLTQLGCTLNFP	
	C163	GRNLLTQIGCTLNFP	
84	C164	LTQLGCTLNFPISPI	
	C165	LTQIGCTLNFPISPI	
85	C166	GCTLNFPISPIDTVP	
	C167	GCTLNFPISPIETVP	
86	C168	NFPISPIDTVPVTLK	
	C169	NFPISPIETVPVKLK	
87	C170	SPIDTVPVTLKPGMD	
	C171	SPIETVPVKLKPGMD	
88	C172	TVPVTLKPGMDGPRV	
	C173	TVPVKLKPGMDGPKV	
89	C174	TLKPGMDGPRVKQWP	
	C175	KLKPGMDGPKVKQWP	
90	C176	GMDGPRVKQWPLTEE	
	C177	GMDGPKVKQWPLTEE	
91	C178	PRVKQWPLTEEKIKA	
	C179	PKVKQWPLTEEKIKA	
92	C180	QWPLTEEKIKALTEI	**POOL 5**
	C181	QWPLTEEKIKALVEI	**40 peptides**
93	C182	TEEKIKALTEICKEM	
	C183	TEEKIKALVEICTEM	
94	C184	IKALTEICKEMEKEG	
	C185	IKALVEICTEMEKEG	
95	C186	TEICKEMEKEGKITK	
	C187	VEICTEMEKEGKISK	
96	C188	KEMEKEGKITKIGPE	
	C189	TEMEKEGKISKIGPE	
97	C190	KEGKITKIGPENPYN	
	C191	KEGKISKIGPENPYN	
98	C192	ITKIGPENPYNTPIF	
	C193	ISKIGPENPYNTPVF	
99	C194	GPENPYNTPIFAIKK	
	C195	GPENPYNTPVFAIKK	
100	C196	PYNTPIFAIKKKDST	
	C197	PYNTPVFAIKKKDST	
101	C198	PIFAIKKKDSTKWRK	
	C199	PVFAIKKKDSTRWRK	
102	C200	IKKKDSTKWRKLVDF	
	C201	IKKKDSTRWRKLVDF	
103	C202	DSTKWRKLVDFRELN	
	C203	DSTRWRKLVDFRELN	
104	C204	WRKLVDFRELNKKTQ	
	C205	WRKLVDFRELNKRTQ	
105	C206	VDFRELNKKTQDFWE	
	C207	VDFRELNKRTQDFWE	

106	C208	ELNKKTQDFWEVQLG	
	C209	ELNKRTQDFWEVQLG	
107	C210	KTQDFWEVQLGIPHP	
	C211	RTQDFWEVQLGIPHP	
108	C212	FWEVQLGIPHPAGLK	
	C213	FWEVQLGIPHPSGLK	
109	C214	QLGIPHPAGLKKKKS	
	C215	QLGIPHPSGLKKKRS	
110	C216	PHPAGLKKKKSVTVL	
	C217	PHPSGLKKKRSVTVL	
111	C218	GLKKKKSVTVLDVGD	
	C219	GLKKKRSVTVLDVGD	
112	C220	KKSVTVLDVGDAYFS	**POOL 6**
	C221	KRSVTVLDVGDAYFS	**39 peptides**
113	C222	TVLDVGDAYFSVPLD	
114	C224	VGDAYFSVPLDESFR	
	C225	VGDAYFSVPLDKDFR	
115	C226	YFSVPLDESFRKYTA	
	C227	YFSVPLDKDFRKYTA	
116	C228	PLDESFRKYTAFTIP	
	C229	PLDKDFRKYTAFTIP	
117	C230	SFRKYTAFTIPSINN	
	C231	DFRKYTAFTIPSTNN	
118	C232	YTAFTIPSINNETPG	
	C233	YTAFTIPSTNNETPG	
119	C234	TIPSINNETPGIRYQ	
	C235	TIPSTNNETPGVRYQ	
120	C236	INNETPGIRYQYNVL	
	C237	TNNETPGVRYQYNVL	
121	C238	TPGIRYQYNVLPQGW	
	C239	TPGVRYQYNVLPMGW	
122	C240	RYQYNVLPQGWKGSP	
	C241	RYQYNVLPMGWKGSP	
123	C242	NVLPQGWKGSPAIFQ	
	C243	NVLPMGWKGSPAIFQ	
124	C244	QGWKGSPAIFQSSMT	
	C245	MGWKGSPAIFQCSMT	
125	C246	GSPAIFQSSMTKILE	
	C247	GSPAIFQCSMTKILE	
126	C248	IFQSSMTKILEPFRA	
	C249	IFQCSMTKILEPFRK	
127	C250	SMTKILEPFRAKNPE	
	C251	SMTKILEPFRKQNPD	
128	C252	ILEPFRAKNPEIVIY	
	C253	ILEPFRKQNPDIVIY	
129	C254	FRAKNPEIVIYQYMD	
	C255	FRKQNPDIVIYQYMD	
130	C256	NPEIVIYQYMDDLYV	
	C257	NPDIVIYQYMDDLYI	
131	C258	VIYQYMDDLYVGSDL	
	C259	VIYQYMDDLYIGSDL	
132	C260	YMDDLYVGSDLEIGQ	
	C261	YMDDLYIGSDLEIGQ	

133	C262	LYVGSDLEIGQHRAK	**POOL 7**
	C263	LYIGSDLEIGQHRTK	**39 peptides**
134	C264	SDLEIGQHRAKIEEL	
	C265	SDLEIGQHRTKIEEL	
135	C266	IGQHRAKIEELREHL	
	C267	IGQHRTKIEELRQHL	
136	C268	RAKIEELREHLLKWG	
	C269	RTKIEELRQHLLRWG	
137	C270	EELREHLLKWGFTTP	
	C271	EELRQHLLRWGFTTP	
138	C272	EHLLKWGFTTPDKKH	
	C273	QHLLRWGFTTPDKKH	
139	C274	KWGFTTPDKKHQKEP	
	C275	RWGFTTPDKKHQKEP	
140	C276	TTPDKKHQKEPPFLW	
	C277	TTPDKKHQKEPPFHW	
141	C278	KKHQKEPPFLWMGYE	
	C279	KKHQKEPPFHWMGYE	
142	C280	KEPPFLWMGYELHPD	
	C281	KEPPFHWMGYELHPD	
143	C282	FLWMGYELHPDRWTV	
	C283	FHWMGYELHPDKWTV	
144	C284	GYELHPDRWTVQPIQ	
	C285	GYELHPDKWTVQPIV	
145	C286	HPDRWTVQPIQLPEK	
	C287	HPDKWTVQPIVLPEK	
146	C288	WTVQPIQLPEKESWT	
	C289	WTVQPIVLPEKDSWT	
147	C290	PIQLPEKESWTVNDI	
	C291	PIVLPEKDSWTVNDI	
148	C292	PEKESWTVNDIQKLI	
	C293	PEKDSWTVNDIQKLV	
149	C294	SWTVNDIQKLIGKLN	
	C295	SWTVNDIQKLVGKLN	
150	C296	NDIQKLIGKLNWASQ	
	C297	NDIQKLVGKLNWASQ	
151	C298	KLIGKLNWASQIY	
	C299	KLVGKLNWASQIY	
152	C300	AEIQKQGQDQWTYQI	**POOL 8**
	C301	AEIQKQGQGQWTYQI	**42 peptides**
153	C302	KQGQDQWTYQIYQEP	
	C303	KQGQGQWTYQIYQEP	
154	C304	DQWTYQIYQEPFKNL	
	C305	GQWTYQIYQEPYKNL	
155	C306	YQIYQEPFKNLKTGK	
	C307	YQIYQEPYKNLKTGK	
156	C308	QEPFKNLKTGKYA	
	C309	QEPYKNLKTGKYA	
157	C310	FNLPPIVAKEIVANC	
	C311	FNLPPVVAKEIVASC	
158	C312	PIVAKEIVANCDKCQ	
	C313	PVVAKEIVASCDKCQ	
159	C314	KEIVANCDKCQLKGE	

	C315	KEIVASCDKCQLKGE	
160	C316	ANCDKCQLKGEAMHG	
	C317	ASCDKCQLKGEAIHG	
161	C318	KCQLKGEAMHGQVDC	
	C319	KCQLKGEAIHGQVDC	
162	C320	KGEAMHGQVDCSPGI	
	C321	KGEAIHGQVDCSPGI	
163	C322	MHGQVDCSPGIWQLD	
	C323	IHGQVDCSPGMWQLD	
164	C324	VDCSPGIWQLDCTHL	
	C325	VDCSPGMWQLDCTHL	
165	C326	PGIWQLDCTHLEGKV	
	C327	PGMWQLDCTHLEGKV	
166	C328	QLDCTHLEGKVILVA	
	C329	QLDCTHLEGKIILVA	
167	C330	THLEGKVILVAVHVA	
	C331	THLEGKIILVAVHVA	
168	C332	GKVILVAVHVASGY	
	C333	GKIILVAVHVASGY	
169	C334	QEFGIPYNPQSQGVV	**POOL 9**
170	C335	IPYNPQSQGVVESMN	**34 peptides**
171	C336	PQSQGVVESMNKELK	
	C337	PQSQGVVESMNNELK	
172	C338	GVVESMNKELKKIIG	
	C339	GVVESMNNELKKIIG	
173	C340	SMNKELKKIIGQVRE	
	C341	SMNNELKKIIGQVRD	
174	C342	ELKKIIGQVREQAEH	
	C343	ELKKIIGQVRDQAEH	
175	C344	IIGQVREQAEHLKTA	
	C345	IIGQVRDQAEHLKTA	
176	C346	VREQAEHLKTAVQMA	
	C347	VRDQAEHLKTAVQMA	
177	C348	AEHLKTAVQMAVFIH	
	C349	AEHLKTAVQMAVLIH	
178	C350	KTAVQMAVFIHNFKR	
	C351	KTAVQMAVLIHNFKR	
179	C352	QMAVFIHNFKRKGGI	
	C353	QMAVLIHNFKRRGGI	
180	C354	FIHNFKRKGGIGGYS	
	C355	LIHNFKRRGGIGGYS	
181	C356	FKRKGGIGGYSAGER	
	C357	FKRRGGIGGYSAGER	
182	C358	GGIGGYSAGERIIDI	
	C359	GGIGGYSAGERIVDI	
183	C360	GYSAGERIIDIIATD	
	C361	GYSAGERIVDIIATD	
184	C362	GERIIDIIATDIQTK	
	C363	GERIVDIIATDIQTR	
185	C364	IDIIATDIQTKELQK	
	C365	VDIIATDIQTRELQK	
186	C366	ATDIQTKELQKQITK	
	C367	ATDIQTRELQKQIIK	

187	C368	QTKELQKQITKIQNF	**POOL 10**
	C369	QTRELQKQIIKIQNF	**34 peptides**
188	C370	LQKQITKIQNFRVYY	
	C371	LQKQIIKIQNFRVYY	
189	C372	ITKIQNFRVYYRDSR	
	C373	IIKIQNFRVYYRDSR	
190	C374	QNFRVYYRDSRDPIW	
	C375	QNFRVYYRDSRDPLW	
191	C376	VYYRDSRDPIWKGPA	
	C377	VYYRDSRDPLWKGPA	
192	C378	DSRDPIWKGPAKLLW	
	C379	DSRDPLWKGPAKLLW	
193	C380	PIWKGPAKLLWKGEG	
	C381	PLWKGPAKLLWRGEG	
194	C382	GPAKLLWKGEGAVVI	
	C383	GPAKLLWRGEGAVVI	
195	C384	LLWKGEGAVVIQDNS	
	C385	LLWRGEGAVVIQDNS	
196	C386	GEGAVVIQDNSDIKV	
	C387	GEGAVVIQDNSEIKV	
197	C388	VVIQDNSDIKVVPRR	
	C389	VVIQDNSEIKVVPRR	
198	C390	DNSDIKVVPRRKVKI	
	C391	DNSEIKVVPRRKAKI	
199	C392	IKVVPRRKVKIIKDY	
	C393	IKVVPRRKAKIIRDY	
200	C394	PRRKVKIIKDYGKQM	
	C395	PRRKAKIIRDYGKQM	
201	C396	VKIIKDYGKQMAGAD	
	C397	AKIIRDYGKQMAGDD	
202	C398	KDYGKQMAGADCVAG	
	C399	RDYGKQMAGDDCVAS	
203	C400	KQMAGADCVAGRQDED	
	C401	KQMAGDDCVASRQDED	

Figure S3 The tHIVconsvX-derived 15-mer peptides and their pairing and pooling. The tHIVconsvX immunogens contain 2 Gag and 4 Pol conserved regions and are designed as a bi-valent mosaic. This means that with a few exceptions, each of the selected 15-amino acid windows has two versions, which differ in about 10% of amino acids. The altogether 401 overlapping 15-mer peptides are organized into 10 pools such, that each related peptide pair is present in the same pool. This way, individual pool frequencies can be added to estimate the overall magnitude of the T-cell response to the tHIVconsvX conserved regions without counting the same responses twice. Peptides highlighted blue have no variant pair; these are C047, C104, C141, C222, C334, C335. Peptides highlighted yellow do not follow in sequential order of its variant.

C031	GHQAAMQMLKDTINE	C262	LYVGSDLEIGQHRAK
C032	GHQAAMQMLKETINE	C263	LYIGSDLEIGQHRTK
C033	AMQMLKDTINEEAAE	C270	EELREHLLKWGFTTP
C034	AMQMLKETINEEAAE	C271	EELRQHLLRWGFTTP
C062	PVGDIYKRWIILGLN	C272	EHLLKWGFTTPDKKH
C063	PVGEIYKRWIIMGLN	C273	QHLLRWGFTTPDKKH
C082	VDRFFKTLRAEQATQ	C350	KTAVQMAVFIHNFKR
C083	VDRFYKTLRAEQASQ	C351	KTAVQMAVLIHNFKR
C154	KAIGTVLVGPTPVNI	C352	QMAVFIHNFKRKGGI
C155	KAIGTVLIGPTPVNI	C353	QMAVLIHNFKRRGGI
C156	TVLVGPTPVNIIGRN	C372	ITKIQNFRVYYRDSR
C157	TVLIGPTPVNIIGRN	C373	IIKIQNFRVYYRDSR
C180	QWPLTEEKIKALTEI	C374	QNFRVYYRDSRDPIW
C181	QWPLTEEKIKALVEI	C375	QNFRVYYRDSRDPLW
C246	GSPAIFQSSMTKILE	C376	VYYRDSRDPIWKGPA
C247	GSPAIFQCSMTKILE	C377	VYYRDSRDPLWKGPA
C248	IFQSSMTKILEPFRA		
C249	IFQCSMTKILEPFRK		

Figure S4 The BALB/c pool of peptides recognized by T cells induced by a single dose saRNA.

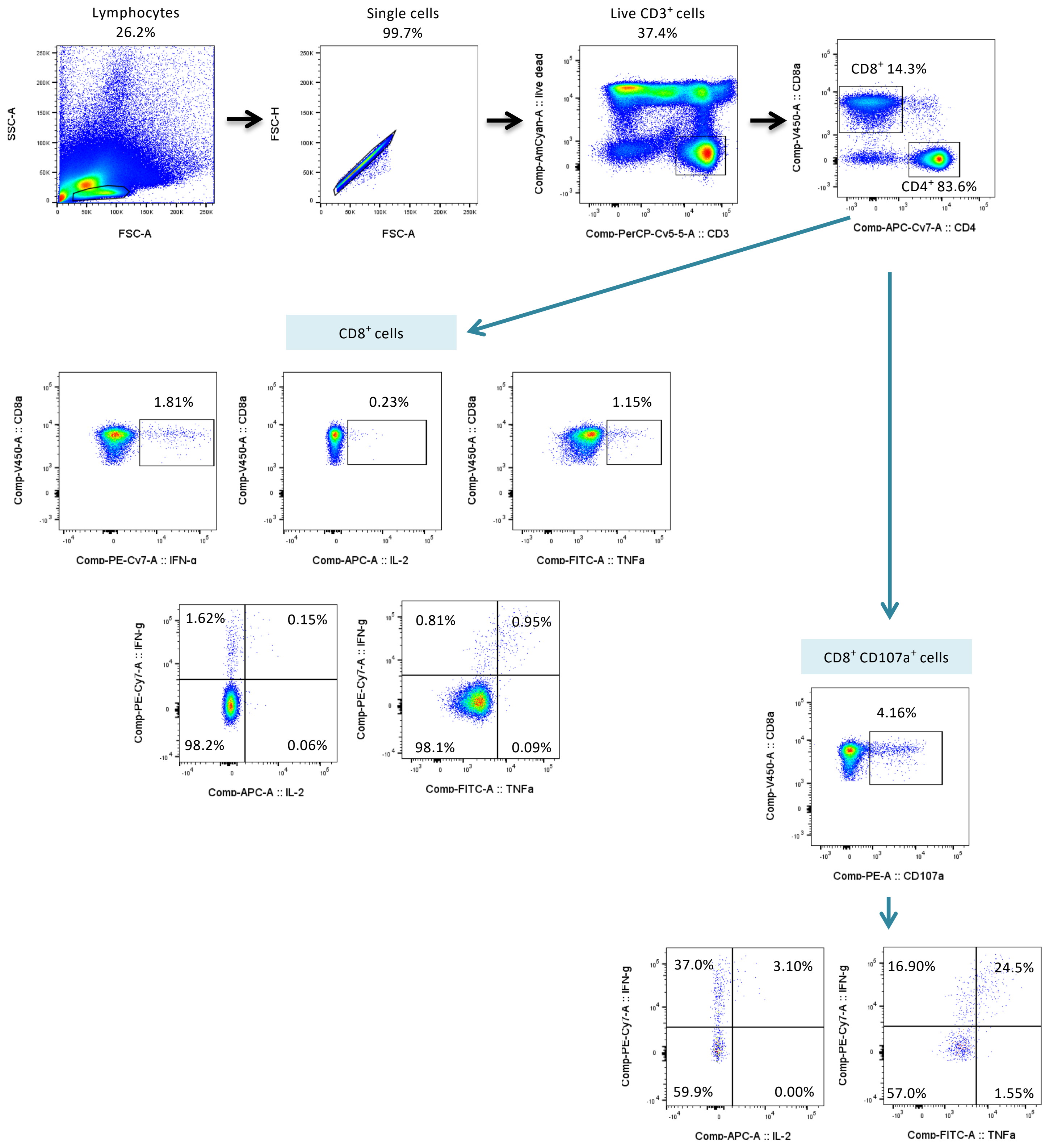

Figure S5 Gating strategy for the T-cell functional phenotype analysis.

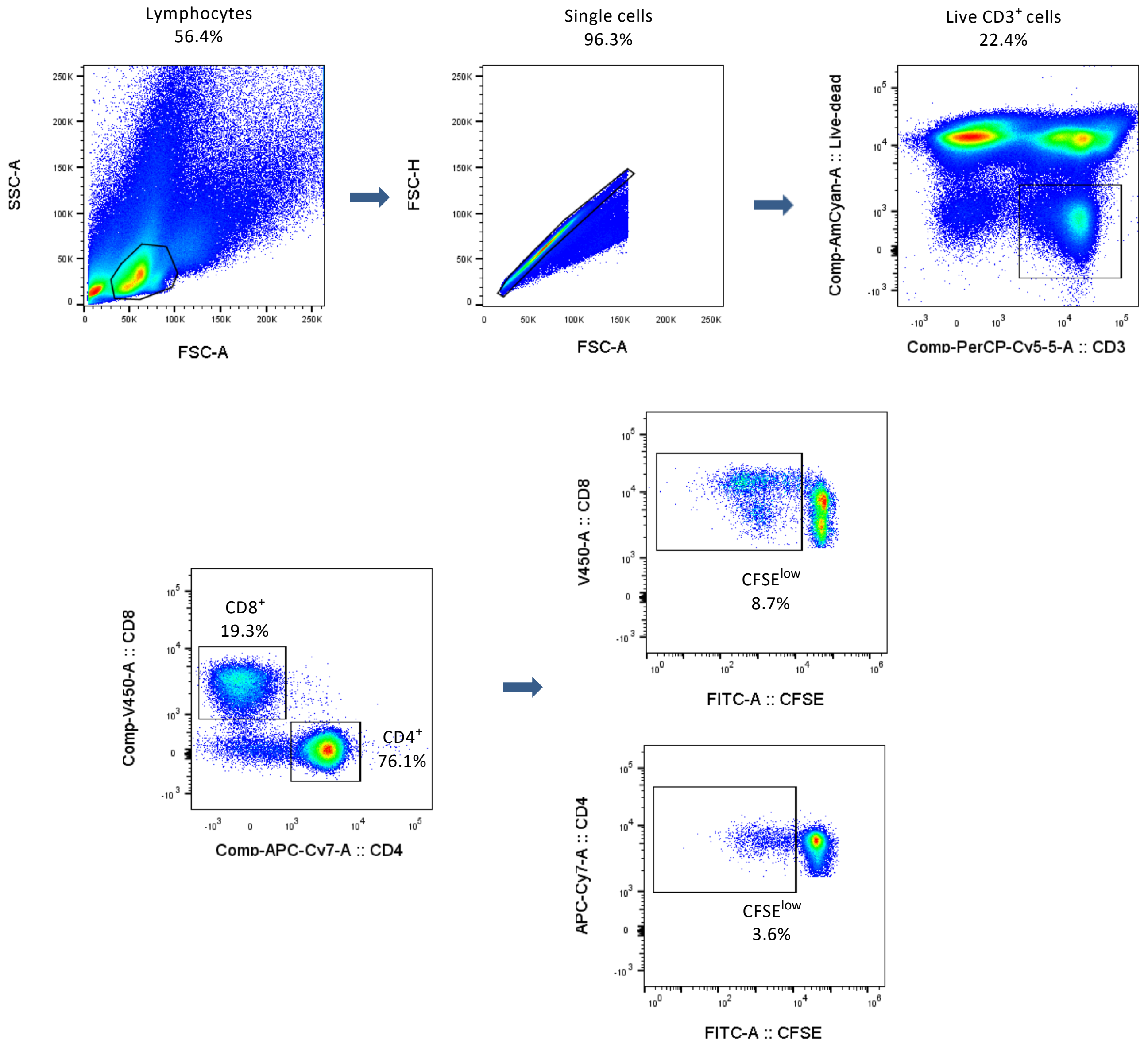

Figure S6 Gating strategy for the CFSE proliferation assay.

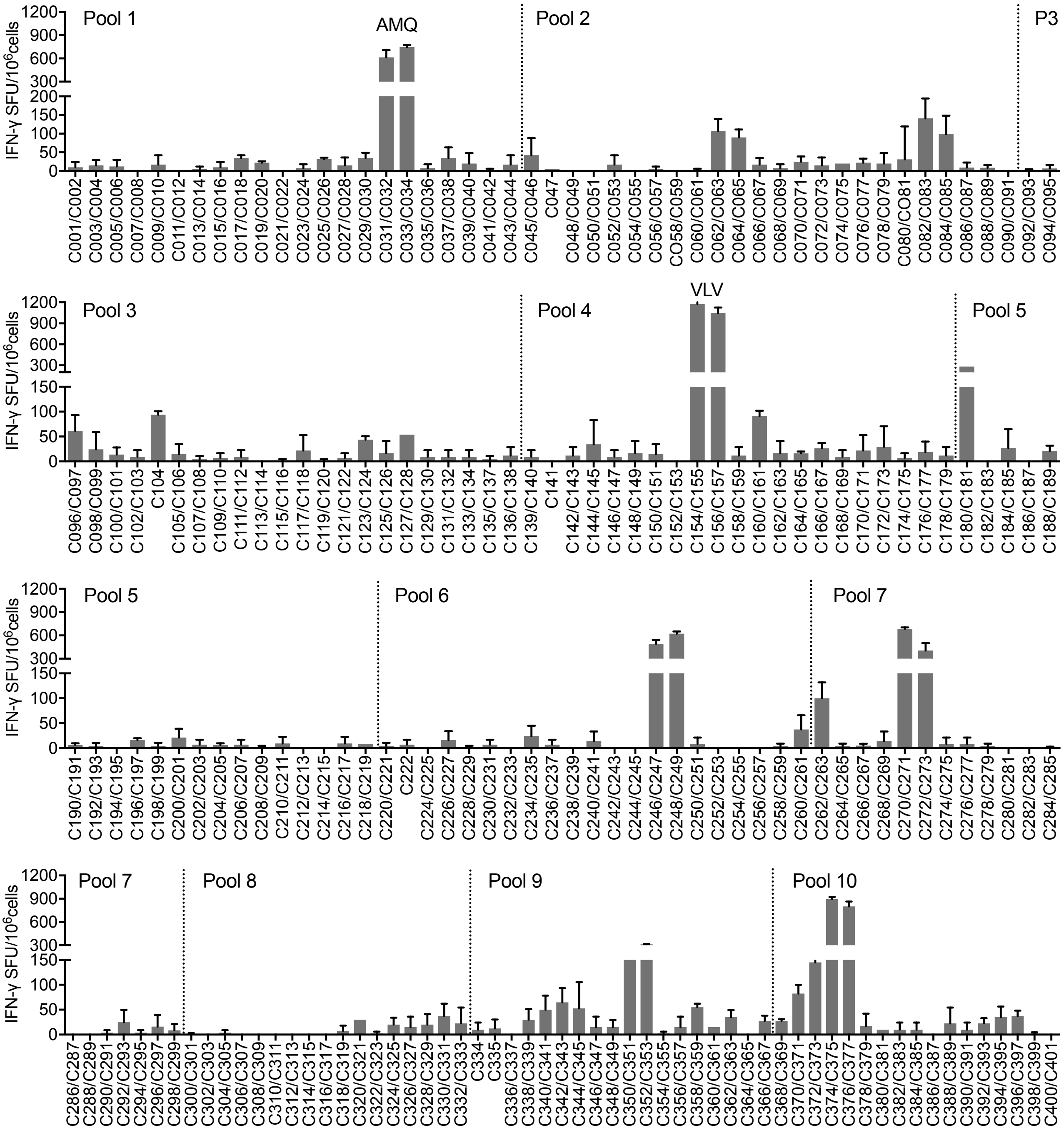

Figure S7 RNA-MVA-elicited responses to individual peptide pairs across the tHIVconsvX immunogens. Groups of BALB/c mice were immunized with AIR.tHIVconsv1+AIR.tHIVconsv2 saRNA followed by MVA.tHIVconsv3+MVA.tHIVconsv4, sacrificed 1 week later and their splenocytes were assessed in an IFN-γ ELSPOT assay using individual 15-mer peptide pairs, whereby one peptide was from mosaic 1 and the other peptide was derived from mosaic 2 (See Figure S3 for the peptide sequences).

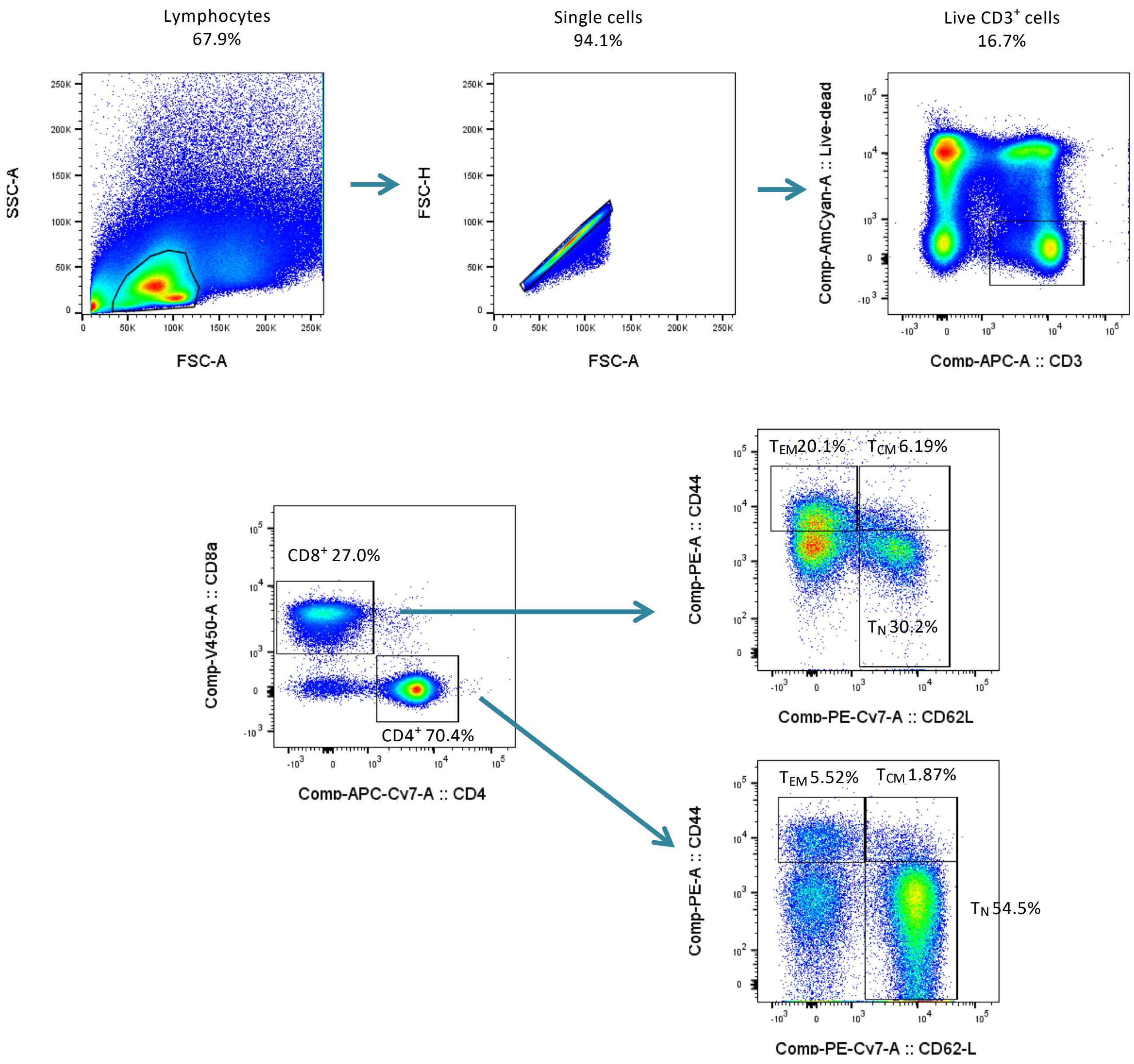

Figure S8 Gating strategy for the T-cell memory subtype analysis.
